# RNA Secondary Structurome Revealed Distinct Thermoregulation in *Plasmodium falciparum*

**DOI:** 10.3389/fcell.2021.766532

**Published:** 2022-01-04

**Authors:** Yanwei Qi, Yuhong Zhang, Quankai Mu, Guixing Zheng, Mengxin Zhang, Bingxia Chen, Jun Huang, Changling Ma, Xinhua Wang

**Affiliations:** ^1^ Department of Pathogenic Biology and Immunology, School of Basic Medical Sciences, Guangzhou Medical University, Guangzhou, China; ^2^ Department of Immunogenetics, Institute of Tropical Medicine (NEKKEN), Nagasaki University, Nagasaki, Japan; ^3^ Department of Blood Transfusion, The First Affiliated Hospital of Guangzhou Medical University, Guangzhou Medical University, Guangzhou, China; ^4^ The Third Clinical School, Guangzhou Medical University, Guangzhou, China; ^5^ The First Affiliated Hospital of Guangzhou Medical University, Guangzhou Medical University, Guangzhou, China

**Keywords:** *Plasmodium falciparum*, RNA secondary structure, RNA thermometers, *in vivo*, *in vitro*, mRNA abundance

## Abstract

The development of *Plasmodium* parasites, a causative agent of malaria, requests two hosts and the completion of 11 different parasite stages during development. Therefore, an efficient and fast response of parasites to various complex environmental changes, such as ambient temperature, pH, ions, and nutrients, is essential for parasite development and survival. Among many of these environmental changes, temperature is a decisive factor for parasite development and pathogenesis, including the thermoregulation of rRNA expression, gametogenesis, and parasite sequestration in cerebral malaria. However, the exact mechanism of how *Plasmodium* parasites rapidly respond and adapt to temperature change remains elusive. As a fundamental and pervasive regulator of gene expression, RNA structure can be a specific mechanism for fine tuning various biological processes. For example, dynamic and temperature-dependent changes in RNA secondary structures can control the expression of different gene programs, as shown by RNA thermometers. In this study, we applied the *in vitro* and *in vivo* transcriptomic-wide secondary structurome approach icSHAPE to measure parasite RNA structure changes with temperature alteration at single-nucleotide resolution for ring and trophozoite stage parasites. Among 3,000 probed structures at different temperatures, our data showed structural changes in the global transcriptome, such as S-type rRNA, HRPII gene, and the erythrocyte membrane protein family. When the temperature drops from 37°C to 26°C, most of the genes in the trophozoite stage cause significantly more changes to the RNA structure than the genes in the ring stage. A multi-omics analysis of transcriptome data from RNA-seq and RNA structure data from icSHAPE reveals that the specific RNA secondary structure plays a significant role in the regulation of transcript expression for parasites in response to temperature changes. In addition, we identified several RNA thermometers (RNATs) that responded quickly to temperature changes. The possible thermo-responsive RNAs in *Plasmodium falciparum* were further mapped. To this end, we identified dynamic and temperature-dependent RNA structural changes in the *P. falciparum* transcriptome and performed a comprehensive characterization of RNA secondary structures over the course of temperature stress in blood stage development. These findings not only contribute to a better understanding of the function of the RNA secondary structure but may also provide novel targets for efficient vaccines or drugs.

## Introduction

A precise and quick response to environmental change is essential for *Plasmodium falciparum* development and the pathogenesis of the disease. A previous study found that parasites can sense metabolism and the environment using a variety of regulatory reprogramming mechanisms; for example, parasites alter the expression of enzymes involved in glycolysis and gluconeogenesis gene to sensitize the glucose (Zuzarte-Luis and Mota, 2018). Recent studies (Mustoe et al., 2018) have established that the RNA secondary and tertiary structures act as fundamental and pervasive regulators of individual transcript expression within seconds (Ganser et al., 2019). The control of gene expression in organisms through structural transformation of RNA elements can promote rapid responses to changing environmental conditions and enhance regulation at the transcription level (Choi et al., 2017). For example RNA thermometers (RNATs), which are usually located in the 5′-UTR region, can respond to changes in temperature within seconds and directly control nascent or existing mRNA translation. These factors have an instantaneous effect on the expression of transcripts related to temperature stress and allow for a fast, cost-effective, and potentially reversible response (Kortmann and Narberhaus, 2012). Thermo-responsive RNA, which alters the RNA structure in response to temperature changes, has recently been studied in other organisms, such as bacteria (Righetti and Narberhaus, 2014; Righetti et al., 2016; Abduljalil, 2018) and yeast (Kertesz, 2010; Talkish et al., 2014). However, all these elements were discovered by the *in vitro* RNA-probing approach.

Malaria, a deadly parasitic disease with high mortality and morbidity rates, caused approximately 229 million cases and around 409,000 deaths in 2019 (World Health Organization, 2020). Without effective vaccines, and the combination of drug and insecticide resistance, it has become an urgent issue to discover and develop new effective strategies to control malaria. Among many of these environmental changes, it has been shown that temperature is a decisive factor for parasite development and pathogenesis, including the thermoregulation of rRNA expression, gametogenesis, and parasite sequestration in cerebral malaria (Singhaboot et al., 2019). Parasites are exquisitely responsive to human host body temperature (37°C), which induces the most metabolic shifts, while the most suitable temperature for the developmental stage of the parasites in mosquitoes is 26°C. Unlike most eukaryotes, *P. falciparum* has two distinct types of 18S ribosomal RNA genes, one type is primarily expressed in the mammalian host (A gene) and the other is primarily expressed in the mosquito host (S gene) (Rogers et al., 1996). *Plasmodium* 18S rRNA has been shown to be developmentally regulated, and the change of temperature represents a predominant factor associated with transcriptional control of S-type rRNAs and the other rRNA copies (Fang and McCutchan, 2002; Fang et al., 2004).

Despite the importance of the temperature response in parasite biology, the precise mechanism underlying this response is still exclusive; thus, the molecular mechanisms in the RNA structure responsible for these reactions are an important yet missing piece of the puzzle. The major strengths of this study are the discussion on parasite development by temperature through RNA secondary structure and our analysis profiles of the structure of transcripts in trophozoite and ring stages after cold shock (low temperature). Here, we applied icSHAPE to profile the transcriptomic-wide *in vivo* and *in vitro* RNA secondary structures. Low icSHAPE scores mean that the position is more structured or presents a protein-binding site of the structure. The flexibility of the RNA secondary structure measured by icSHAPE score indicates the probability that a nucleotide is present in unpaired or single-stranded forms. A combined analysis of these structural profiles and transcriptome data reveals a unique RNA secondary structure that responds to temperature changes and parasite stage development, including S-type rRNA, parasite-infected erythrocyte surface protein (PIESP2), and erythrocyte membrane protein.

Our results provide a framework for understanding how the malaria parasite responds to its environmental temperature through changes in RNA structure *in vivo* and indicate that the development of *P. falciparum* is closely connected to the RNA secondary structure even in *P. falciparum* after cold shock. However, the mechanism needs to be further explored. A greater understanding of the structure and function of RNAT response to changes in temperature in the 5′-UTR region could thus provide insights into the virulence and development of *P. falciparum*.

## Materials and Methods

### Parasite Culture and Temperature Treatment

As described previously, *P. falciparum* strain 3D7 parasites were propagated in human red blood cells under standard conditions (Trager and Jensen, 1976). Parasites were synchronized twice within 8 h apart by 5% D-sorbitol treatments (Lambros and Vanderberg, 1979). When the parasites in 5% hematocrit reached ∼8% parasitemia, they were harvested and exposed to two temperatures (37°C and 26°C) at 45 and 70 h after the first sorbitol treatment, respectively. The asexual parasite cultures were incubated for a continued 3 h at the two different temperatures. Three hours later, the culture was harvested for 3 min at 2,000 rpm to obtain RBCs. Then, the parasites, which were in the synchronous ring and trophozoite stages, were lysed with 0.05% saponin solution, followed by washing parasite pellets using PBS (pH 7.4). The remaining parasite pellets were used for total RNA extraction or NAI-N_3_ *in vivo* modification.

### *In Vitro* and *In Vivo* NAI-N_3_ Probing and RNA Sample Extraction

For *in vivo* NAI-N_3_ modification, the washed parasite pellets were subjected to *in vivo* NAI-N_3_ modification procedures according to standards (Flynn et al., 2016) using 100 mM NAI-N_3_ for 15 min as described previously (Qi et al., 2021) before total RNA extraction. Briefly, after lysis of RBCs by 0.05% saponin treatment and washing with PBS, the parasite pellet was resuspended in 200 µl of dimethyl sulfoxide (DMSO) solution or 100 mM NAI-N_3_ solution, mixed by inversion and incubated at 37°C for 15 min. These modifications cause reverse transcription to stop one nucleotide before modification. During NAI-N_3_ modification, we set a DMSO-treated negative control sample because the DMSO sample can provide an “input” sample. The reaction was stopped and collected at 14,000 *g* for 30 s at 4°C. Then, the pellet was resuspended in 10 ml of pre-warmed TRIzol, and total RNA was isolated followed by DNase treatment. For the icSHAPE *in vitro* libraries, heat-denatured total RNA or polyA-selected RNA samples from parasites were treated with DMSO solution at 95°C for 2 min and then transferred to ice for cooling. The time and concentration of modified NAI-N_3_ *in vitro* were 10 min at 37°C and 100 mM, respectively. Finally, the samples were transferred to ice to stop the reaction, and the RNA sample was purified by a Zymo RNA Clean and Concentrator-5 column.

### RNA-Seq and icSHAPE Illumina Library Construction and Sequencing

Validated strand-specific, polyA-selected RNA sequencing libraries were prepared with the NEBNext Ultra Directional RNA Library Prep Kit (NEB) according to the manufacturer’s instructions and as described previously (Qi et al., 2021). These libraries were sequenced on an Illumina HiSeq 2000 at GENEWIZ (Suzhou, China). Finally, read counts were collected, and gene expression levels were estimated. The edgeR package was used to identify differentially expressed genes across different groups. The *in vitro* and *in vivo* icSHAPE libraries were generated as previously described (Flynn et al., 2016; Qi et al., 2021). Finally, the PCR-amplified library with a size between 200 and 300 bp was selected by 10% TBE-PAGE. These icSHAPE libraries were quantified on a BioAnalyzer High Sensitivity DNA Chip 2100 (Agilent) and Qubit. Then, the samples were sent to GENEWIZ (Suzhou, China), or Vazyme Biotechnology Co., Ltd.’s (Nanjing, China) Illumina HiSeq was used for deep sequencing.

### Quantifying the Modification of 18S rRNA by Primer Extension, Resolved by Capillary Electrophoresis

Total RNA was extracted from parasite lysates with NAI-N_3_ chemical reagent modification as described above. In total, 7 μg RNA without poly-A selected from the 37°C control group and the 26°C treated group in the trophozoite developmental stage was digested with RNase-free DNase to remove residual DNA. Then, these total RNAs were reverse transcribed by primer extension with a FAM-labelled oligonucleotide (5′- ACC​CTA​ACA​TCA​AAA​GCT​GAT​AGG -3′) in the 315–338-bp region of the 18S rRNA gene. The PCR program was as follows: 5 min at 65°C, then kept on ice for 2 min, followed by 1 h at 50°C and 5 min at 95°C, and finally, the sample was kept at 4°C when ending the reaction. The reverse-transcribed products were resolved by capillary electrophoresis (CE) using an ABI 3730XL DNA Sequencer, and the results were shown by GeneMarker.

### Quantitative Real-Time PCR

Total RNA used for the qPCR analysis was isolated and purified as described above. Briefly, approximately 2 μg of the RNA sample was used for strand complementary DNA synthesized by either oligo dT primers or random primer mixes using a Superscript III Reverse Transcriptase kit. Real-time PCR amplification was carried out in the presence of 12.5 µl of SYBR master mixture (Takara Bio Inc.), 1 µl of cDNA template, and target gene-specific primers in a CFX96 System under the following conditions: 1) 1 min at 95°C; 2) 5 s at 95°C; 3) 30 s at 50°C; 4) 30 s at 72°C with a plate reader; and 5) repeat steps 2), 3), and 4) for another 39 cycles. The housekeeping gene, serine–tRNA ligase, putative (PF3D7_0717700), was used to normalize the gene transcriptional level of each sample. Primers for detecting different transcripts from the 37°C control group and the 26°C treatment group were designed in this study. The primer sequences for PCR are listed in Supplementary Table S1.

### Sequencing Data Analysis and RNA Secondary Structure Predictions With icSHAPE Scores

Data analysis was performed as previously described (Qi et al., 2021). The quality of the PF data was evaluated by FastQC (Babraham Bioinformatics), and the percentage of readings with GC content, Q30, and Q20 was calculated. Clean data with the barcode (1–13 bases in each read) removed by Trimmomatic (Bolger et al., 2014) were used for the following analyses. Then, these clean data were mapped to the transcriptome and genome of *P. falciparum* using Bowtie (Langmead et al., 2009) with default parameters. Finally, to reduce the overestimation of signals of individual bases with zero or low coverage (Wan et al., 2014), a value of 5 was added. After normalizing the reverse transcription stops by the amount of all reads in each library, the raw icSHAPE signals/scores for each RNA position were calculated by the ratio of NAI-N_3_/DMSO numbers of modified nucleotides in each position (Qi et al., 2021). The final analysis of the icSHAPE scores of individual positions was performed with Microsoft Excel. In our study, online Vienna RNA Web Services were used for the color coding by icSHAPE scores. RNAstructure 6.1 (Lu et al., 2009) was used for the RNA secondary structure profiling on a Windows operating system. We concluded that the RNAstructure software threshold parameter for the chemical modification was 1.0 and that for single-stranded force was 1.5. Alternatively, the webserver http://rna.urmc.rochester.edu/RNAstructureWeb/was also used, and finally, the RNA secondary structure was drawn and colored using StructureEditor.

## Results

### Overview of the *P. falciparum* RNA Structurome at Different Temperatures

Previously, we applied icSHAPE to characterize and predict the RNA secondary structure in *P. falciparum* (Qi et al., 2021). In previous analyses, we predicted 3,396 transcripts in trophozoite and ring stages at normal culture temperature (37°C) and identified some key regulatory features. Additionally, our study indicates that the mRNA abundance of *P. falciparum* during the development of parasites is crucially connected to the RNA secondary structure.

**FIGURE 1:**
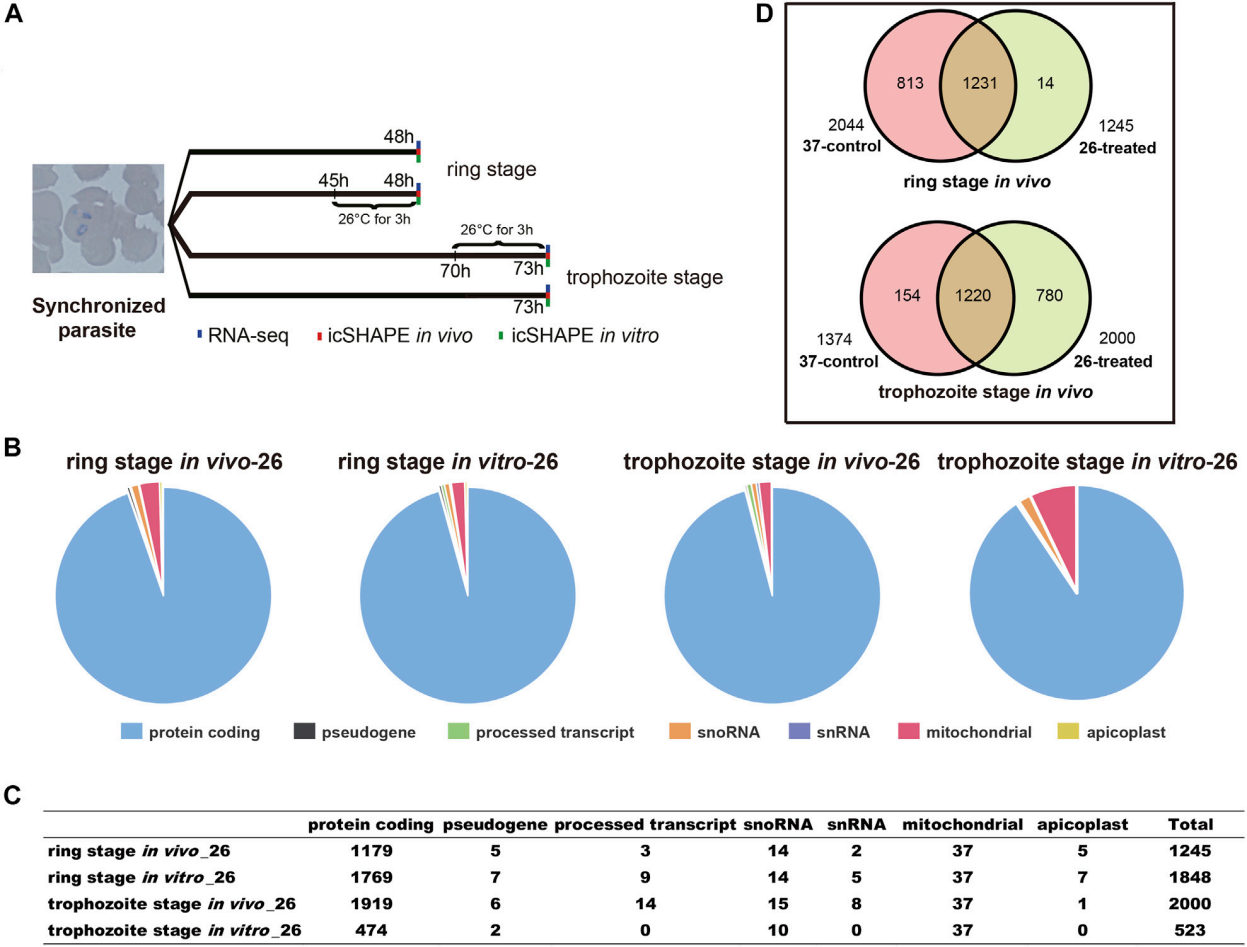
Experimental design and icSHAPE library statistics. **(A)** Overview of the icSHAPE approach in our experiment. We performed temperature stress (37°C and 26°C) to the cultured parasites at 45 h (ring stage)/70 h (trophozoite stage) and continue to culture at different temperatures for continuous 3 h. *In vivo* RNA structure experiments, *in vitro* RNA structure experiments, and RNA-seq experiments were performed for all four groups. **(B, C)** The number of RNA structural profiles for the whole transcripts was classified into various classes of RNAs from 1,245 transcripts for ring library at 26°C *in vivo*, 2,000 transcripts for trophozoite library at 26°C *in vivo*, 1,848 transcripts for ring library at 26°C *in vitro*, and 523 transcripts for trophozoite library at 26°C *in vitro*. **(D)** Overlap of mRNAs between 37°C and 26°C among the two different stages *in vivo*.

To search for potential RNA thermometers in cold shock, we performed icSHAPE experiments with a poly-A-depleted RNA library using new parasites collected from the ring stage and trophozoite stage developmental cycles at a vector host body temperature of 26°C as illustrated in Figure 1A. icSHAPE libraries were generated from the cultured parasites 45/70 h after the first synchronization and with a brief (3 h) treatment at 37°C (control) or 26°C (cold shock) with or without NAI-N_3_ (Figure 1A). The primers used in library construction are listed in Supplementary Table S1. To test whether our icSHAPE accurately probed the RNA structure, we first confirmed the rRNA structure obtained with the traditional method as reported previously (Wong et al., 2014; Qi et al., 2021). We found a high agreement between our icSHAPE signal and the known secondary structure of A-type 18S rRNA. This finding suggests that our genome-wide icSHAPE recapitulates results obtained by CE methods and provides structural information on genome wide transcripts.

We performed 12 icSHAPE experiments (two biological replicates for each stage and condition) with poly-A-depleted RNA library of ring and trophozoite stages, resulting in an average of 46.1 million sequence reads that map to the *P. falciparum* genome. Mapping statistics are detailed in Supplementary Table S2. We defined the ratio of NAI-N_3_/DMSO as numbers of nucleotides with modification and calculated icSHAPE scores for each nucleotide of the transcriptome. The transcripts whose coverage rate was no less than 2 were analyzed as described (Spitale et al., 2015). Finally, 1,245 and 2,000 sufficient structural profiles for transcripts were acquired *in vivo* from the ring stage and trophozoite stage 26°C-treated RNA libraries (with poly-A selected), respectively, and the majority of transcripts were mRNAs (Figure 1B, C and Supplementary Table S3). Additionally, 1,848 and 523 sufficient structural profiles were acquired *in vitro* from the ring stage and trophozoite stage 26°C-treated libraries, respectively (Figure 1B, C and Supplementary Table S3). At last, we obtained sufficient structural coverage at two temperatures for 1,231 mRNAs in the ring stage and 1,220 mRNAs in the trophozoite stage *in vivo*. Finally, these results afforded a transcriptomic-wide view of the RNA secondary structure landscape of *P. falciparum* after cold shock (Figure 1D and Supplementary Table S3).

### Temperature Effects on the Development of Parasites and Temperature-Responsive Gene Expression Determined by RNA-Seq Analysis

The twice-synchronized *P. falciparum* 3D7 was propagated at 37°C to keep most of the parasites at a similar stage (Supplementary Figure S1). To culture the parasites, they were exposed to different temperature stresses (37°C and 26°C) for 45/70 h after the first synchronization and maintained at different temperatures for 3 h as previously described (Fang and McCutchan, 2002; Bunnik et al., 2013). Parasitemia and parasite morphology at each time point were monitored daily by examination of smears made by Giemsa staining. A comparison of the growth status of *P. falciparum* at 26°C and 37°C for three continuous hours of incubation showed that the gametocytemia was higher and the growth rate was slower at 26°C than at 37°C (Supplementary Figure S1). Our findings showed that ambient temperatures may affect the multiplication rate of *P. falciparum*, thus confirming that thermoregulation is crucial to the developmental transition of parasites from vertebrate hosts to mosquito vectors.

**FIGURE 2:**
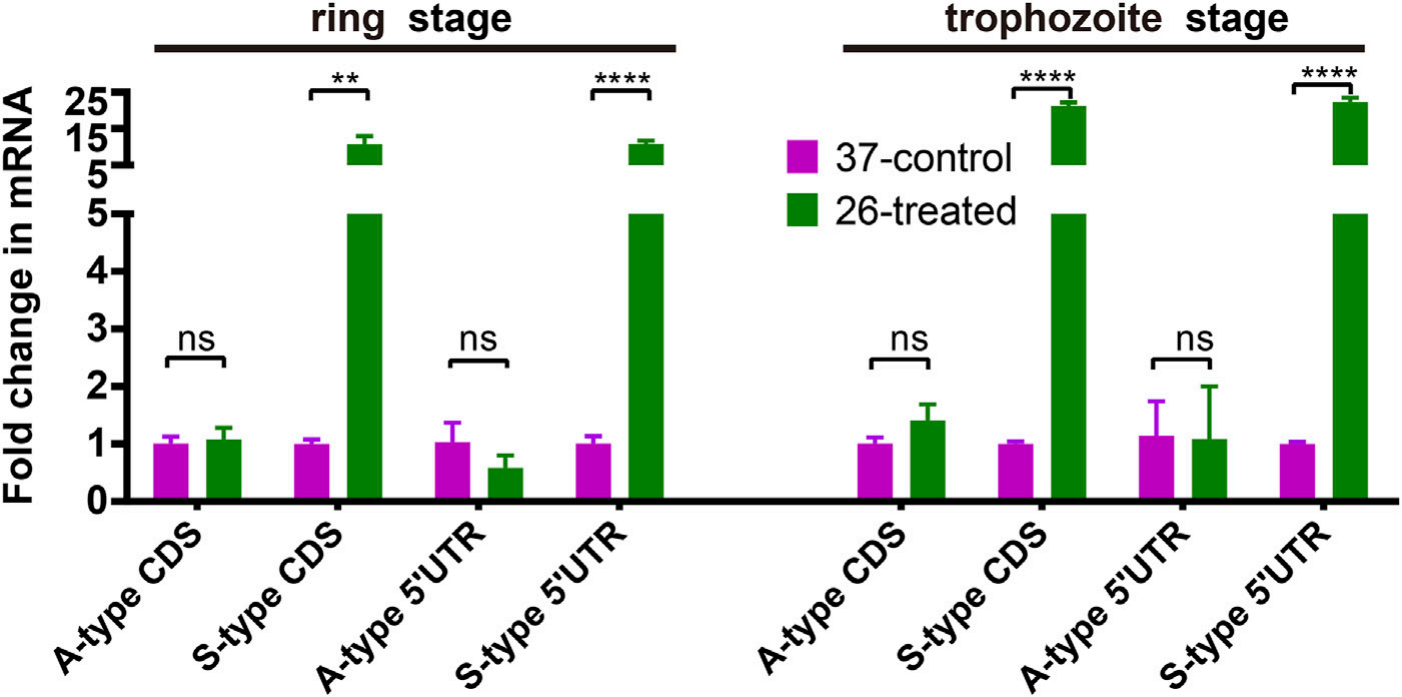
The effect of temperature on the expression of A- and S-type 18S ribosomal RNA (18S rRNA). Fold changes in the expression of 18S ribosomal RNA in the 26°C treatment group was detected by qRT-PCR. qPCR analyses of transcriptional regulation of A- and S-type 18S rRNA (fold changes of 3 h at 26°C to 37°C temperature stimulation) at 45/70 h after invasion (ring stage/trophozoite stage). Stages of parasites (ring stage or trophozoite stage) is shown at the top. Serine–tRNA ligase (PF3D7_0717700), a housekeeping gene, was used to normalize the level of transcription of each gene. Each type of 18S rRNA was detected by two pairs of primers (Supplementary Table S2) located at different sites of transcriptions (5′-UTR and CDS region). The name of each gene is shown at the bottom. The data are representative of three independent experiments. Two-sided *t*-test, ***p < 0.001; ****p < 0.0001; ns, no significance (*t*-test; SDs from three replicates).

To dissect the effect of cold shock on gene expression in *P. falciparum*, we first investigated the expression of A- and S-type 18S ribosomal RNA (18S rRNA). As shown in Figure 2, we compared the expression of A-type and S-type ribosomal RNA at two stages of parasites with a low culture temperature for 3 h. These results showed that after low temperature treatment, the expression level of S-type rRNA in the 26°C treatment group has a significant increase than the 37°C control group whether it happened in the ring stage or trophozoite stage, while the A-type rRNA showed little or no change. This expression pattern was found not only in the CDS region but also in the 5′-UTR region. The DNA primers used for the real-time quantitative PCR (qRT-PCR) assay are listed in Supplementary Table S2.

**FIGURE 3:**
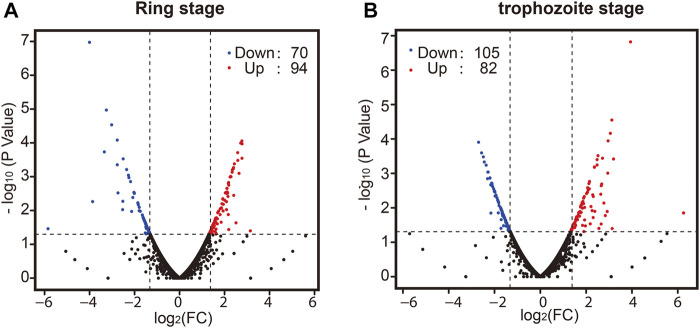
The effect of temperature on the development of parasites in the ring stage and trophozoite stage. Pair-wise comparisons between *Plasmodium* cultures. Volcano plot of log_2_ fold change in gene expression in the 26°C treatment group versus the 37°C control group that differs significantly (|log_2_ratio| ≥ 1 and *q*-value|FDR ≤ 0.05) in the ring stage **(A)** and trophozoite stage **(B)**. Red plots indicate genes upregulated in the 26°C treatment group compared to the 37°C control group; blue plots indicate genes downregulated in the 26°C treatment group compared to the 37°C control group; and black plots indicate genes that are not significantly different between the two groups. A full list of differentially expressed genes for each condition is shown in Supplementary Table S3.

Finally, we sought to determine whether there was a difference in whole-transcriptome expression between different groups. Changes in *P. falciparum* gene expression and regulation in the ring stage and trophozoite stage were investigated by changing the culture temperature, and four samples from the parasite culture are illustrated in Figure 1A. The detailed RNA-seq data are displayed in Supplementary Table S2. A correlation analysis of the whole-transcriptome expression showed good reproducibility between the two biological replicates (*R* ≥ 0.937, Supplementary Figure S2C). We compared the expression levels of differentially expressed genes (DEGs) from the ring stage and trophozoite stage at different culture temperatures for 3 h, and the results showed that a large number of genes had different expressions after cold shock in different groups (Figure 3). A comparison of the ring-stage parasites in the 37°C groups with the ringstage parasites with low temperature treatment showed significant differences in 164 genes (Figure 3A and Supplementary Table S4) between the two temperatures. These genes included downregulation genes, such as erythrocyte membrane protein 1, *Plasmodium* export protein, and rifin (RIF), and upregulation genes, such as ABC transporter G family member 2 and circumsporozoite (CS) protein (CSP). At the same time, when we compared parasites at the trophozoite stage with the 37°C control group parasites, 82 genes had higher expression and 105 genes had lower expression in the 26°C treatment group. A few examples of genes that were obviously expressed at lower level in the 26°C treatment group are erythrocyte membrane protein 1, early transcribed membrane protein, and nine genes encoding *Plasmodium* export protein (Figure 3B and Supplementary Table S4). Other genes such as the genes encoding erythrocyte membrane protein 1, *Plasmodium* export protein, and sporozoites and 22 genes encoding RIF protein were expressed at higher levels in the 26°C-treated parasites.

Interestingly, 38 genes were up-regulated in both stages, and most of the cold-upregulated genes were dominantly expressed in the gametophyte or mosquito stage. For example, CPW-WPC family protein (PF3D7_1331400) is translationally activated during ookinete formation and silenced in gametocytes (Rao et al., 2016). *P. falciparum* LCCL domain-containing protein (PfCCp) proteins (PF3D7_1407000) are expressed in the parasitophorous vacuole and are later associated with macrogametes (Simon et al., 2009). Using the same criteria to identify DEGs, a large number (1,082 and 1,126) of temperature-response genes were found when we compared the ring stage and trophozoite stage at the same culture temperatures 37°C and 26°C (Supplementary Figure S2A, B and Supplementary Table S4). Altogether, these data imply that low temperature-treated parasites display variations in the expression of several genes with important functions in malaria growth and that parasites can respond to the temperature environment by reducing their transmission and replicative fitness. The functional roles of the majority of DEGs in the temperature response are currently unknown and require further investigation.

### The Effect of Specific RNA Structures and Control of the Expression Level of rRNA

**FIGURE 4:**
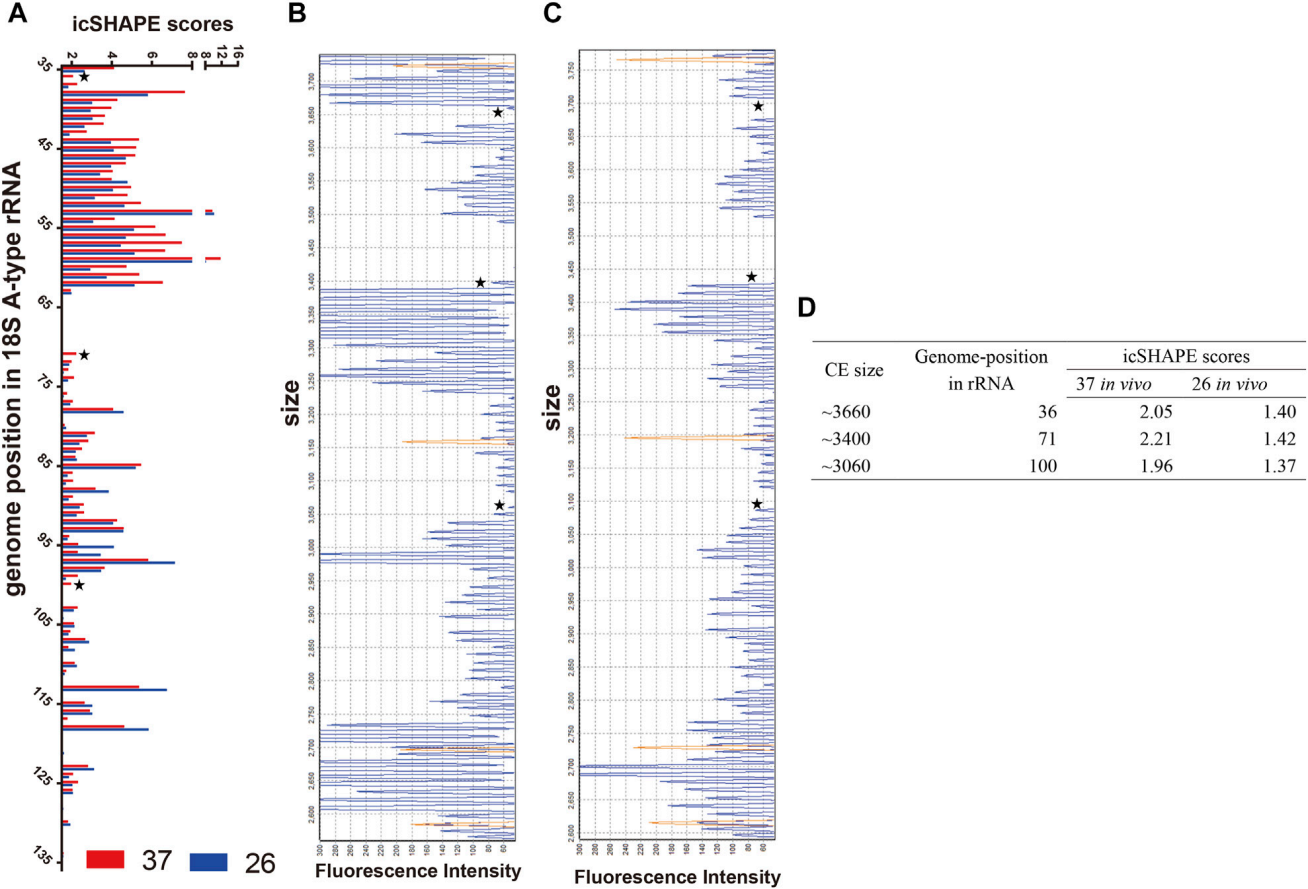
icSHAPE scores for the 5′-end of the 18S A-type ribosome RNA from the 37°C control group and the 26°C treatment group in the trophozoite developmental stage combined with CE gel results. **(A)** icSHAPE scores from 37°C to 26°C groups for nucleotides 35–135 are shown on the left. Those two gels from 18S A-type rRNA of the 37°C control group **(B)** and the 26°C treatment group **(C)** with NAI-N_3_ modification were read out by CE experiment. For those two panels of capillary electrophoresis, the starting RNA material was the same as the total *in vivo* NAI-N_3_-modified RNA. The orange traces in **(B, C)** is GeneScan 500 LIZ Size Standard for capillary electrophoresis gel (from top to bottom: 250, 200, 160, and 150 bp), and the stars represent the three genome positions in 18S A-type rRNA, which are significantly different between the 37°C control group and the 26°C treatment group. **(D)** The icSHAPE scores, CE size, and the position in the rRNA genome from three different base-pair positions.

First, we compared the icSHAPE coverage of A-type and S-type rRNA in the non-depleted RNA libraries, and the results showed that icSHAPE scores from those libraries have a good sequencing depth, especially A2-type 18S rRNA (PF3D7_0531600) and S2-type 18S rRNA (PF3D7_1371000) (Supplementary Table S5). To accurately obtain the RNA secondary structure, we chose these two 18S rRNAs to study the direct temperature effects on the RNA structure for future investigation. We next investigated whether the dynamics of icSHAPE scores from NAI-N_3_ modifications from the icSHAPE experiment were similar to those obtained with CE-based *in vivo* structure probing. We combined icSHAPE scores (non-depleted RNA library) from the 37°C control group and 26°C treatment group in the trophozoite developmental stage with CE gel results, as shown in Figure 4 (Supplementary Table S6). The region shown here is near the 5′-end (35–135 bp) of 18S A-type rRNA, and icSHAPE scores from the 37°C and 26°C groups for this region are shown in Figure 4A. All nucleotides shown in Figure 4A have scores no less than 1.5, indicating that they are single-stranded at this position (Figure 4A and Supplementary Table S6). We also showed the 37°C control group and 26°C treatment group results on the same region-of-interest nucleotides, which were probed by the conventional gel-based method. The two gels from the 18S A-type rRNA of the 37°C control group and 26°C treatment group from NAI-N_3_ modification were consistent with those for the region from 35–135 bp in the 18S rRNA (Figure 4B, C). Finally, these results indicated that the three genome positions in 18S A-type rRNA (from 5′ to 3′ are 36, 71, and 100) were significantly different between the 37°C control group and the 26°C treatment group (Figure 4D). Meanwhile, a high agreement was observed between the icSHAPE scores and CE gel results. These results further support the possibility that the icSHAPE experiment in this study can help us to obtain genome-wide RNA secondary structure and identify the structural characteristics and regulatory roles of these dynamic mRNAs in cold shock.

**FIGURE 5:**
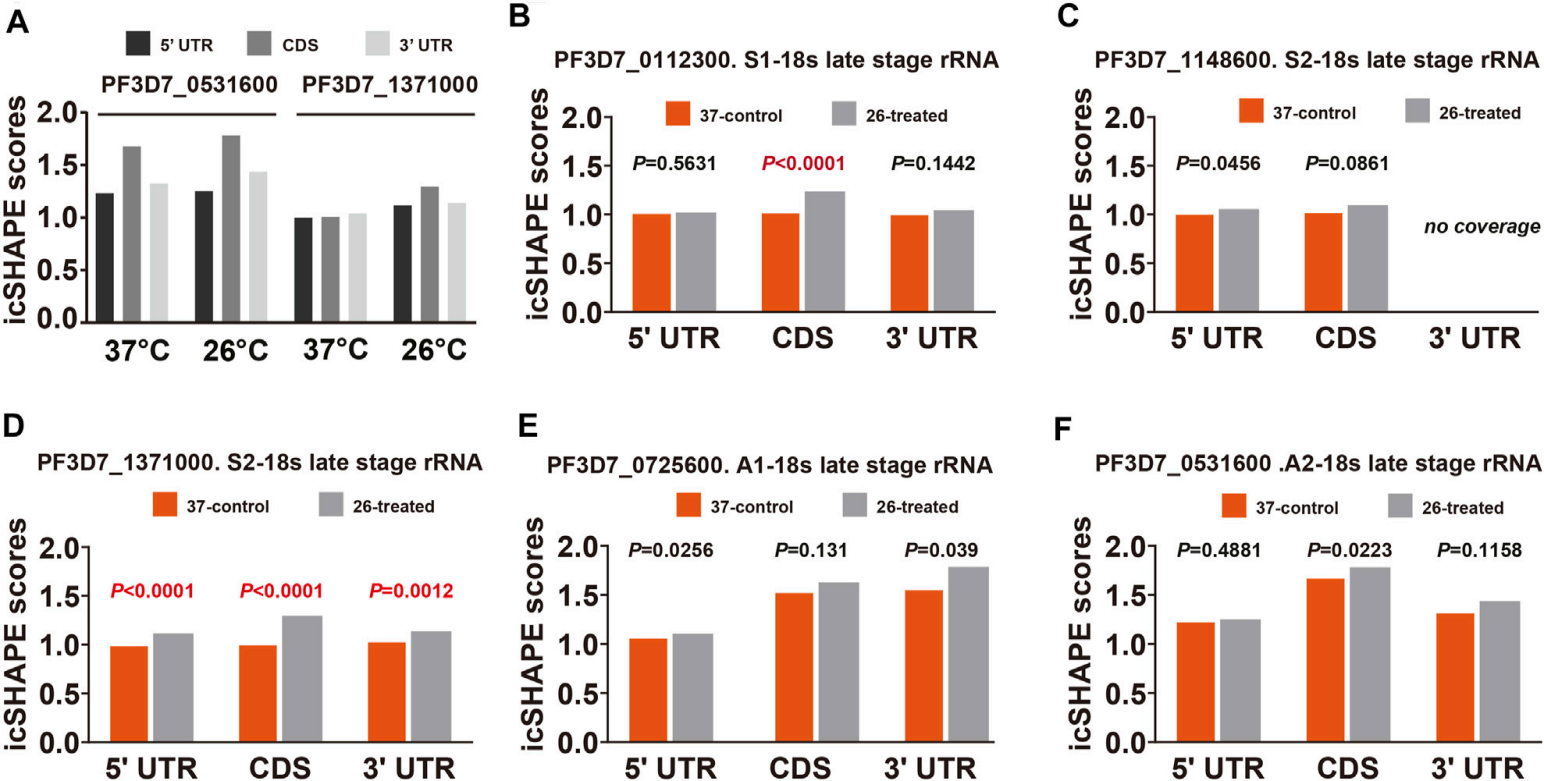
Average icSHAPE on all five subtypes of rRNA’s three regions at two temperatures. **(A)** Comparison of the mean average icSHAPE scores at 37°C and 26°C in the 5′-UTR, CDS, and 3′-UTR regions of 18S A2-type rRNA (PF3D7_0531600) and S2-type RNA (PF3D7_1371000). The mean average icSHAPE scores in the 5′-UTR region, CDS region, and 3′-UTR regions in S1-type **(B)**, S2-type on chromosome 11 **(C)**, S2-type on chromosome 13 **(D)**, A1-type **(E)**, and A2-type **(F)** of 18S ribosomal RNA. The average icSHAPE scores are significantly greater after 26°C treatment for all S2-type 18s rRNA subregions **(D)**, while the average icSHAPE scores from three subregions of A1-type **(E)** and A2-type **(F)**18s rRNA are not significant between 37°C and 26°C. The average icSHAPE scores from three subregions of S1-type 18s rRNA on chromosome 1 **(B)** and S2-type 18s rRNA on chromosome 11 **(C)** are not significant between 37°C and 26°C due to the lower nucleotide coverage (Supplementary Table S5). icSHAPE scores on whole transcripts were cross-normalized between different temperatures (*Materials and Methods*). Specific *p* values for each comparison and coverage of each transcript are provided in Supplementary Table S4.

**FIGURE 6:**
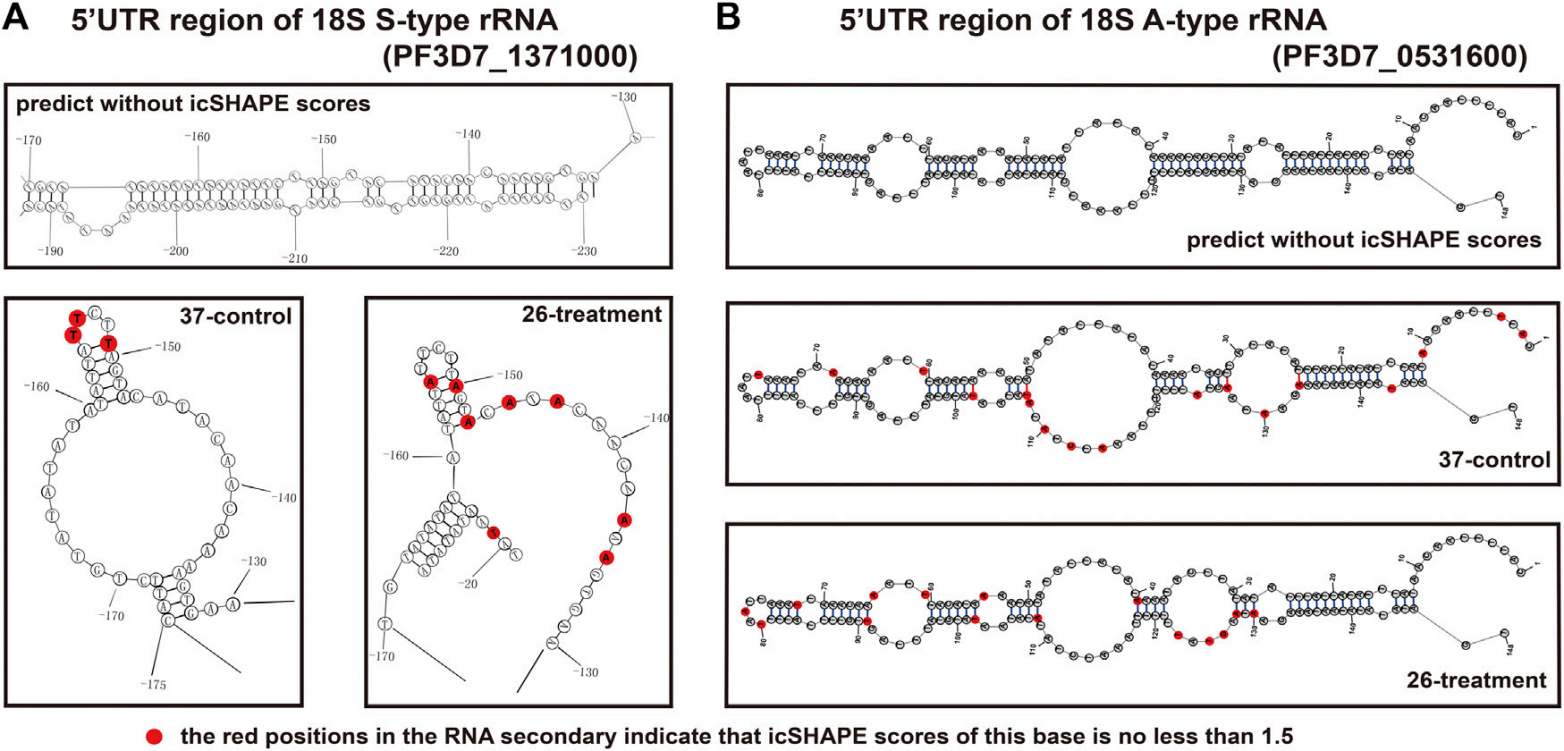
Map of the RNA secondary structure of *P. falciparum* 18S A- and S-type rRNA 5′-UTR. **(A)** Map of the RNA secondary structure of *P. falciparum* 18S S-type rRNA 5′-UTR region without icSHAPE scores, 37°C control group with icSHAPE scores *in vivo*, and 26°C treatment group with icSHAPE scores *in vivo*. **(B)** Map of the RNA secondary structure of *P. falciparum* 18S A-type rRNA 5′-UTR region without icSHAPE scores, 37°C control group with icSHAPE scores *in vivo*, and 26°C treatment group with icSHAPE scores *in vivo*. The red positions in the RNA secondary structure indicate that the icSHAPE scores of this base is no less than 1.5.

To further validate and investigate the mechanism underlying the change in S-type rRNA expression after cold shock, which may be due to the temperature-responsive RNA structures, we next investigated the average *in vivo* icSHAPE score of 18S rRNA on five different chromosomes to show the distribution of icSHAPE profiles (Figure 5). As shown in Figure 5A, the CDS regions of A2-type 18S rRNA (PF3D7_0531600) showed the highest average icSHAPE scores not only in the control group but also in the cold stress group. These positions were treated because they were more accessible to chemical modification and opened their double-strands with a reduced tendency for double-stranded conformation to increase accessibility for subsequent ribosomal protein binding and gene expression. Meanwhile, the icSHAPE scores of the 5′-UTR region in 18S A-type rRNA (PF3D7_0531600) harbor a higher value than S-type RNA (PF3D7_1371000) especially in the 37°C group, which provides a mechanistic basis for the melting of this region and opening of the double-strand for easy ribosomal protein binding and the expression of A-type 18s rRNA. On the other hand, regions that presented nucleotide crowding *in vivo* and/or protein binding were less chemically reactive than the CDS regions. Then, we compared icSHAPE scores from three subregions of the S-type 18S RNA (PF3D7_1371000) in the control and cold stress groups and found that the CDS region is actually predicted to be more accessible to chemical modification and opening of the double-strands for gene expression in the 26°C group than in the control group, which has also been reported for 5′-UTR and 3′-UTR regions; however, these regions are shown to be less accessible and have lower icSHAPE scores than the A2-type 18s rRNA (PF3D7_0531600). These results are consistent with our previous findings that after low-temperature treatment, the expression level of S-type rRNA in the 26°C treatment group was significantly increased compared with that in the 37°C control group, while the A-type rRNA showed little change. These observations also indicate that A-type rRNA is the predominant form in the blood stage development of parasites, and they are consistent with our speculation that transcripts with higher icSHAPE scores also have the most abundant level. After normalization for icSHAPE score differences between the two different temperatures, a significantly different (*p* < 0.01) average icSHAPE score at 26°C compared with 37°C was observed for three subregions in S2-type 18S rRNA (PF3D7_1371000) (Figure 5D), but not in A2-type 18S rRNA (PF3D7_0531600) (Figure 5F). The 5′-UTR showed the most significant (*p* < 0.01) increase in average icSHAPE scores under cold shock compared with the CDS region and 3′-UTR region. Then, we mapped the 5′-UTR region of 18S A-type rRNA (PF3D7_0531600) and S-type RNA (PF3D7_1371000) secondary structures by our icSHAPE scores (Figure 6) and compared the difference in RNA structures between the different temperatures treatments. In Figure 6, the red positions in the RNA secondary structures indicate that the icSHAPE scores of this base are no less than 1.5. These results showed that there was a very large difference in the −170 to −130 region of the 5′-UTR of 18S S-type rRNA (Figure 6A and Supplementary Figure S3), while there were only two small structural changes in the whole 148bp 5′-UTR region of 18S A-type rRNA as shown in Figure 6B and Supplementary Figure S4. These results indicated that the secondary structure changes of 18S rRNA in our experiment determined by the icSHAPE scores were positively correlated with the expression level of A-type and S-type rRNA. Our results imply that the secondary structure of rRNA, especially the 5′-UTR region, has a pivotal role in regulating rRNA expression. However, the regions/bases that are the most important need to be further tested.

### The mRNA Structurome Reveals Numerous RNATs

**FIGURE 7:**
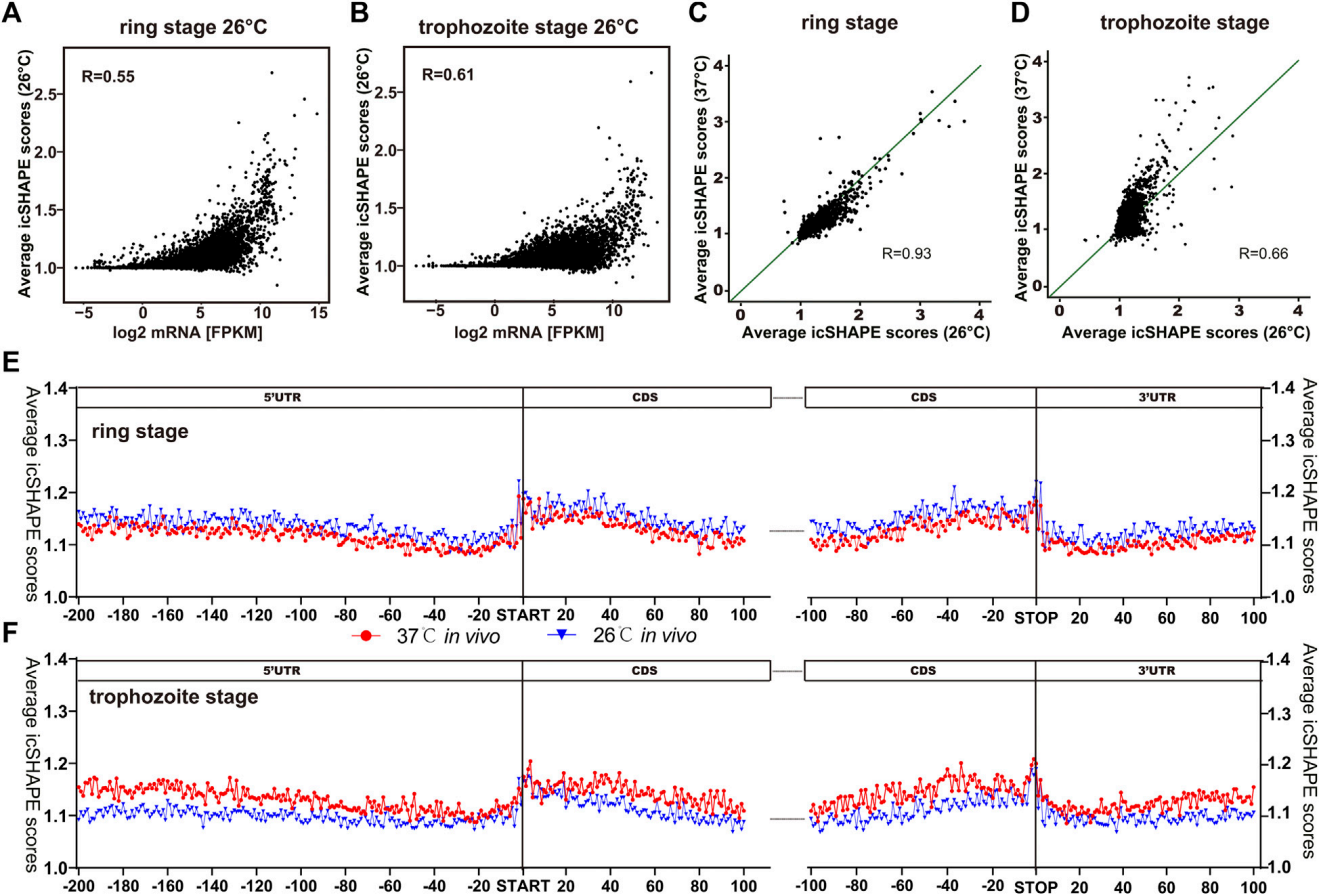
Functional analysis of the mean icSHAPE scores across the entire transcripts. **(A, B)** Dependence of the average icSHAPE scores *in vivo* on the mRNA expression level of the entire transcripts from the trophozoite and ring stages at 26°C. **(C)** The average icSHPE scores from the ring stage are nearly the same between 37°C and 26°C, with no significance. **(D)** The average icSHPE scores from the trophozoite stage are significantly greater at 37°C than 26°C. Pearson correlation coefficient (*R*) is showed in each panel. **(E, F)** The average icSHAPE score collected from the CDS regions and the UTR regions of different transcriptome *in vivo* among the ring stage and trophozoite stage after low-temperature stimuli, respectively. There are some regions that have significantly different icSHAPE scores between the 37°C control group and the 26°C treatment group, such as around −55 nt at 5′-UTR region, 25 nt at CDS region, and 10 nt at 3′-UTR region.

The RNA secondary structure can regulate the mRNA translation efficiency of certain genes in a widespread fashion (Mustoe et al., 2018). In our study, we investigated the relationship between different gene expression patterns in *P. falciparum* development by changing physiologically relevant temperatures and RNA secondary structures. We compared the average icSHAPE score from the CDS region of individual transcripts, and the results displayed a correlation that transcripts with higher icSHAPE scores also have most abundant level (Figure 7A, B). After normalization for NAI-N_3_ reactivity differences between two different temperatures and the two developmental stages, a global trend of significantly reduced average icSHAPE scores for entire transcripts *in vivo* at 26°C compared with 37°C was observed (Figure 7C, D) in the trophozoite stage, but not in the ring stage. Reproducibility was confirmed by comparisons between biological replicates. This result indicates that low temperatures may suppress the growth and development of parasites, with the maximal effect on the trophozoite stage of parasites. Additionally, the same pattern was observed when we compared the average icSHAPE scores for entire transcripts at 37°C *in vitro* with 26°C *in vitro* (Supplementary Figure S5).

We also compared the average icSHAPE scores of CDS regions and the UTR regions of each different transcriptome *in vivo* among the ring stage and trophozoite stage after low-temperature stimuli in *P. falciparum* (Figure 7E, F). By comparing the icSHAPE score at the same stage but at different temperatures, we found that there were some regions that had significantly different icSHAPE scores between the 37°C control group and 26°C treatment group, such as that at approximately −55 nt at 5′-UTR region, 25 nt at CDS region, and 10 nt at 3′-UTR region. Additionally, a sharp rise in average icSHAPE scores near the stop and start codons indicated that these two regions with decreased structure may have a significant role in the efficiency of translation. Our results imply that these features may favor changes in RNA structure in response to low-temperature stress; however, the mechanism remains to be tested in the future.

**FIGURE 8:**
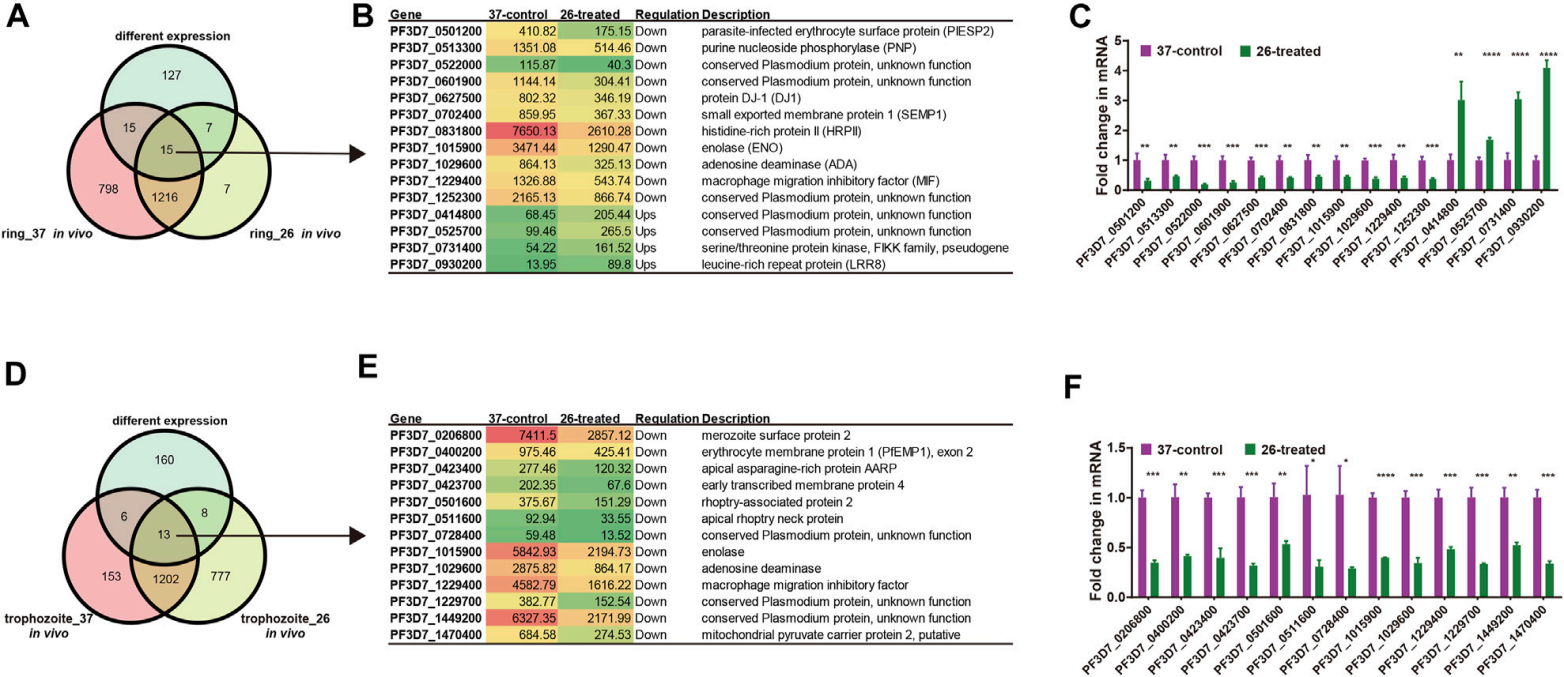
List of genes included in our structure analysis. **(A, D)** Overlap of *Plasmodium* mRNAs in the ring stage **(A)** and trophozoite stage **(D)** with adequate structure probing *in vivo* coverage between 26°C and 37°C temperature and with different expressions between two temperatures. **(B, E)** List of all genes with different expressions after cold response in the ring stage **(B)**/trophozoite stage **(E)** and with average nucleotide coverage above 2. Columns show, for each gene, the gene name, the FPKM value, the regulation, and gene description. Red color indicates high expression level and green color indicates low expression level. **(C, F)** qPCR analyses of transcriptional regulation [fold changes at 3 h and 26°C temperature stimulation to 37°C at 45 h (ring stage)/70 h (trophozoite stage)] after invasion. A comparison of the gene expression in different treated groups in the ring developmental stage between RNA-seq and qPCR. Fold changes in the expression of different genes in the 26°C treatment group were detected by qRT-PCR. The name of each gene is shown at the bottom. The qPCR results were highly consistent with the RNA-seq analysis. The data are representative of three independent experiments. Two-sided *t*-test, *p < 0.05; **p < 0.01; ***p < 0.001; ****p < 0.0001; ns, no significant (*t* test; SDs from three replicates).

We next investigated the change in the RNA secondary structure of those genes, which have a reverse transcription termination coverage rate of transcripts no less than 2 in both two developmental stages and DEGs from the ring stage and trophozoite stage at different culture temperatures for 3 h. When the changed transcripts in the ring stage development were compared, those transcripts included 15 genes, in which 4 genes were upregulated and 11 genes were downregulated. A comparison of transcripts in the trophozoite stage development identified 13 genes located on different chromosomes of the parasites, and all of the 13 genes were downregulated (Supplementary Table S7). To experimentally verify the accuracy of RNA-seq in the pattern of changes induced by low-temperature stimuli, the fold changes in the expression of different genes in the 26°C treatment group were detected by qPCR. The list of genes that needed to be detected at the mRNA level by qRT-PCR is shown in Figure 8, and the DNA primers used in qRT-PCR assay are listed in Supplementary Table S1. The qPCR results, which are representative from three independent experiments, were highly consistent with those of the RNA-seq analysis.

RNA thermometers, in which changes in temperature lead to alterations in the 5′-UTR region of individual RNAs, usually modulate translation efficiency in bacteria (Kortmann and Narberhaus, 2012). For our analysis, we compared and identified whether the differential expression of transcripts occurred due to changes in the 5′-UTR structure of individual RNAs. Among all 15 differentially expressed RNAs in the ring stage and 13 differentially expressed RNAs in the trophozoite stage between different temperatures, our global map of the 5′-UTR of the RNA structure results showed that it has different icSHAPE scores (Table 1) and different predicted RNA secondary structures with icSHAPE scores (Supplementary Table S8 and Supplementary Figure S6). These results showed that the 5′-UTR region of the majority of mRNAs (except PF3D7_0513300 in the ring stage and PF3D7_1029600 and PF3D7_1449200 in the trophozoite stage) folded significantly differently *in vivo* at the two different temperatures and was also different from the predicted structure. As an additional confirmation of the structural information, we also predicted the *in vitro* RNA structures from the same samples and culture conditions using icSHAPE scores (Supplementary Figure S6). The global 5′-UTR of secondary structure probing maps at different temperature profiles can help us to reveal a large number of RNAT candidates in malaria parasites. Fourteen candidate genes, which were located on 14 different chromosomes and did not show differential expression after cold shock in the two stages, were selected, and the RNA secondary structures of the 5′-UTR region of the individual RNAs were drawn (Supplementary Table S8 and Supplementary Figure S6). The majority of those 14 genes have the same RNA structure.

**TABLE 1:** List of the RNAT candidates according to cold stress in *Plasmodium* [columns show, for each gene, the name, annotation developmental stage, regulation, *t*-test, and average of icSHAPE scores of 5′-UTR (−1 to −200)].

Gene	Annotation	Developmental stage	Regulation	icSHAPE scores^a^	icSHAPE scores^a^	icSHAPE scores^a^	icSHAPE scores^a^	t-test^b^	t-test^b^	t-test^b^	t-test^b^	Prediction of thermal control^c^
				37 *in vivo*	37 *in vitro*	26 *in vivo*	26 *in vitro*	37 *in vivo* to 26 *in vivo*	37 *in vivo* to 37 *in vitro*	37 *in vitro* to 26 *in vitro*	26 *in vivo* to 26 *in vitro*	
PF3D7_0501200	Parasite-infected erythrocyte surface protein (PIESP2)	Ring stage	Down	1.11318415759915	1.16517762208649	1.12208760171332	1.15361949399859	0.8957	0.358	0.8364	0.5975	N
PF3D7_0513300	Purine nucleoside phosphorylase (PNP)	Ring stage	Down	1.05551215344291	1.10014243370182	1.20362784225472	1.16513278752761	0.1141	0.5199	0.307	0.6724	N
PF3D7_0522000	Conserved *Plasmodium* protein, unknown function	Ring stage	Down	1.54007122545246	1.08061806724822	1.3031907845106	1.15059888058864	0.0267	<0.0001	0.246	0.0334	Y
PF3D7_0601900	Conserved *Plasmodium* protein, unknown function	Ring stage	Down	1.18189769379726	1.13401884078101	1.1169749519831	1.02337140052	0.3828	0.3364	0.0268	0.1116	N
PF3D7_0627500	Protein DJ-1 (DJ1)	Ring stage	Down	1.41231124107859	1.04648820423592	1.38385240343451	1.12358480847633	0.782	<0.0001	0.2099	0.0025	N
PF3D7_0702400	Small exported membrane protein 1 (SEMP1)	Ring stage	Down	1.12341976835768	1.21028850831799	1.21906053606498	1.13520385700056	0.2147	0.0902	0.1243	0.1761	N
PF3D7_0831800	Histidine-rich protein II (HRPII)	Ring stage	Down	0.999505440685413	1.15449493777787	1.03724600135267	1.1377811954296	0.5155	0.0012	0.7201	0.0572	N
PF3D7_1015900	Enolase (ENO)	Ring stage	Down	0.798003529197416	1.14707470399586	1.33361069766907	1.27768248141225	<0.0001	<0.0001	0.054	0.4921	Y
PF3D7_1029600	Adenosine deaminase (ADA)	Ring stage	Down	0.987384463053602	0.962874313840553	1.04707352441963	1.0635068020404	0.2117	0.5178	0.0239	0.8299	N
PF3D7_1229400	Macrophage migration inhibitory factor (MIF)	Ring stage	Down	1.50638846227964	1.32304906980165	1.5747346503315	1.23955931811926	0.5718	0.0124	0.2836	0.0005	N
PF3D7_1252300	Conserved *Plasmodium* protein, unknown function	Ring stage	Down	1.04763368375218	1.22947078863875	1.07929809559152	1.08654676711955	0.6523	0.0035	0.0325	0.8923	N
PF3D7_0414800	Conserved *Plasmodium* protein, unknown function	Ring stage	Ups	1.07811563136032	1.05522040160658	1.07334134174054	1.13533883400995	0.9414	0.5128	0.0504	0.2192	N
PF3D7_0525700	Conserved *Plasmodium* protein, unknown function	Ring stage	Ups	1.10936792958133	1.04417044238781	1.24245904664744	1.03760148519355	0.0701	0.1182	0.852	0.0037	N
PF3D7_0731400	Serine/threonine protein kinase, FIKK family, pseudogene (FIKK7.2)	Ring stage	Ups	1.06995986412861	1.01936965009826	1.54563474728354	1.27575465881603	<0.0001	0.2264	<0.0001	0.002	Y
PF3D7_0930200	Leucine-rich repeat protein (LRR8)	Ring stage	Ups	1.10556027381952	1.13699701579546	1.18388186967829	1.25151415106983	0.4487	0.4922	0.1461	0.4138	N
PF3D7_0206800	Merozoite surface protein 2 (MSP2)	Trophozoite stage	Down	2.21844005554365	2.16545058333929	2.11998529445452	3.1445161811697	0.5741	0.8875	0.0677	0.0043	N
PF3D7_0400200	Erythrocyte membrane protein 1 (PfEMP1), exon 2, pseudogene	Trophozoite stage	Down	1.27476907148766	0.985586017225317	1.19968156791575	1.36388251163218	0.4553	0.0166	0.0554	0.348	N
PF3D7_0423400	Asparagine-rich protein (AARP)	Trophozoite stage	Down	1.67944930869978	1.02129980519896	1.19180846523002	1.60243963083084	0.0001	0.0006	0.0084	0.0048	Y
PF3D7_0423700	Early transcribed membrane protein 4 (ETRAMP4)	Trophozoite stage	Down	1.20406678881567	1.22887569496438	0.998513656655461	2.05902494510159	0.0034	0.8218	0.0003	<0.0001	Y
PF3D7_0501600	Rhoptry-associated protein 2 (RAP2)	Trophozoite stage	Down	1.09533960925014	1.62355805179727	1.27728255309109	1.64814477369071	0.0246	0.0214	0.932	0.0107	Y
PF3D7_0511600	Apical rhoptry neck protein (ARNP)	Trophozoite stage	Down	1.24165674459331	1.23586870613038	1.0879861728563	1.28328242535274	0.0221	0.9684	0.7997	0.1673	Y
PF3D7_0728400	SDH5 domain-containing protein, putative	Trophozoite stage	Down	1.19190147029617	1.13413906948208	1.05619996510389	0.914546782322872	0.1202	0.6884	0.1559	0.0461	N
PF3D7_1015900	Enolase (ENO)	Trophozoite stage	Down	1.71355363932401	1.0716817274604	1.00042445166024	1.72097707481264	<0.0001	0.0025	0.0263	0.0014	Y
PF3D7_1029600	Adenosine deaminase (ADA)	Trophozoite stage	Down	1.41153901669993	1.03159610002481	1.01977810981006	1.06868900952739	<0.0001	0.0007	0.7843	0.6188	Y
PF3D7_1229400	Macrophage migration inhibitory factor (MIF)	Trophozoite stage	Down	2.33310582323676	0.914583765477988	1.19120400565315	1.39946364811451	<0.0001	<0.0001	0.0609	0.3264	Y
PF3D7_1229700	Mitochondrial respiratory chain complex II subunit, putative	Trophozoite stage	Down	1.47608474032324	1.0313131039331	1.1038959925848	0.985230549512947	0.0003	0.0019	0.76	0.2107	Y
PF3D7_1449200	Conserved *Plasmodium* protein, unknown function	Trophozoite stage	Down	1.43496065780095	1.40867302270062	1.30183163153328	1.66476386061194	0.1127	0.8908	0.2791	0.0352	N
PF3D7_1470400	mitochondrial pyruvate carrier protein 2, putative (MPC2)	Trophozoite stage	Down	1.30452521077698	1.53527932743505	1.23082069692473	1.30187277020204	0.3716	0.257	0.4504	0.7687	N

a In vivo and in vitro average icSHAPE scores of the 5′-UTR region (−200 to −1 from the start codon) measured at 26°C and 37°C.

b Two-tailed paired Student’s t-test applied in icSHAPE scores of the 5′-UTR region (−200 to −1 from the start codon) from 37°C *in vivo* to 26°C in vivo, 37°C in vivo to 37°C in vitro, 37°C in vitro to 26°C in vitro, and 26°C in vivo to 26°C in vitro, respectively.

c Prediction of thermal control. Y, thermal control (37°C in vivo to 26°C in vivo, P value <0.05); N, no thermal control (37°C in vivo to 26°C in vivo, P value ≥0.05)

Because in RNA structure some nucleotides that were crowded *in vivo* and/or protein binding can affect the prediction of RNA secondary structure even with icSHAPE scores, to more accurate identify temperature-responsive RNA structures of the above 25 genes (Supplementary Table S8, three genes are not only in the ring stage but also in the trophozoite stage) in *Plasmodium*, we compared *in vivo* and *in vitro* average icSHAPE scores of the 5′-UTR region (−200 to −1 from the start codon) measured at 26°C and 37°C (Table 1). To further evaluate the relationship between icSHAPE scores and RNA structure change, two-tailed paired Student’s *t*-test was applied in icSHAPE scores of the 5′-UTR region (−200 to −1 from the start codon) from 37°C *in vivo* to 26°C *in vivo*, 37°C *in vivo* to 37°C *in vitro*, 37°C *in vitro* to 26°C *in vitro*, and 26°C *in vivo* to 26°C *in vitro*, respectively. Because crowded nucleotides or protein binding can affect icSHAPE scores, only a *p* value <0.05 of two-tailed paired Student’s *t*-test was considered a functional RNAT. Finally, these results suggested that at least 11 genes are the most potential RNAT in *Plasmodium* after cold shock. In future research, it would be interesting to confirm the mechanisms of the RNATs found by reporter gene fusions or single-nucleotide changes in the RNAT regions.

Overall, our study adds to the accumulating evidence that suggests that the special RNA structure, especially 5′-UTR regions, has dramatic effects on the complex biological program of *P. falciparum*. Additional studies are needed to confirm the RNATs found in our study by single-nucleotide changes in the RNAT regions.

## Discussion

The development and transmittance of malaria parasite is based on complex environmental changes, and the parasite must face dynamic changes during their developmental cycles (Fang, 2004). Therefore, a quick response to these alterations need to be developed. Among these changes, temperature is the most determining factor for a mammalian pathogen entering its host (Righetti and Narberhaus, 2014). Temperature control is extremely important for malaria development, and fluctuations in ambient temperature can affect the development of *P. falciparum* in the host and mosquito (Fang and McCutchan, 2002; Qi et al., 2015). Thermoregulation of *P. falciparum* has been extensively studied for its regulation of rRNA, gametogenesis, drug response, and parasite sequestration in cerebral malaria (Singhaboot et al., 2019). Additionally, cold shock can affect parasite growth and alter the drug response of parasites and has been proposed for the therapy of CM. This study implies that low temperature treated-parasites display variations in the expression of several genes that have important functions in malaria growth and that parasites can respond to environmental temperature by reducing their transmission and replicative fitness. Some studies have illustrated the post-translation control mechanism, including the genetic and epigenetic mechanisms. However, the exact mechanism remains unknown.

The RNA secondary structure has recently been demonstrated as an essential factor that contributes to gene expression (Bevilacqua et al., 2016), such as transcription (Schmitz et al., 2010), RNA maturation (Buratti and Baralle, 2004), and translation initiation (Kutchko et al., 2015). RNATs are usually located in the 5′-UTR region and form a base-paired structure in bacteria (Kortmann and Narberhaus, 2012; Su et al., 2018). RNAT modulation plays a role in the differential regulation of individual genes in the infection and host adaptation processes of many organisms, such as *Yersinia pseudotuberculosis* (Righetti et al., 2016), *Neisseria meningitidis* (Loh et al., 2013), *Pseudomonas aeruginosa* (Grosso-Becerra et al., 2014), and *Vibrio cholerae* (Weber et al., 2014). Here, we performed a genome-wide RNA secondary structure study *in vitro* and *in vivo* during the parasites’ trophozoite and ring stages in the intra-erythrocytic developmental cycle in response to temperature changes. Our genome-wide icSHAPE recapitulates the results obtained by CE methods and can provide a genome-wide view of the RNA structure landscape of *P. falciparum* after cold shock.

This study identified some functional RNA structural elements, demonstrated that a dynamic RNA structurome responds to two different temperatures, and identified novel RNA thermometers. Our study adds to the accumulating evidence that febrile temperatures can suppress the growth and development of parasites with a maximal effect on trophozoites (Tinto-Font et al., 2021). We identified a global trend of significantly reduced average icSHAPE scores for entire transcripts at 26°C compared with those at 37°C in the trophozoite stage based on the icSHAPE *in vivo*, as well as icSHAPE *in vitro*. One possibility is that cold shock response mRNAs are more structured (low icSHAPE scores) in the trophozoite stage and may assume multiple conformations that have regulatory purposes.

A previous study in *P. falciparum* reported that the most abundant transcripts exhibited higher icSHAPE scores in human host body temperature (Qi et al., 2021). We also observed a sharp rise in average icSHAPE scores near the stop and start codons, which is associated with the efficiency of mRNA transcription. Consistent with this conclusion, we observed that the development of *P. falciparum* is connected to the RNA secondary structure, even in *P. falciparum* after cold shock. Cold-induced icSHAPE score changes were strongly correlated with cold-induced changes in transcript abundance. A directed analysis of the average icSHAPE score collected from the CDS regions and the UTR regions revealed that certain regions had significantly different icSHAPE scores after cold stress. Thus, at present, our experimental and computational conclusions suggest that these positions may provide candidate sites for the functional conformation of mRNA in response to low-temperature stress; however, their interaction with regulatory proteins needs to be tested further.

Based on a parallel analysis of RNA structures in our experimental and computational analysis, we mapped the 5′-UTR structures of 44 genes at two different temperatures. An interesting observation from our global map of the 5′-UTR of the RNA structure results is that the majority of mRNAs folded significantly differently *in vivo* at two different temperatures. These candidate 5′-UTRs of secondary structure probing maps at different temperature profiles can help reveal a large number of RNAT candidates in malaria parasites. These results imply that these features might favor changes in RNA structure in response to low-temperature stress conditions in parasites; however, the mechanism must be tested in the future. A possible RNAT was determined in *P. falciparum*, and our study provides the first structurome for post-transcriptome regulation in response to the temperature changes. Identifying cold response molecules will provide new clues for further understanding the virulence and development of *P. falciparum*.

## Data Availability

The datasets presented in this study can be found in online repositories. All the RNA-seq data and icSHAPE-seq data sets discussed in this paper have been deposited in National Center for Biotechnology Information (NCBI) Sequence Read Archive under the BioProject ID PRJNA625172.
