# Supplementary material for: RNA Secondary Structurome Revealed Distinct Thermoregulation in Plasmodium falciparum
Source: Front Cell Dev Biol. 2022 Jan 4;9:766532. doi: 10.3389/fcell.2021.766532 (PMC8763798; doi:10.3389/fcell.2021.766532)

PF3D7_0501200

Supplementary Figure 6

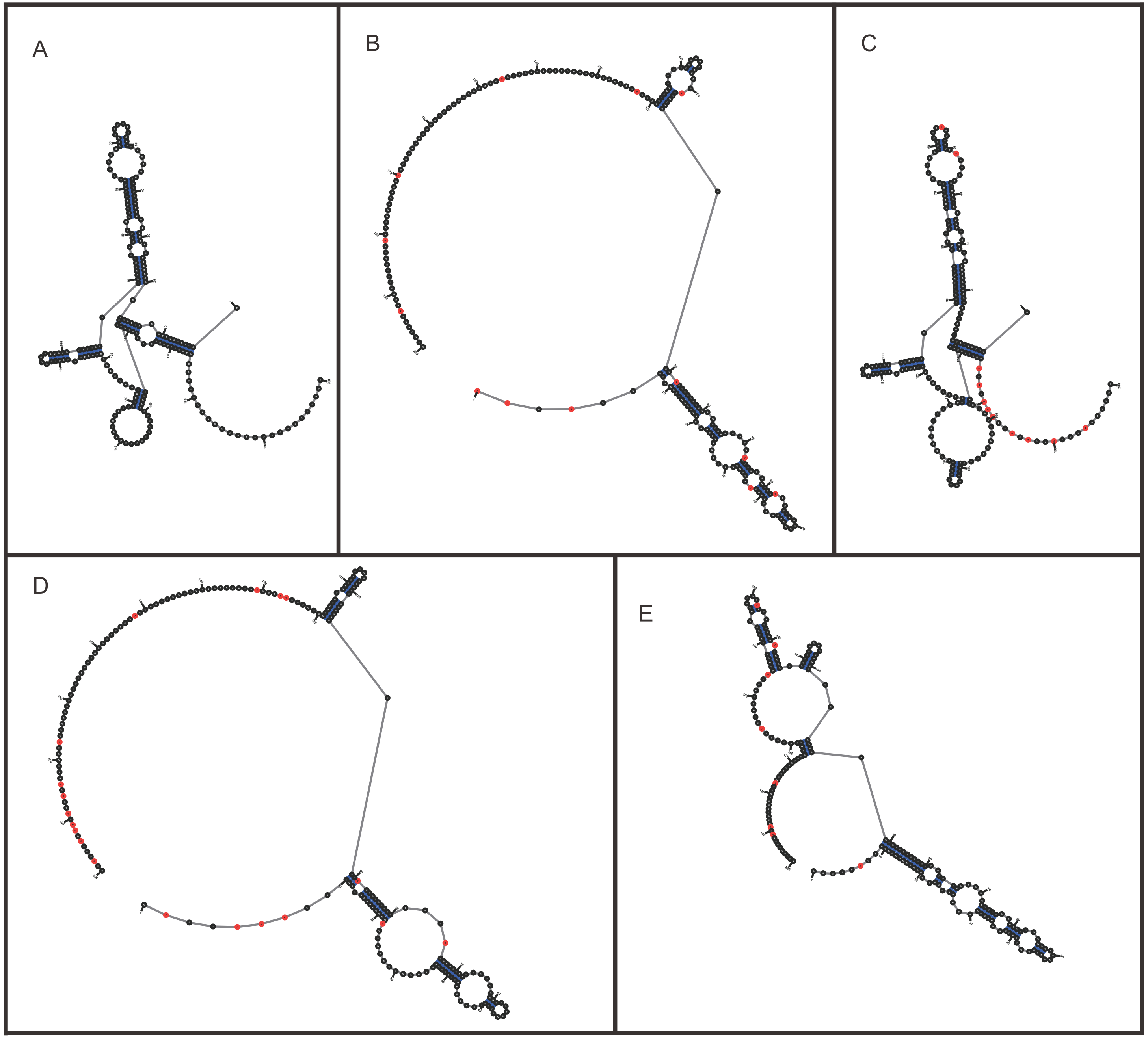

more interior loop after cold stress

● the red positions in the RNA secondary indicate that icSHAPE scores of this base is no less than 1.5

PF3D7_0513300

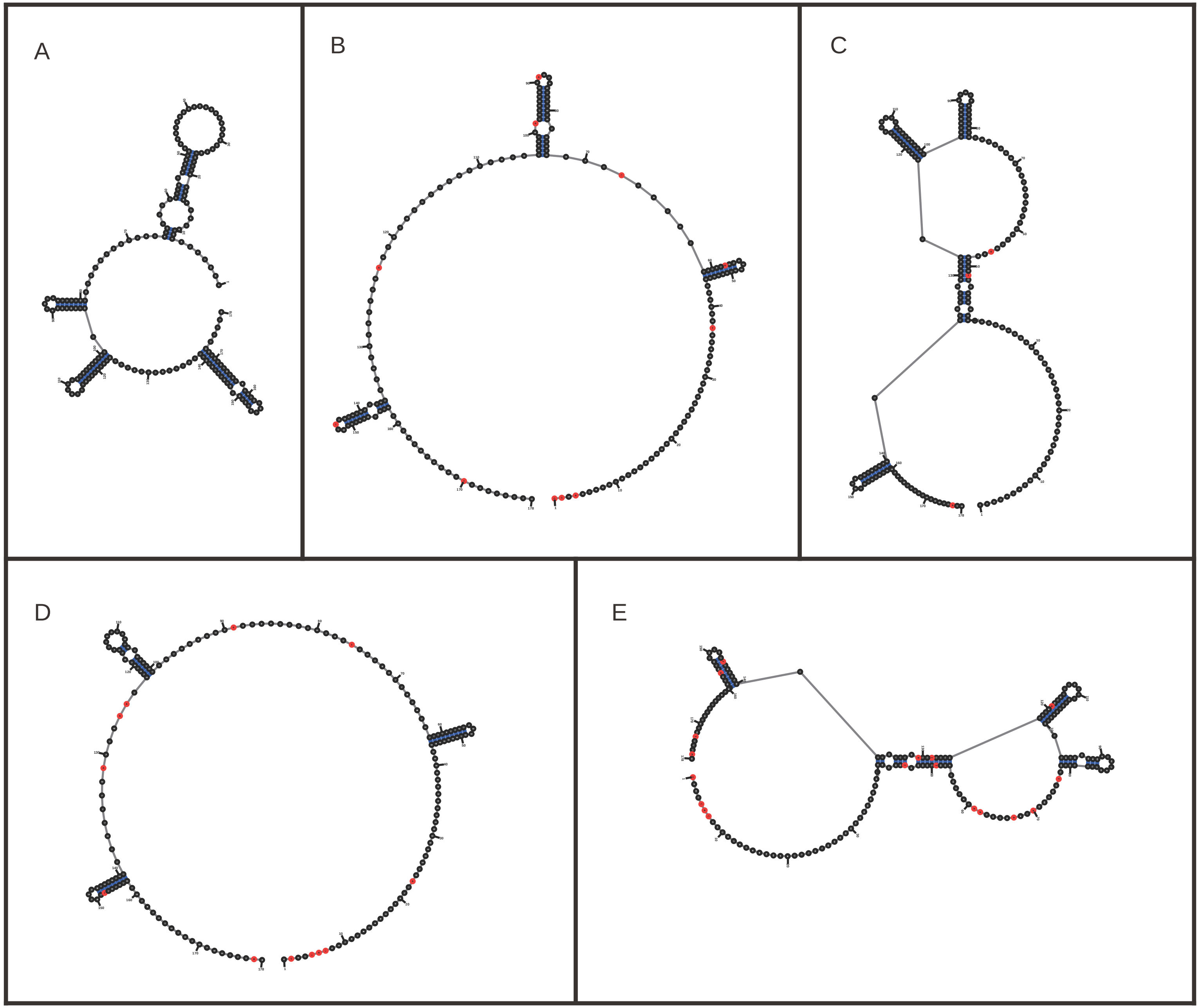

similar structure

PF3D7_0522000

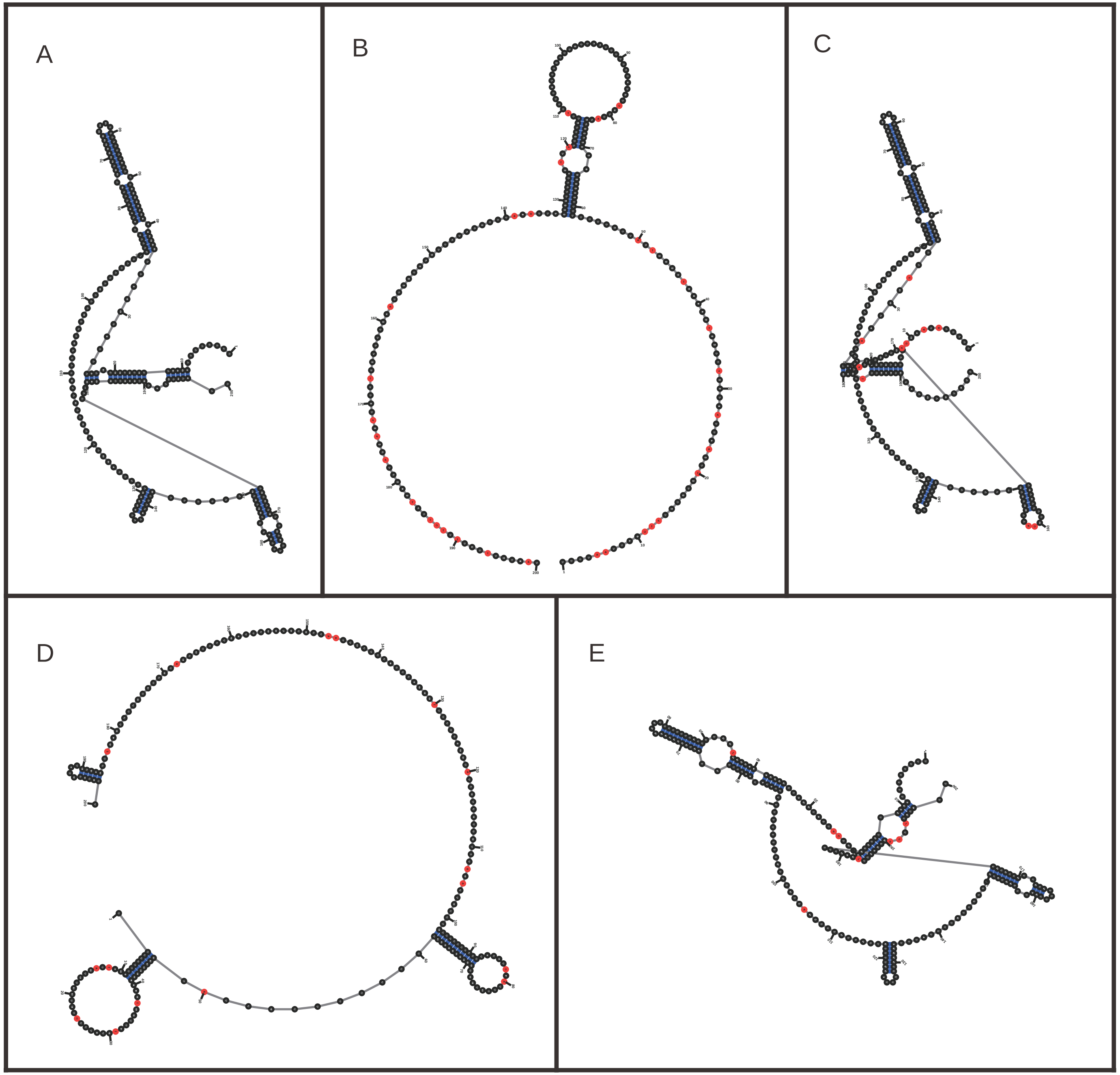

more hairpin loop after cold response

PF3D7_0601900

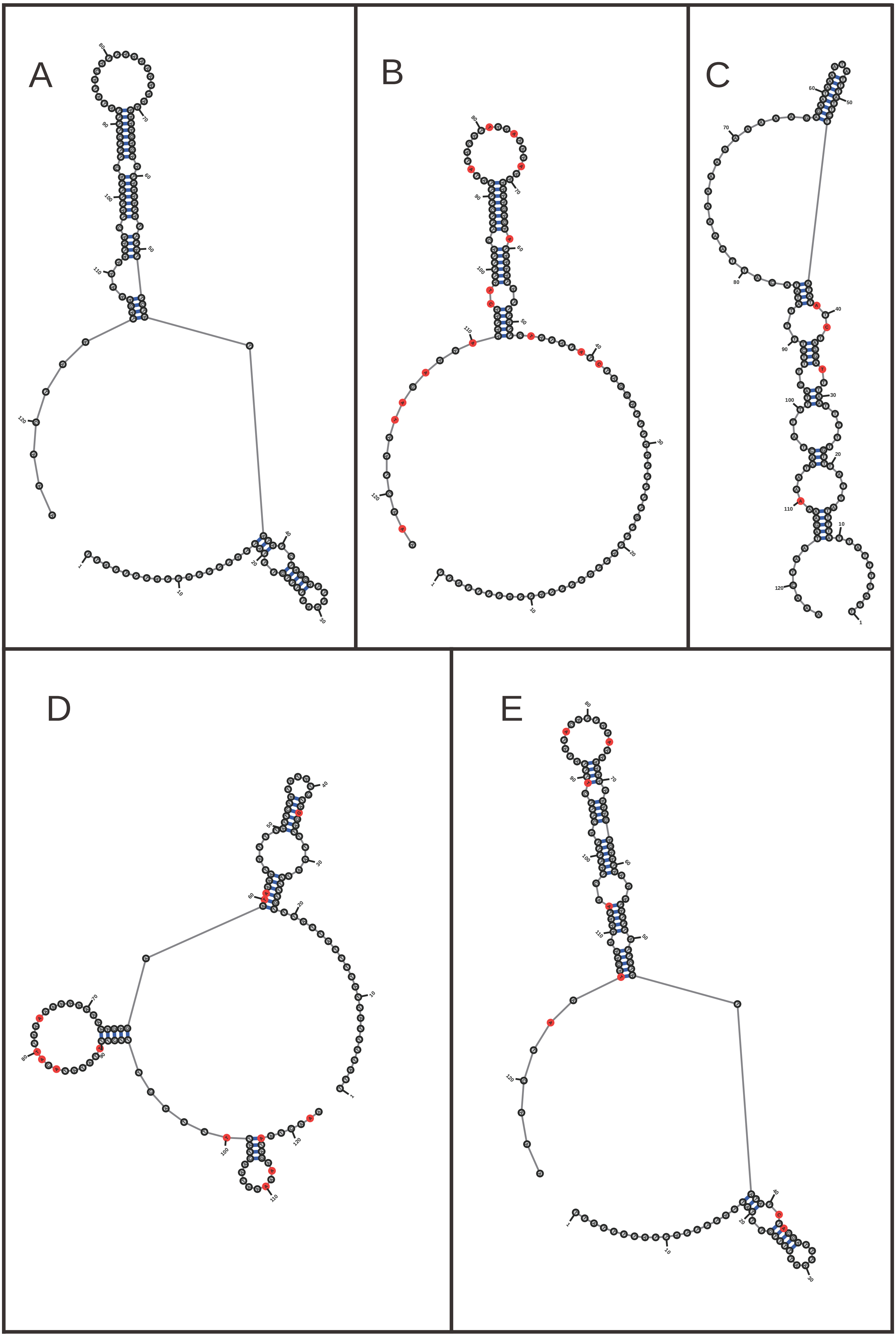

more hairpin loop after cold response

PF3D7_0627500

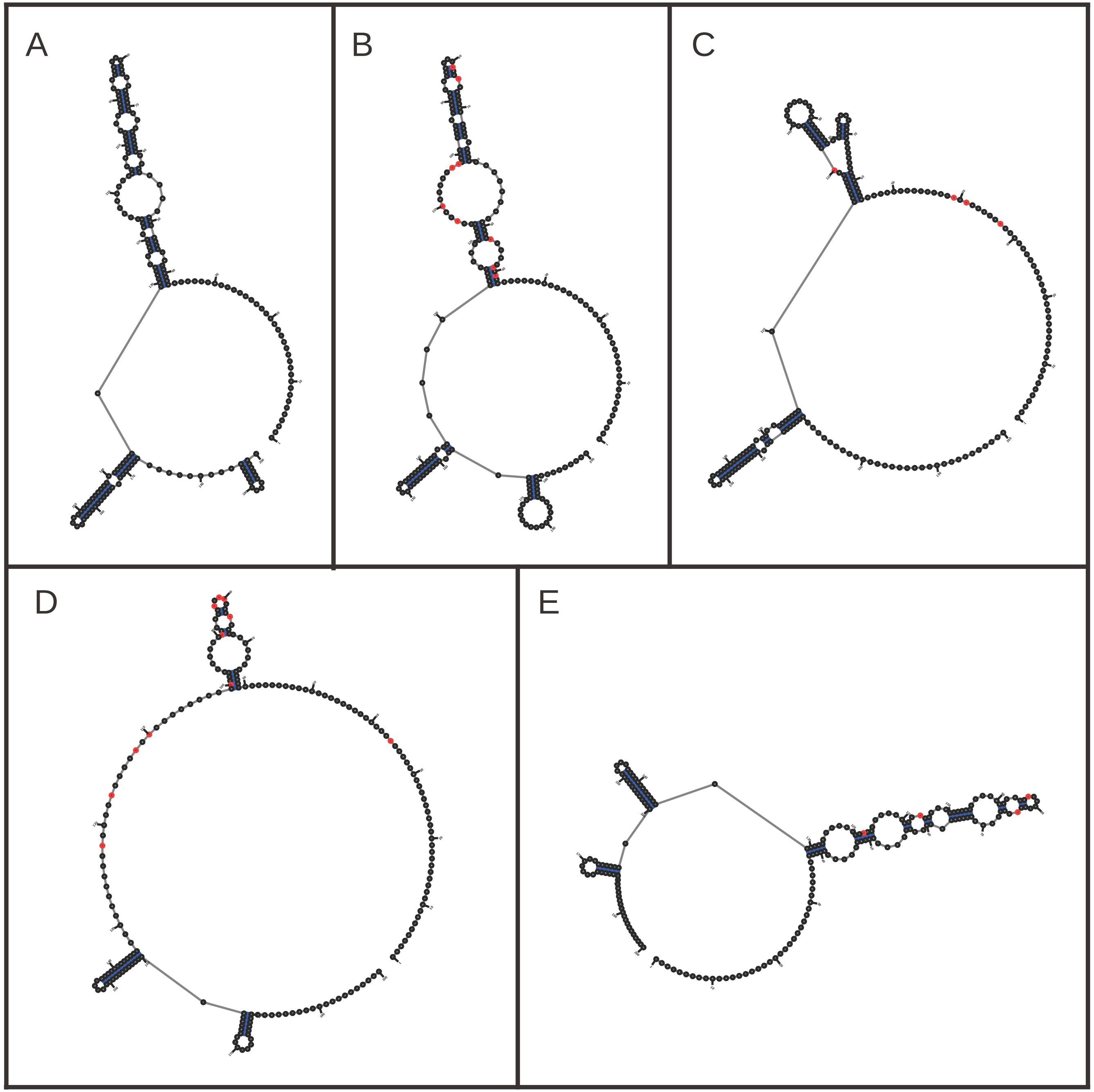

more interior loop than cold stress

PF3D7_0702400

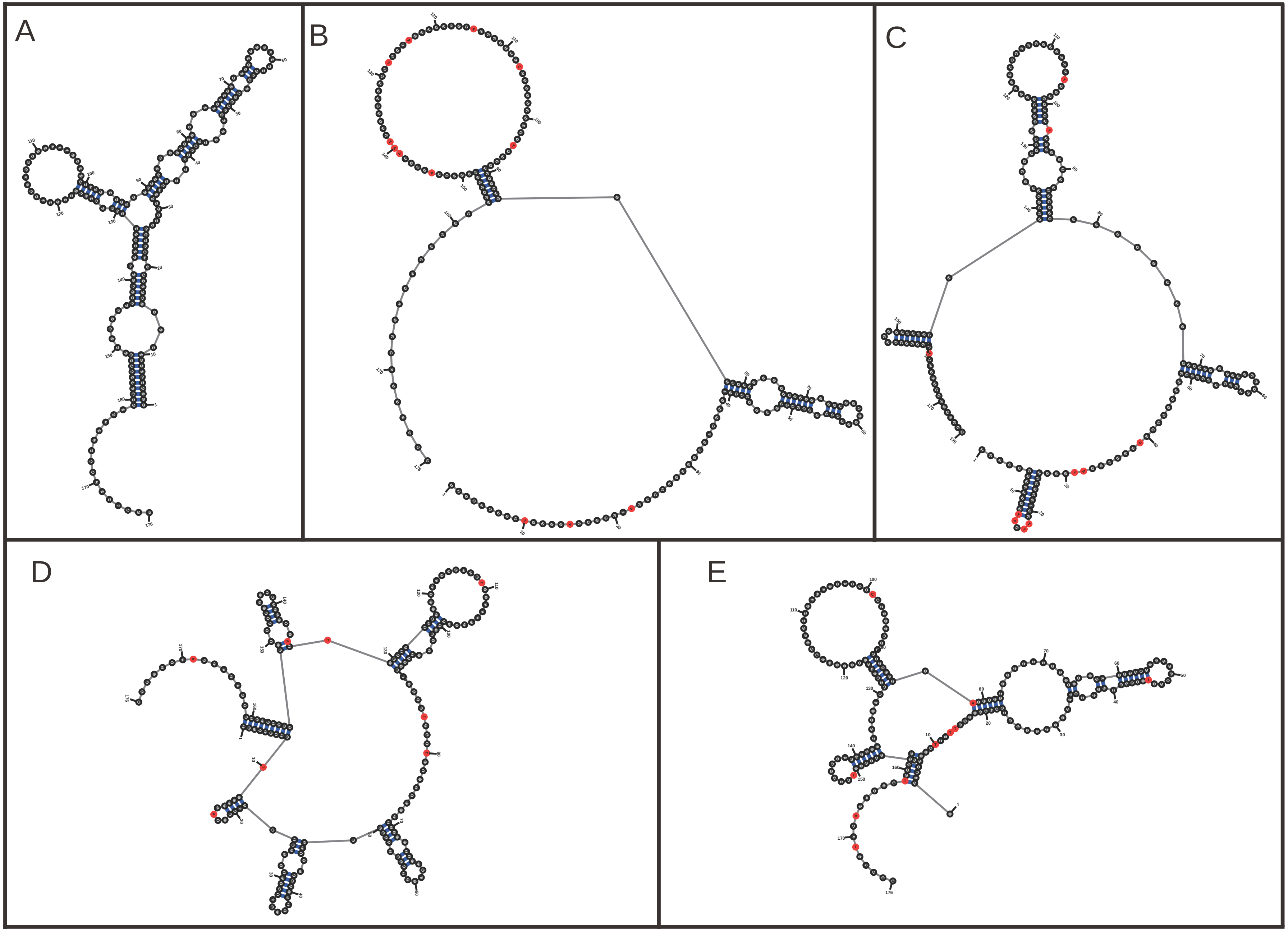

more hairpin loop and interior loop after cold response

PF3D7_0831800

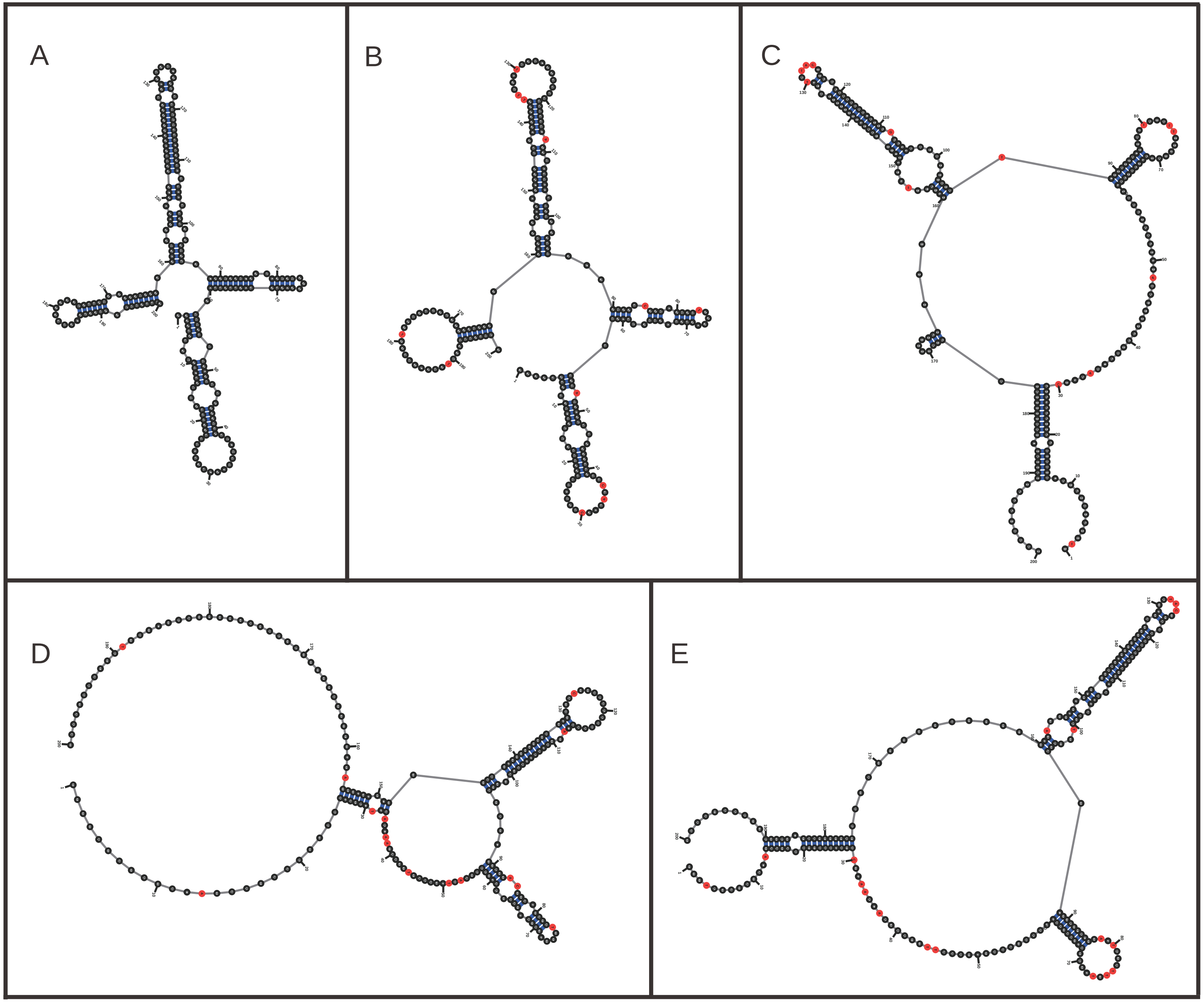

totally different

PF3D7_1015900

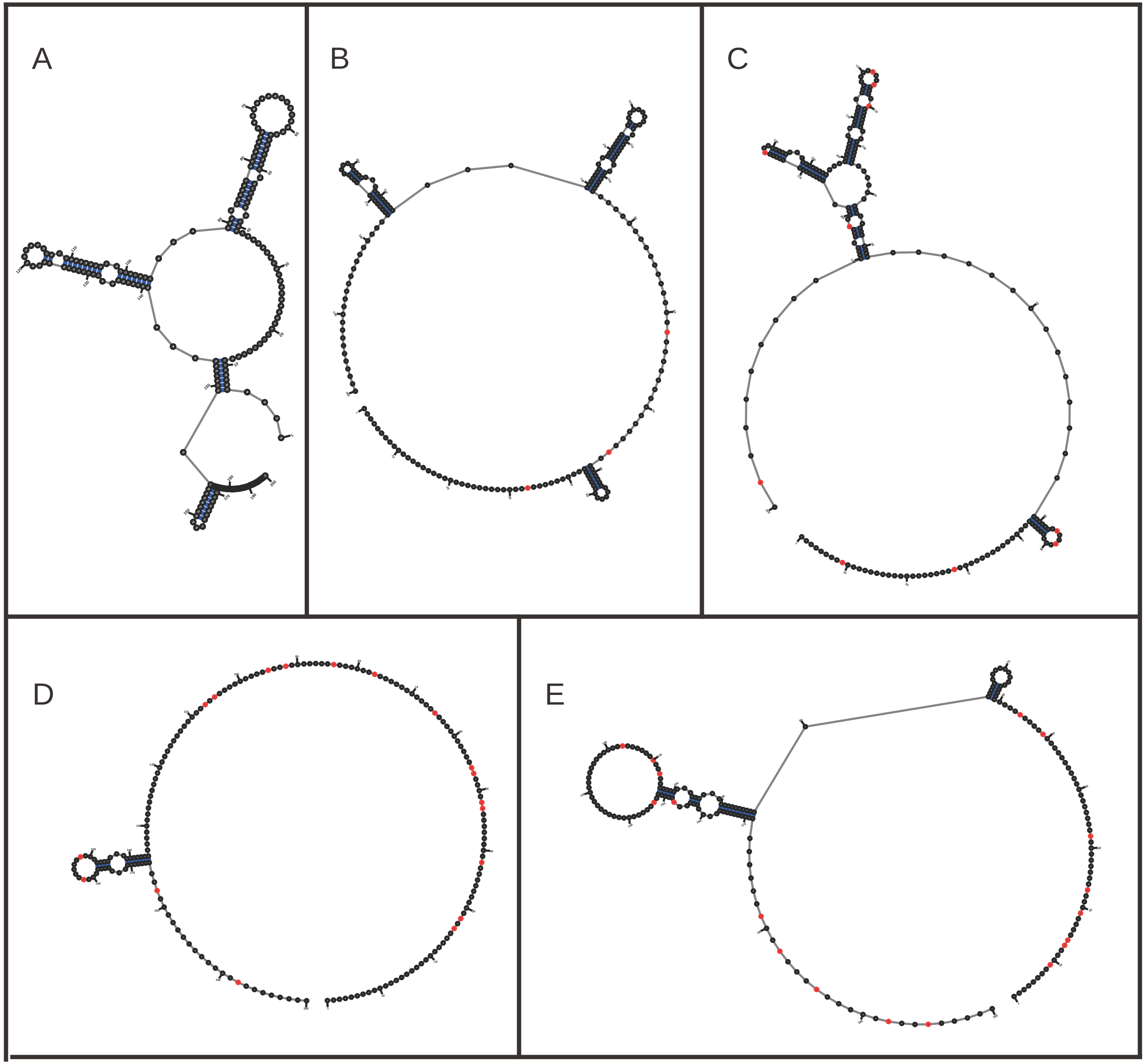

more hairpin loop and interior loop than cold stress

PF3D7_1029600

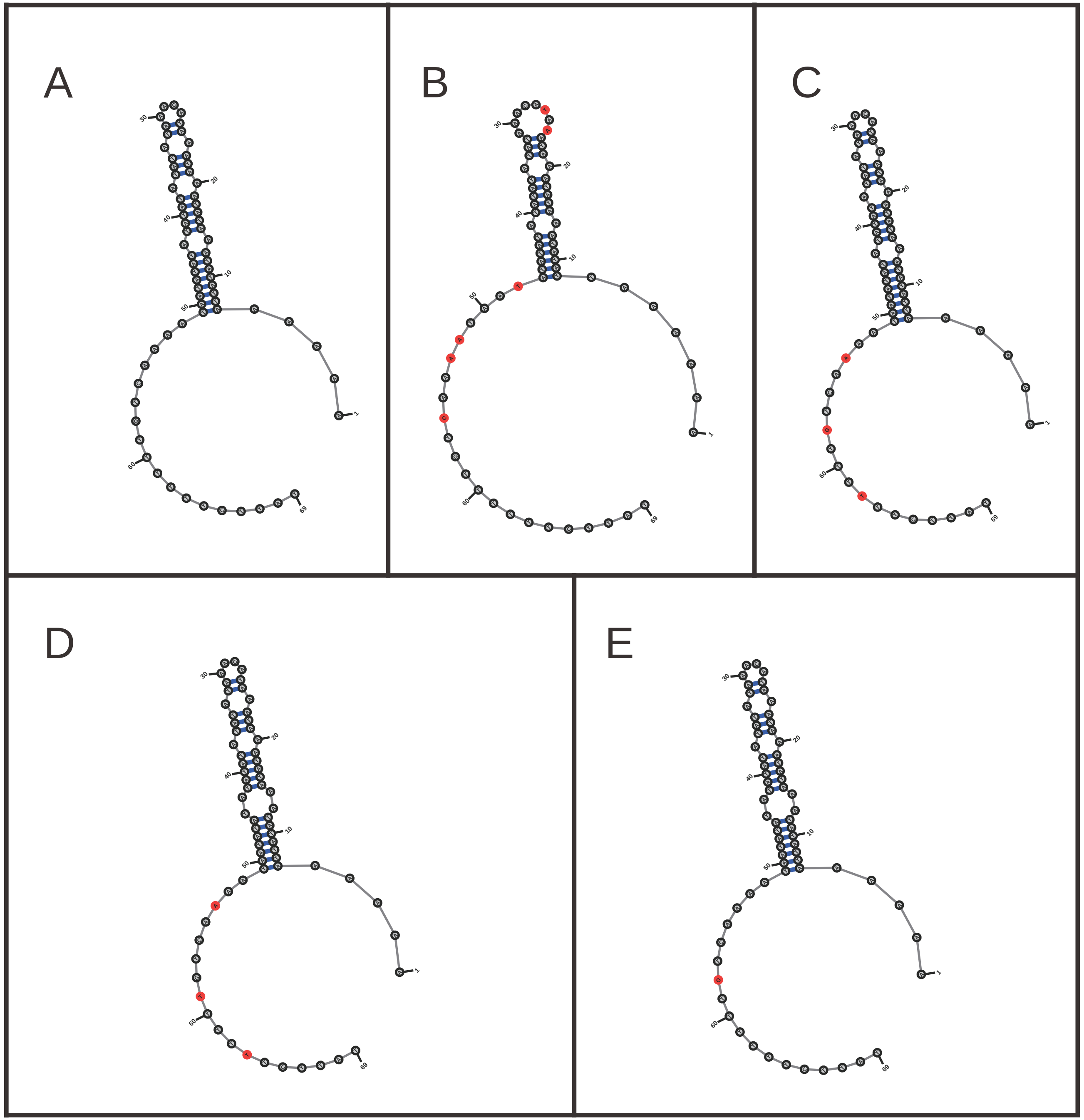

more interior loop after cold stress

PF3D7_1229400

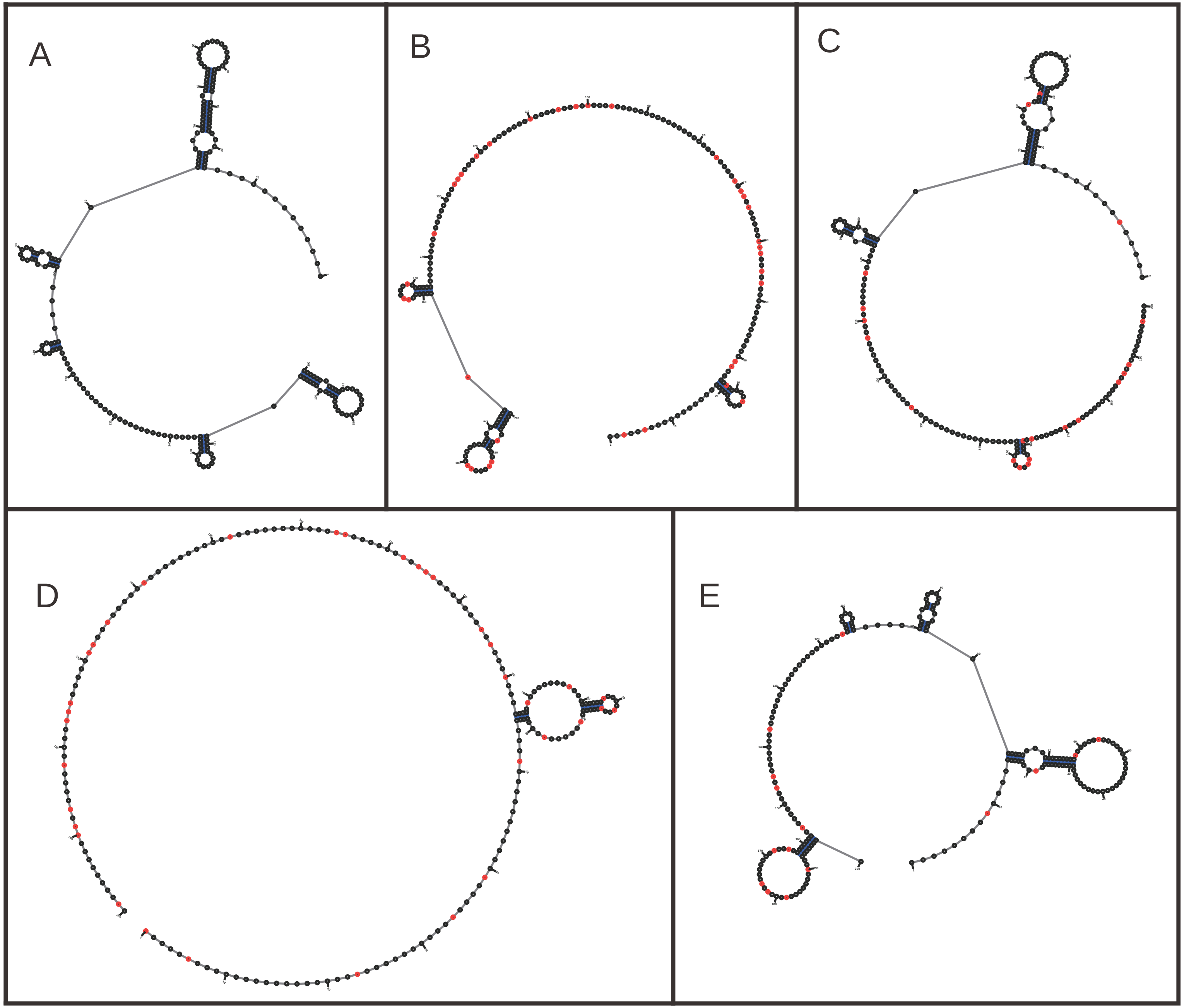

more hairpin loop and interior loop than cold stress

PF3D7_1252300

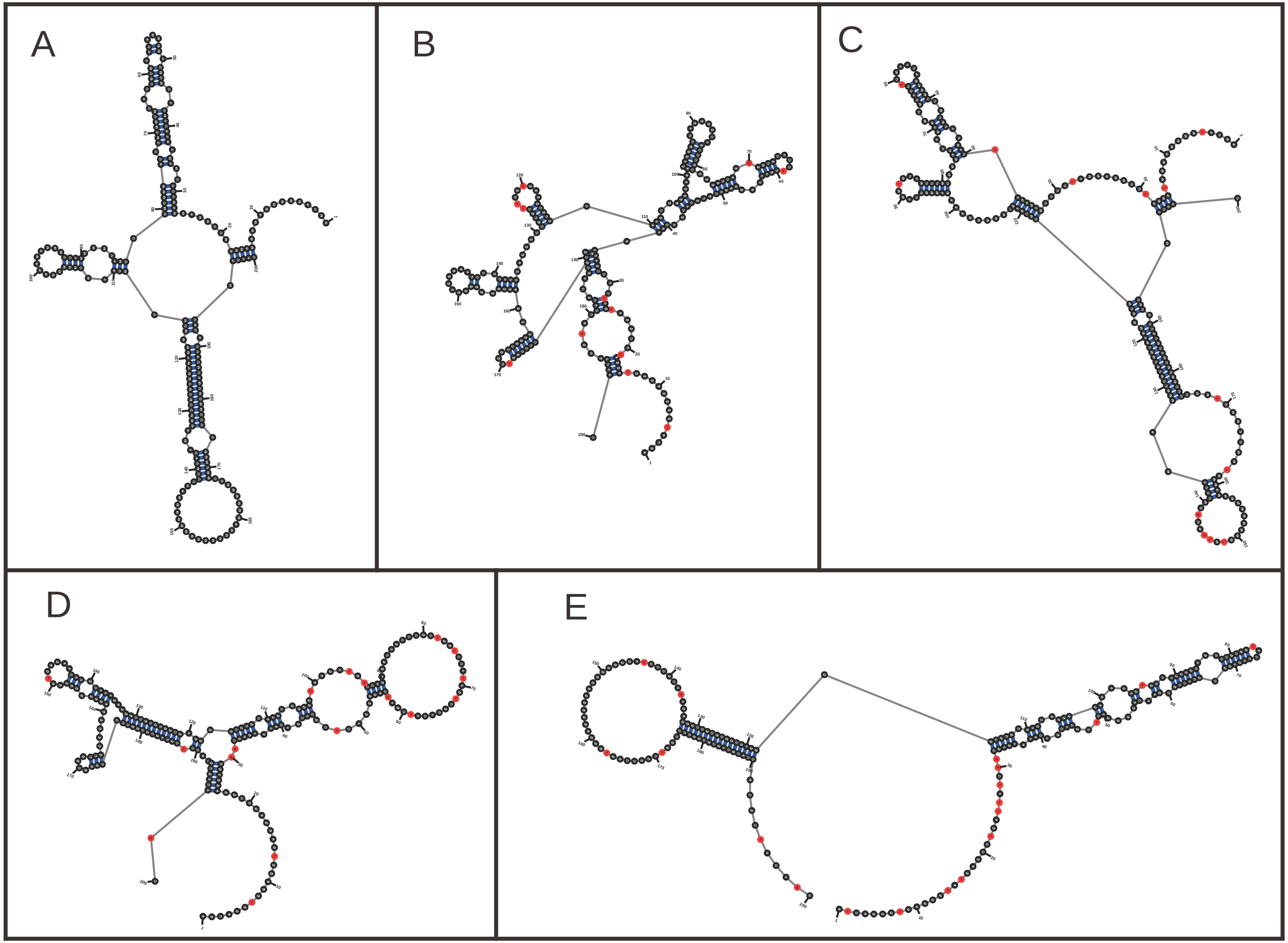

completely different shape

PF3D7_0414800

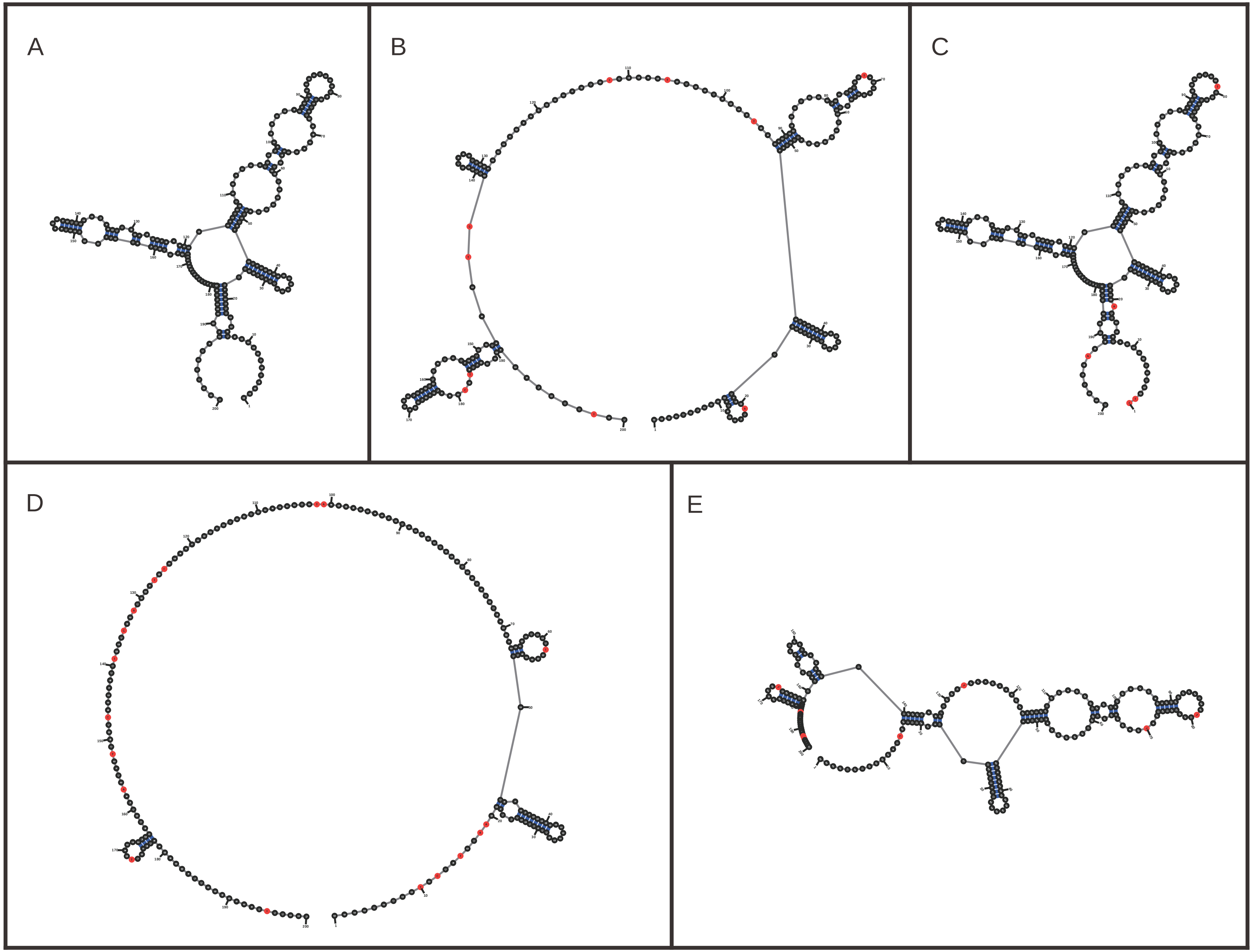

more hairpin loop and interior loop than cold stress

PF3D7_0525700

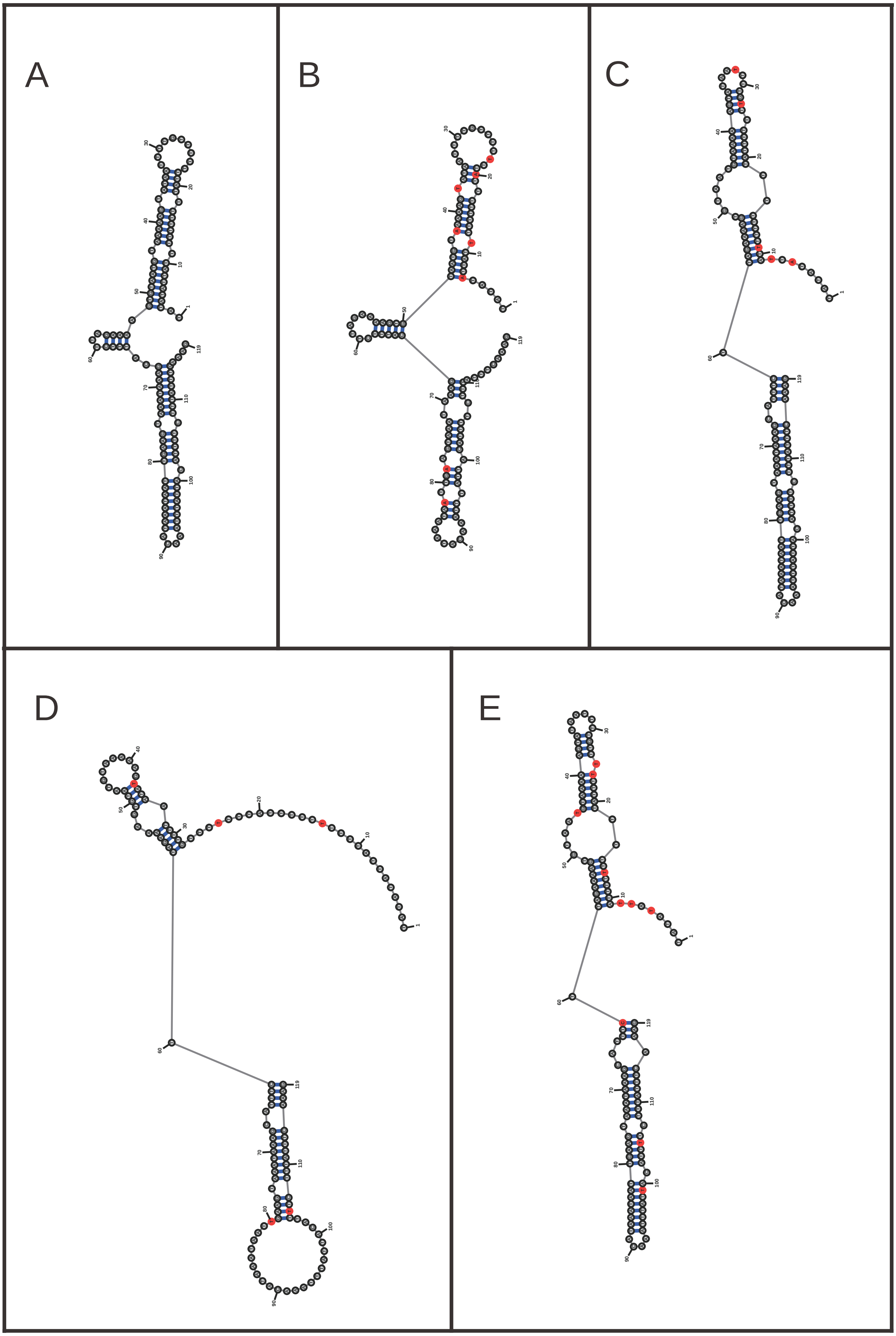

more hairpin loop and interior loop than cold stress

PF3D7_0731400

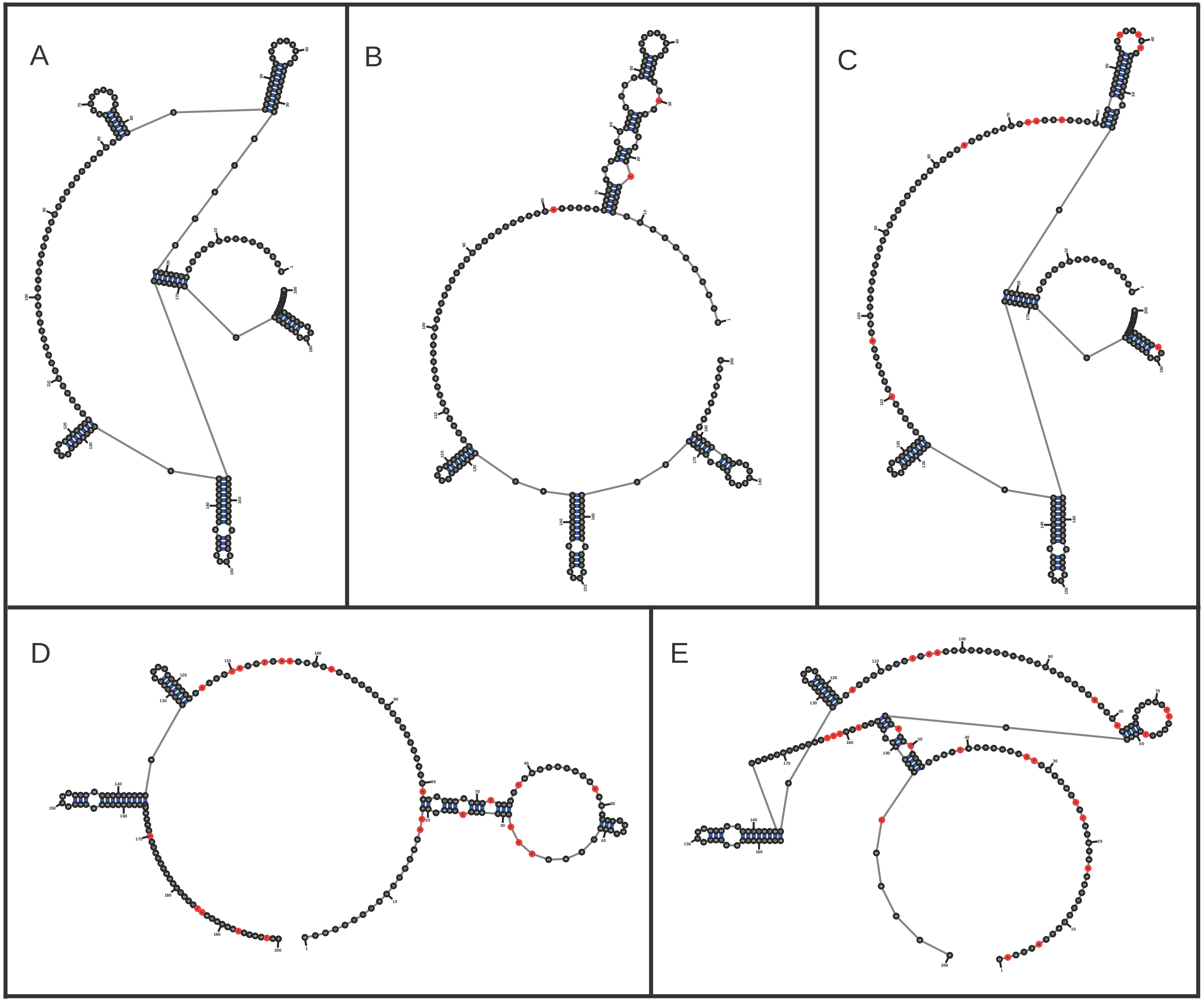

more hairpin loop and interior loop than cold stress

PF3D7_0930200

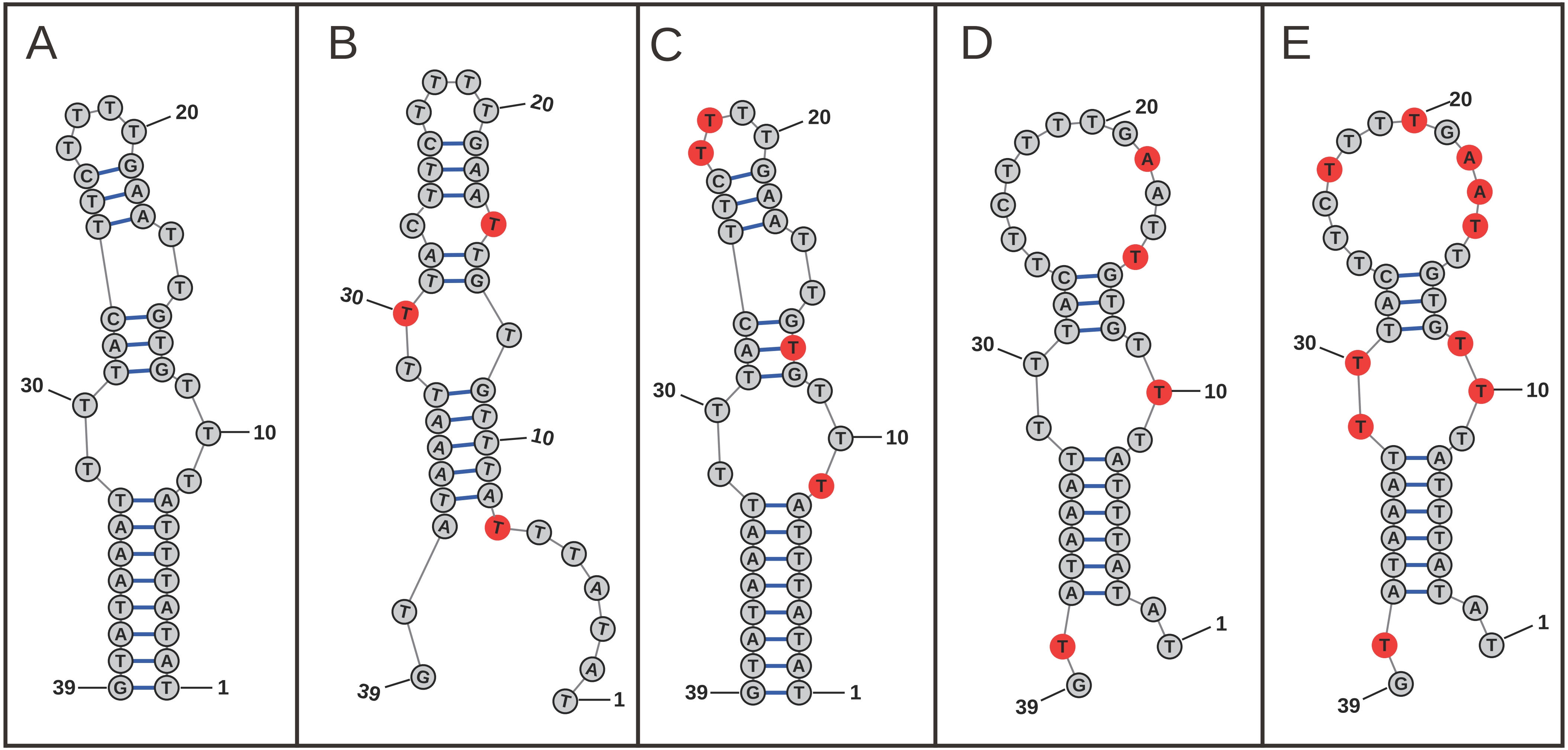

more interior loop than cold stress

PF3D7_0206800

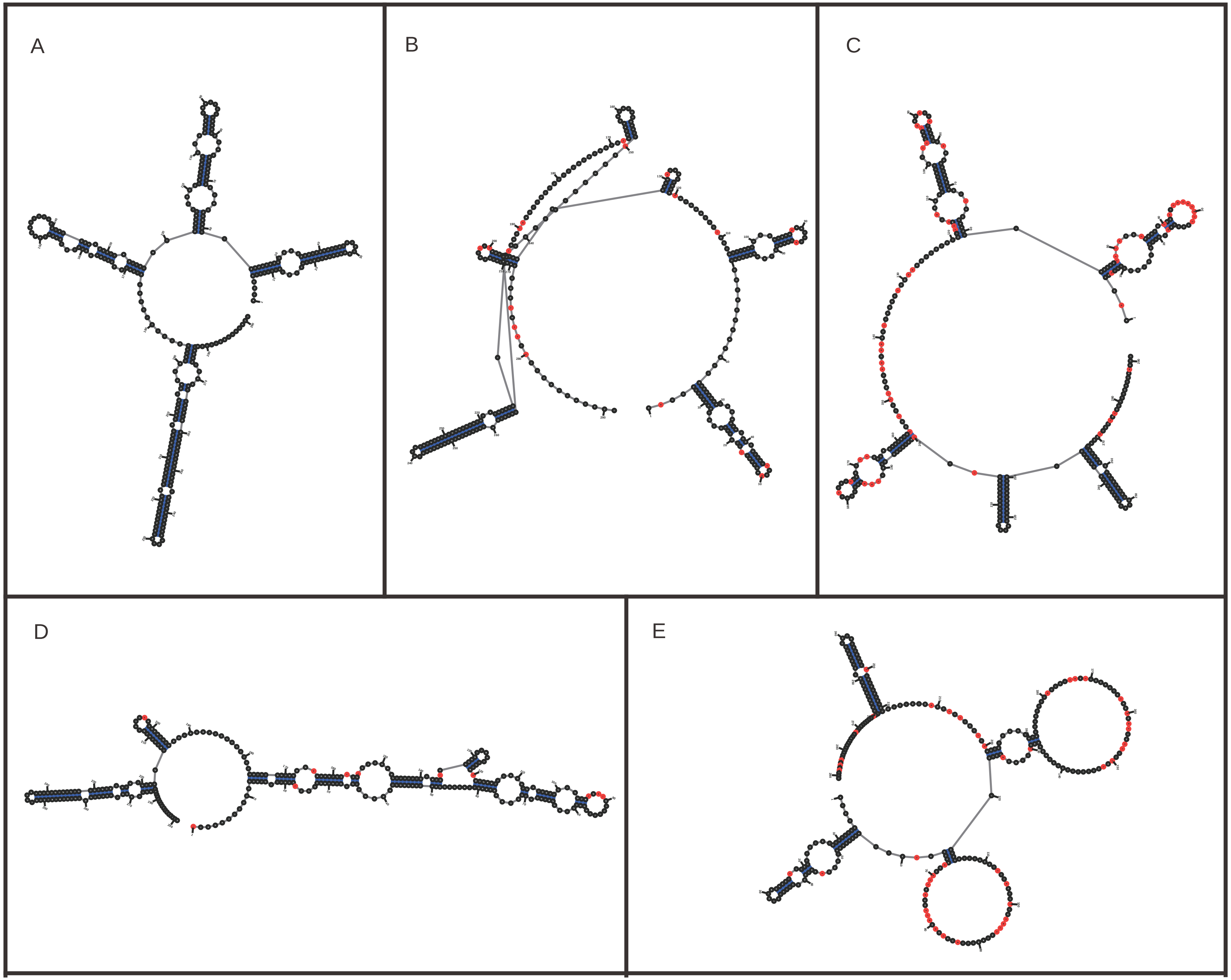

completely different shape

PF3D7_0400200

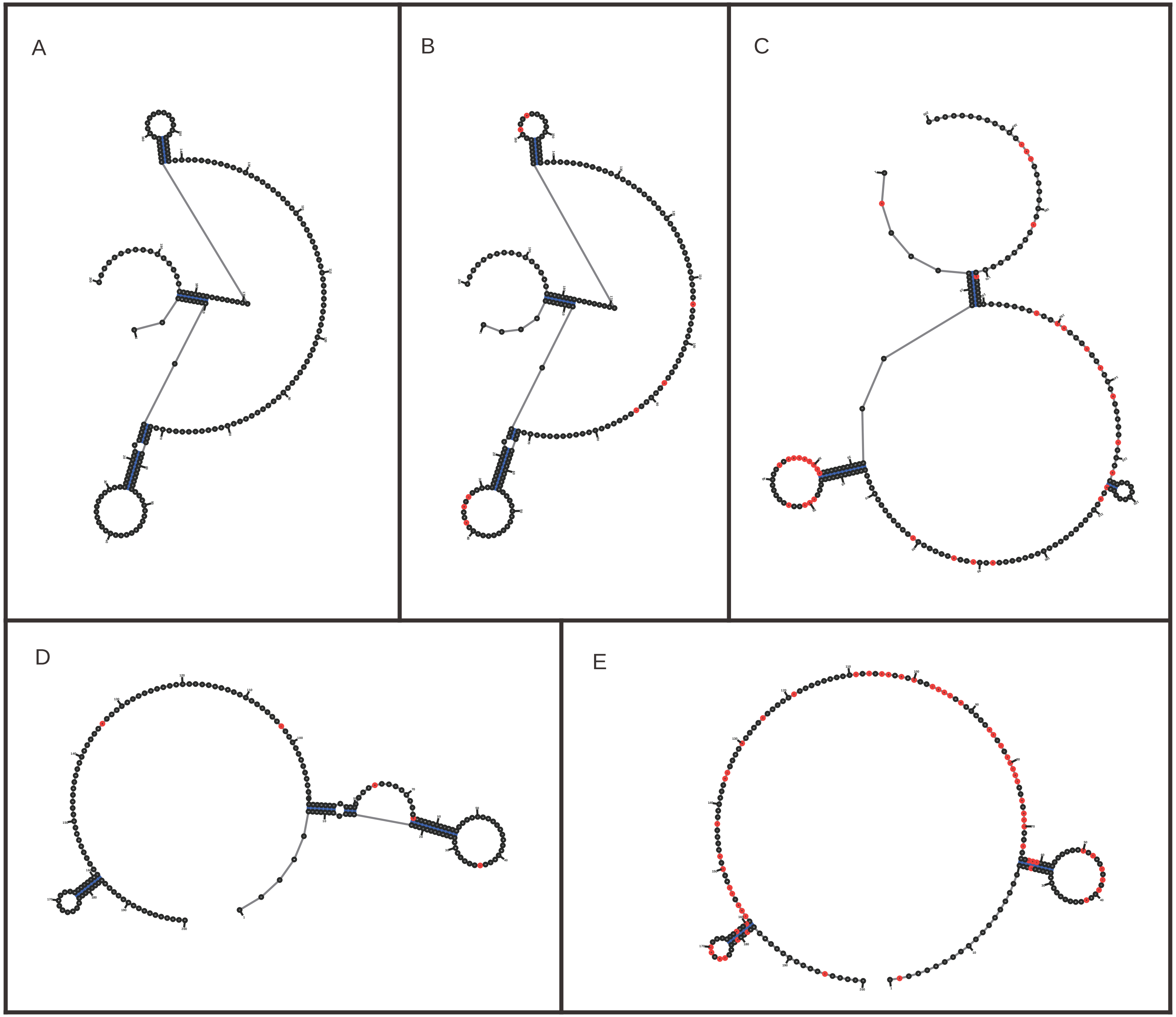

completely different shape

PF3D7_0423400

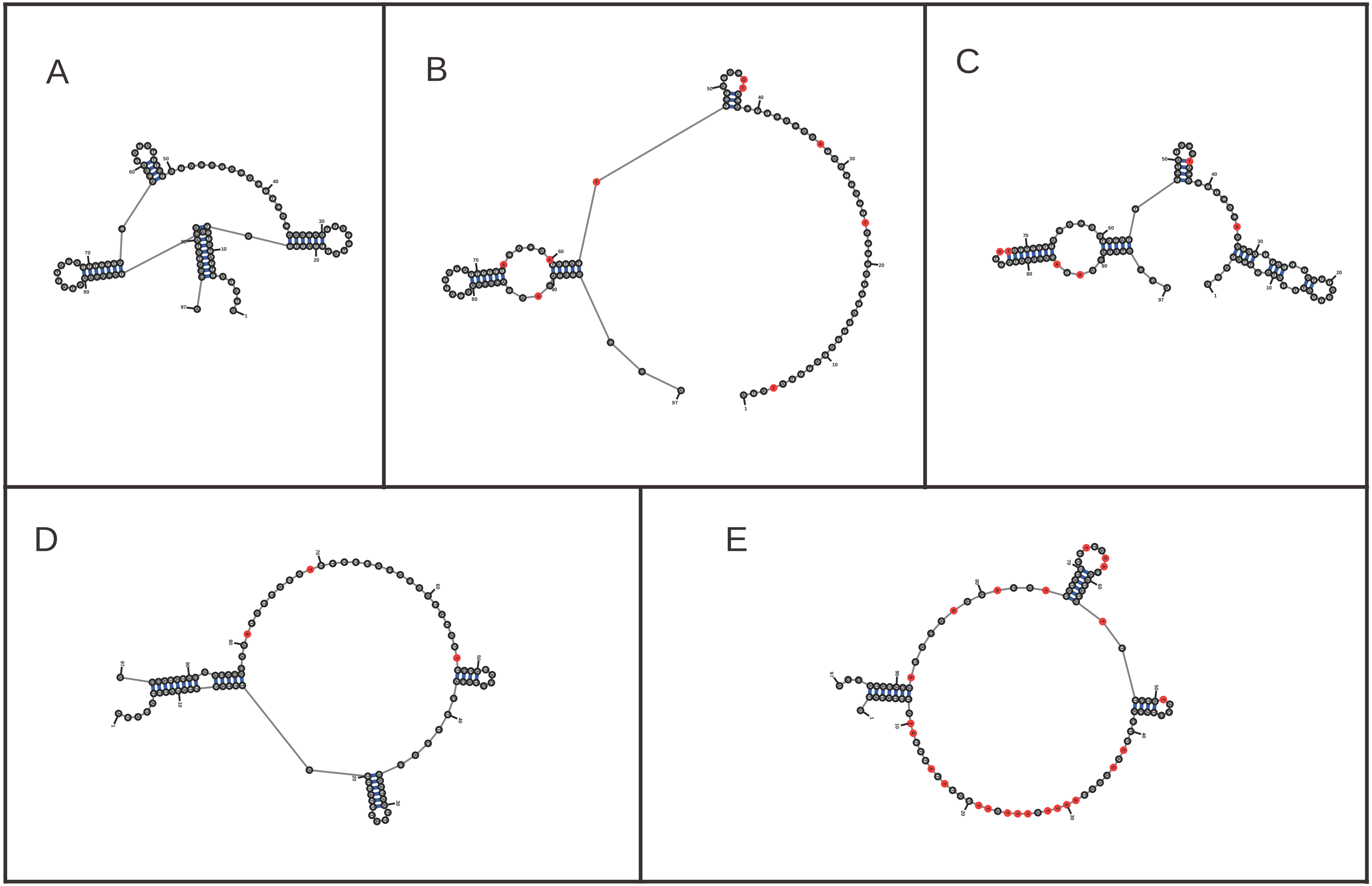

more multi-btanch loop after cold response

PF3D7_0423700

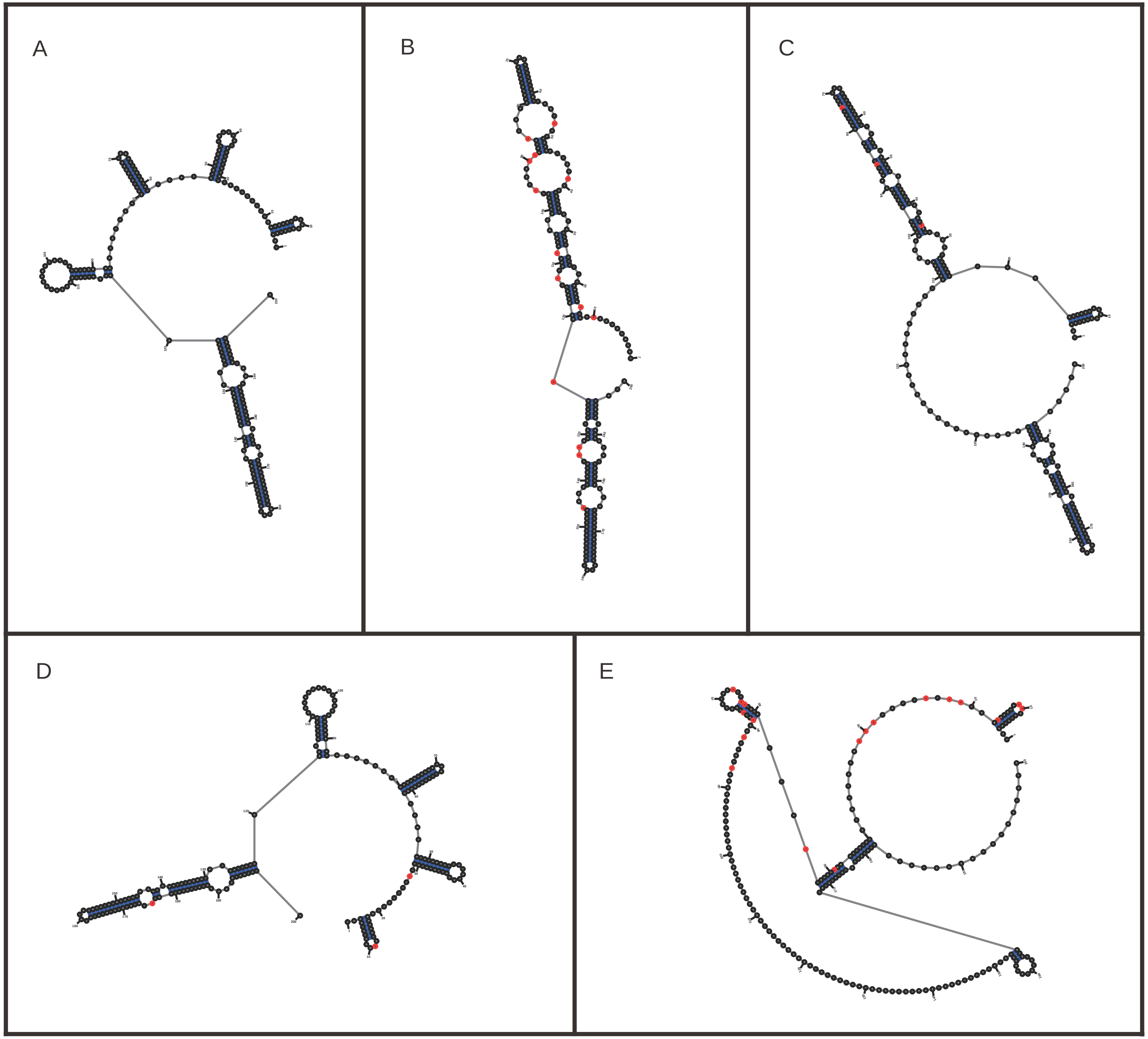

totally different

PF3D7_0501600

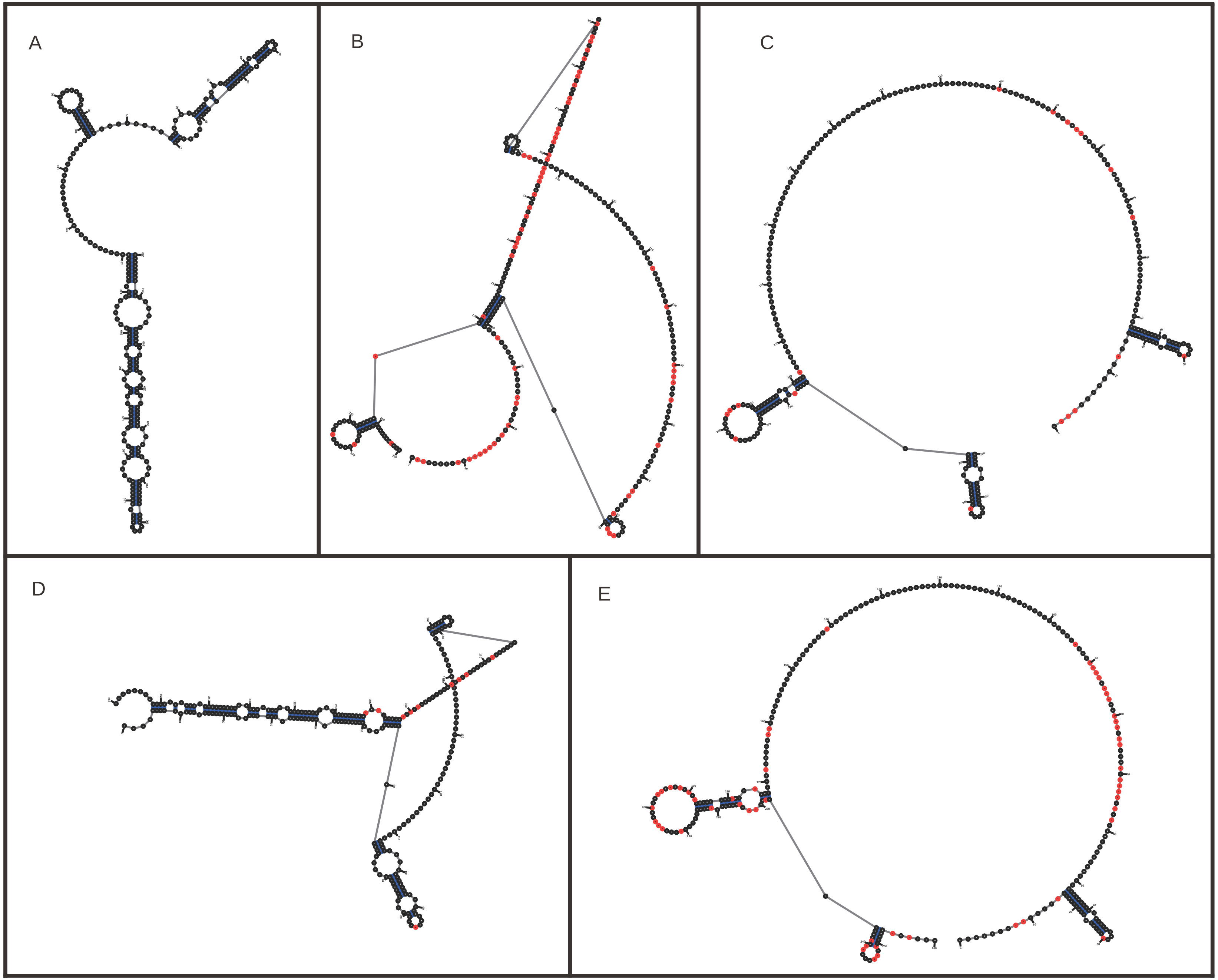

more hairpin loop and interior loop after cold response

PF3D7_0511600

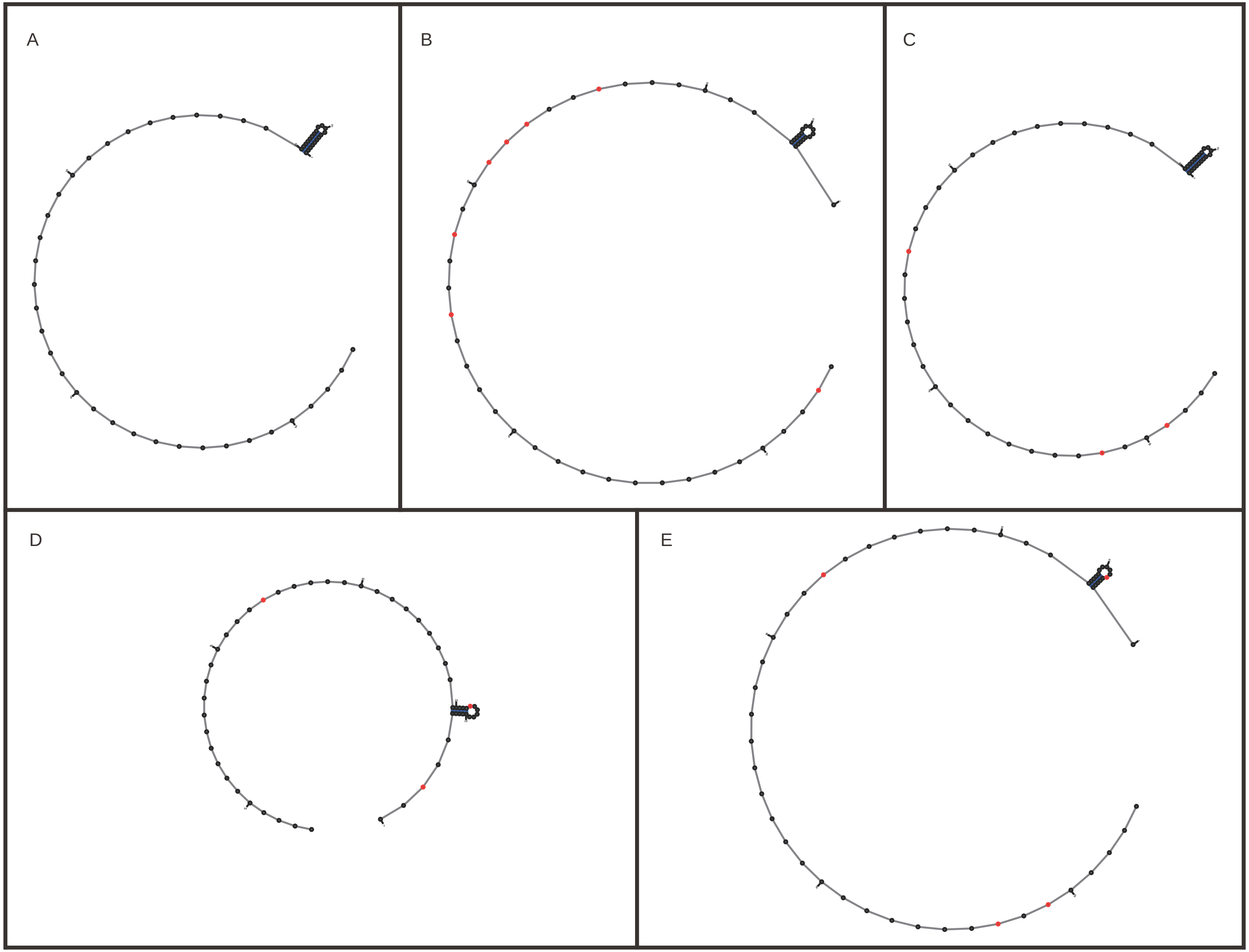

A hairpin exist in different locations

PF3D7_0728400

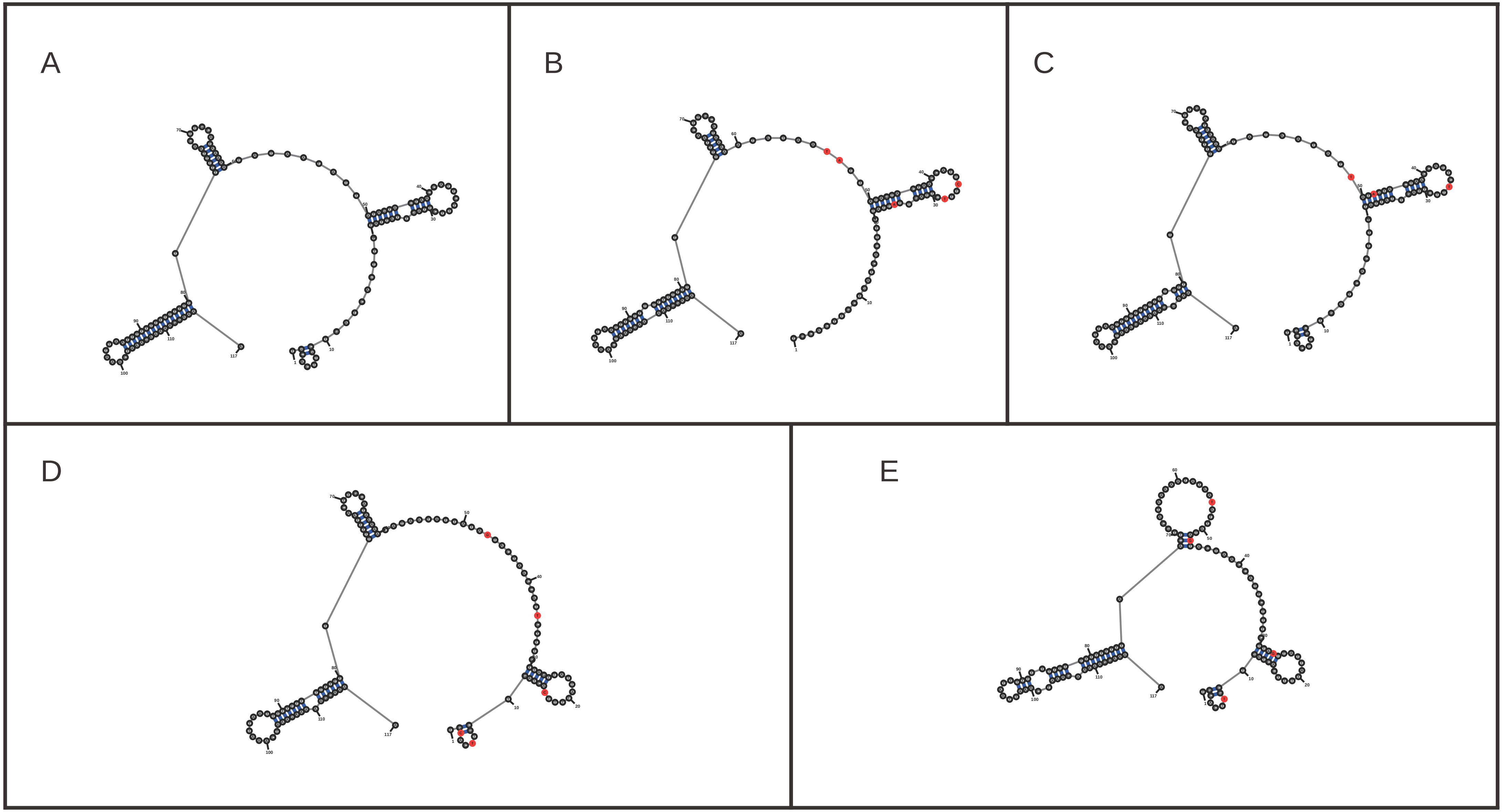

more hairpin loop and less interior loop after cold response

PF3D7_1015900

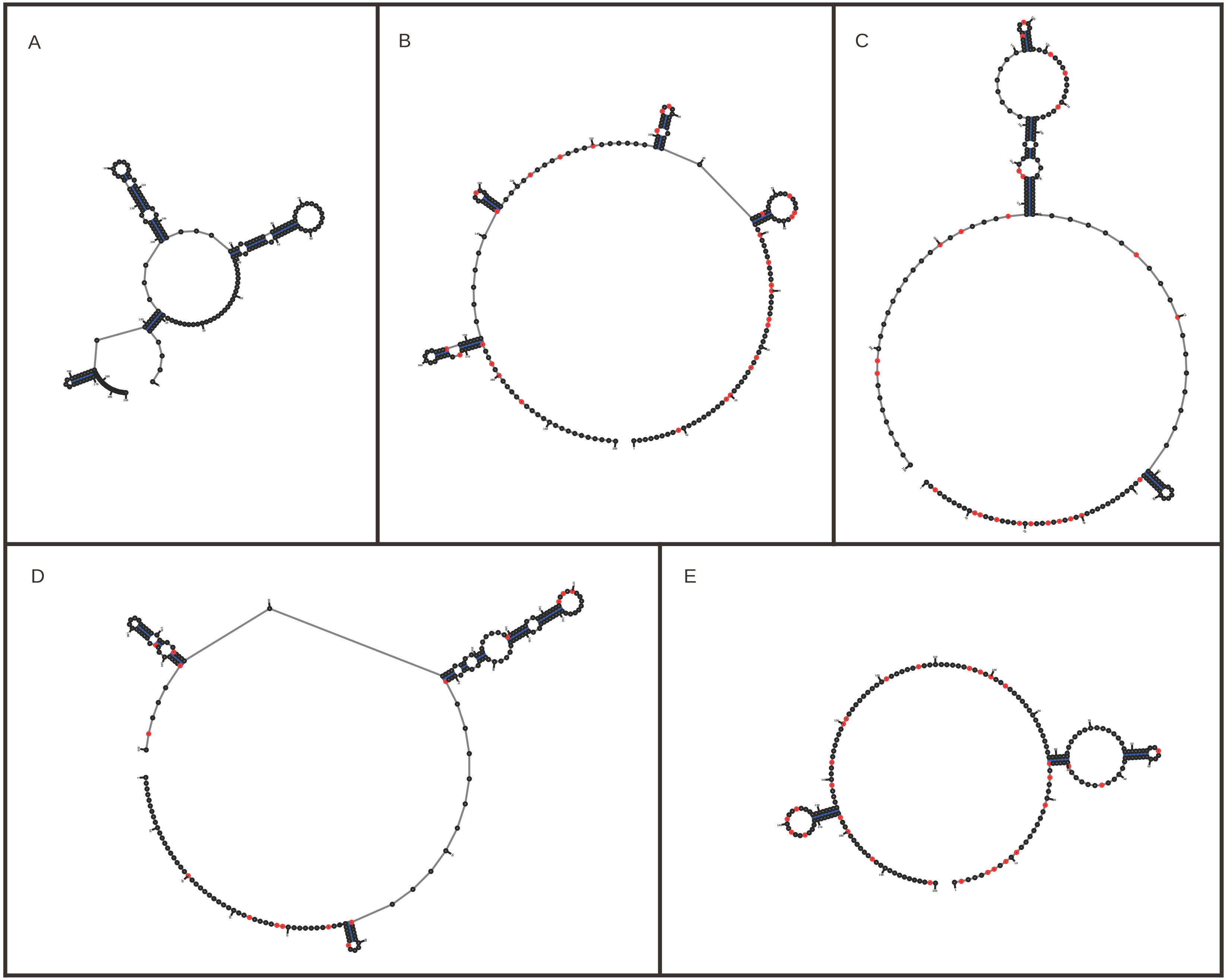

more interior loop after cold response

PF3D7_1029600

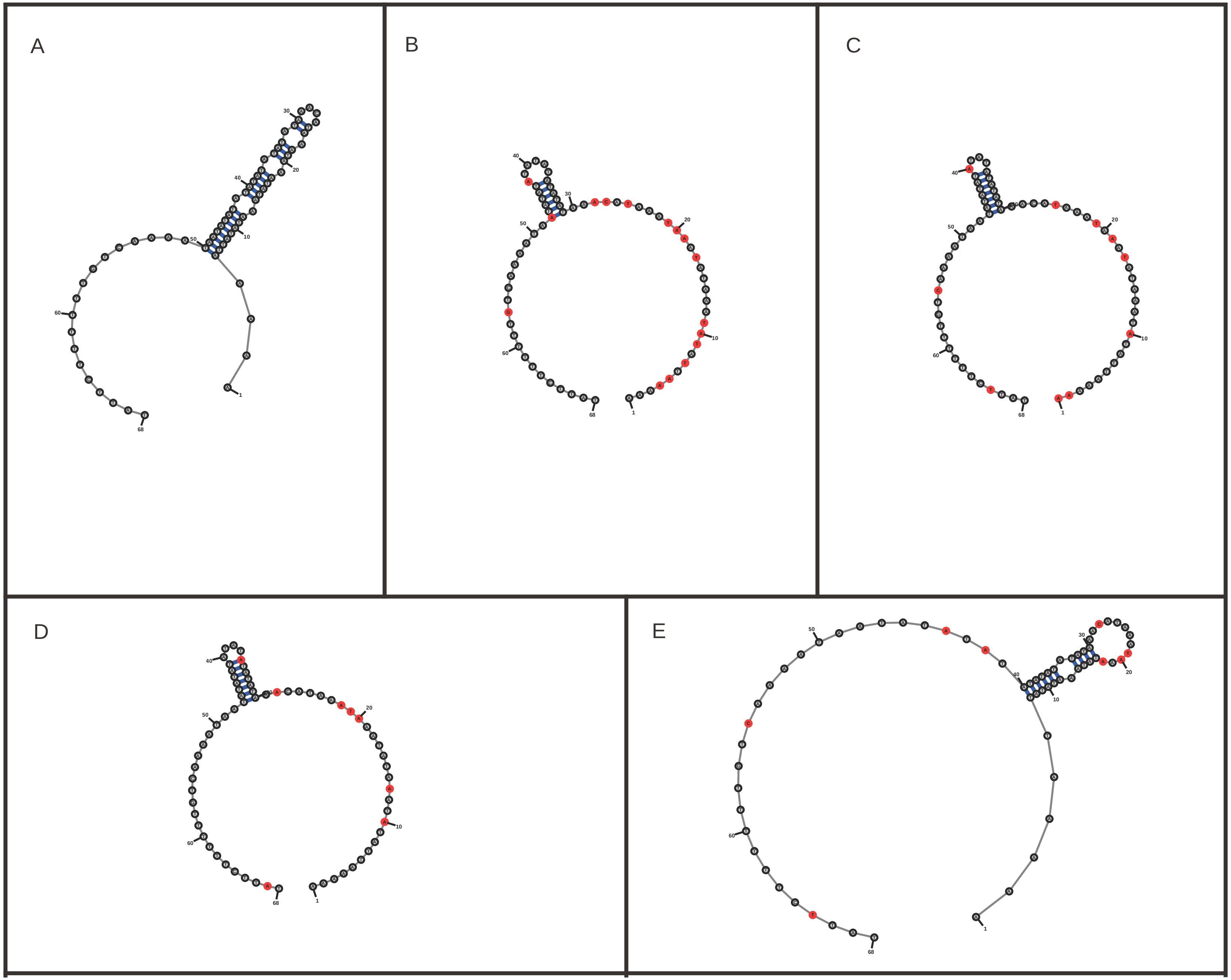

similar structure

PF3D7_1229400

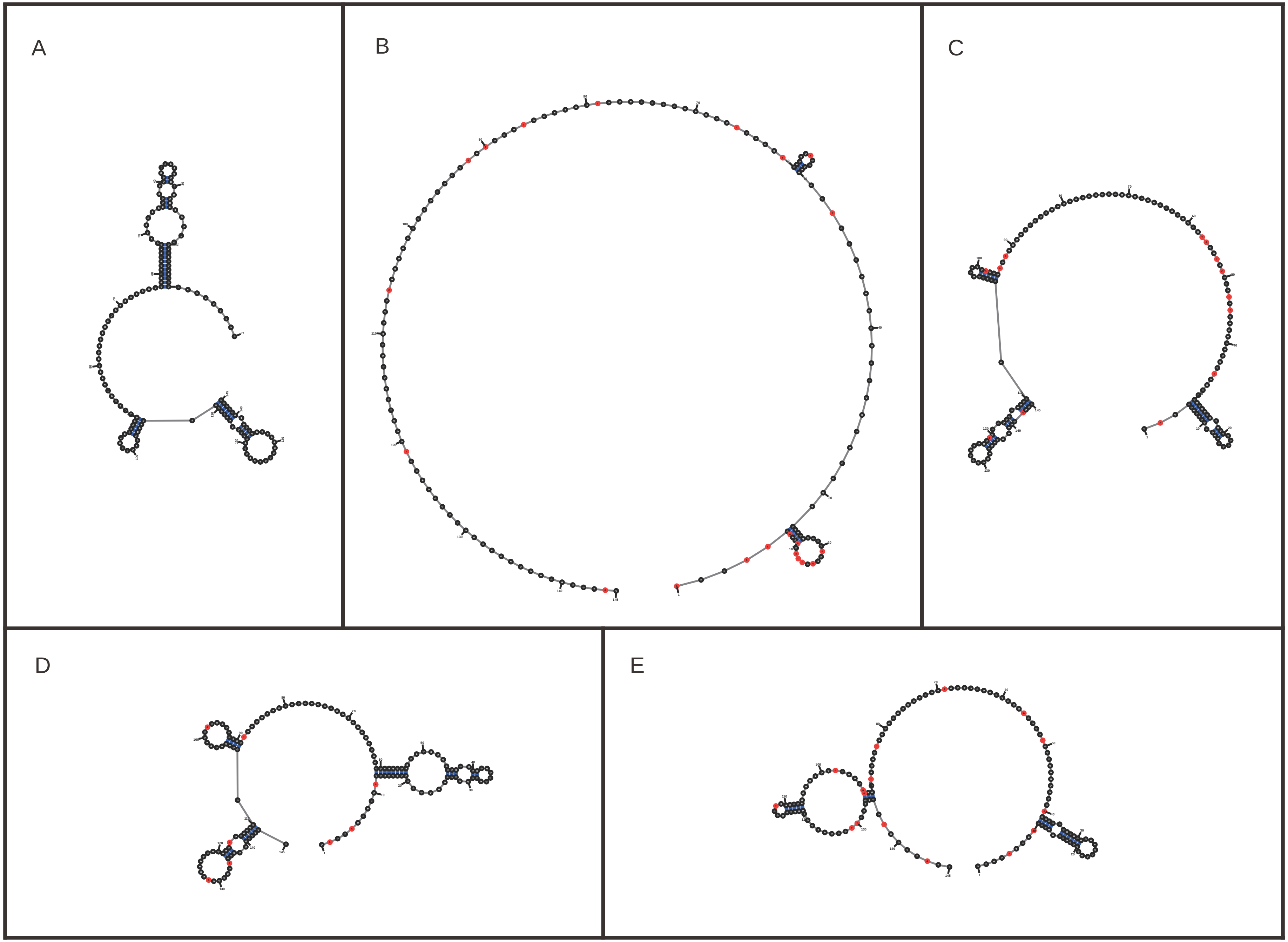

more hairpin loop and interior loop after cold response

PF3D7_1229700

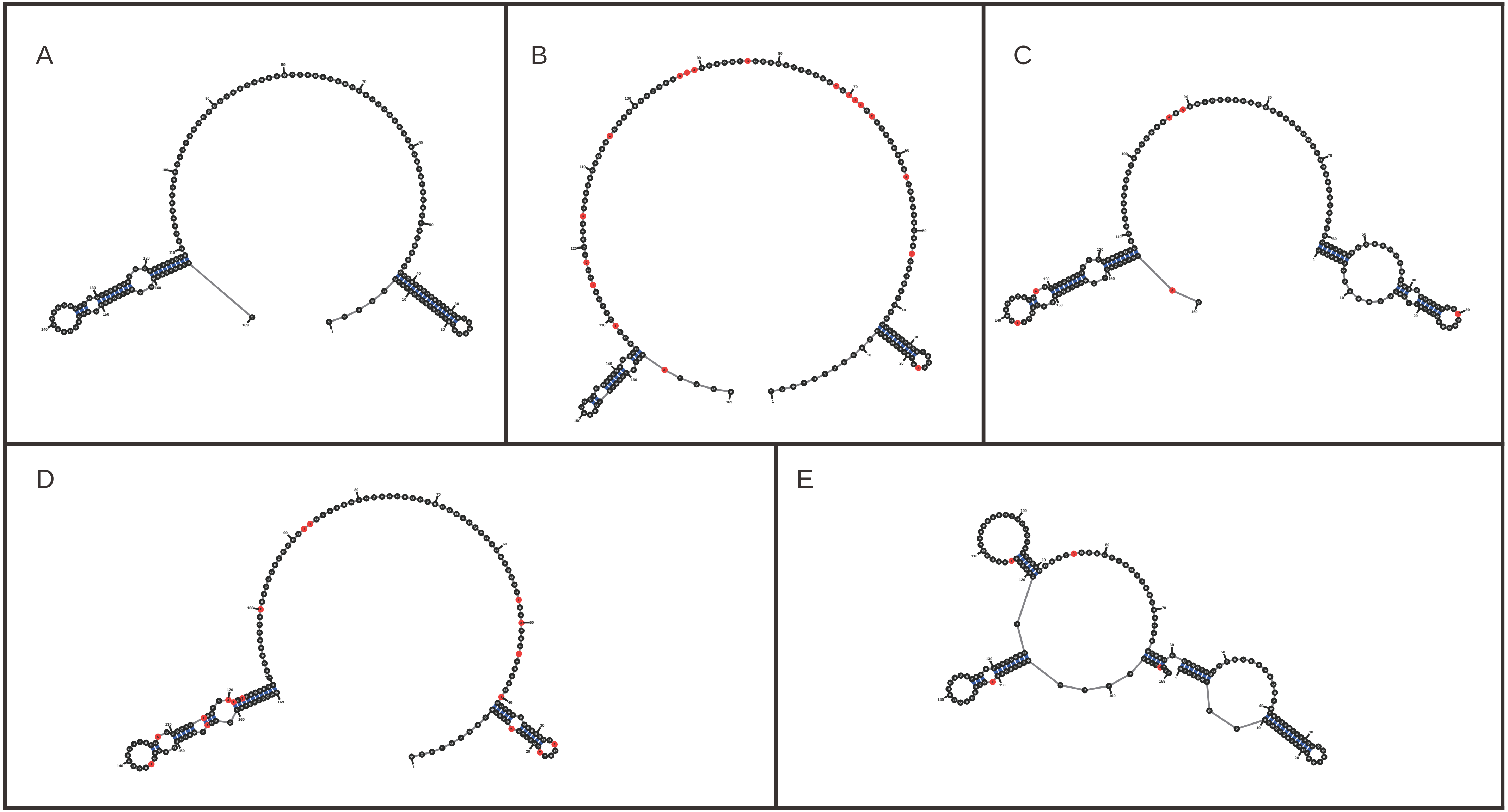

more interior loop after cold response

PF3D7_1449200

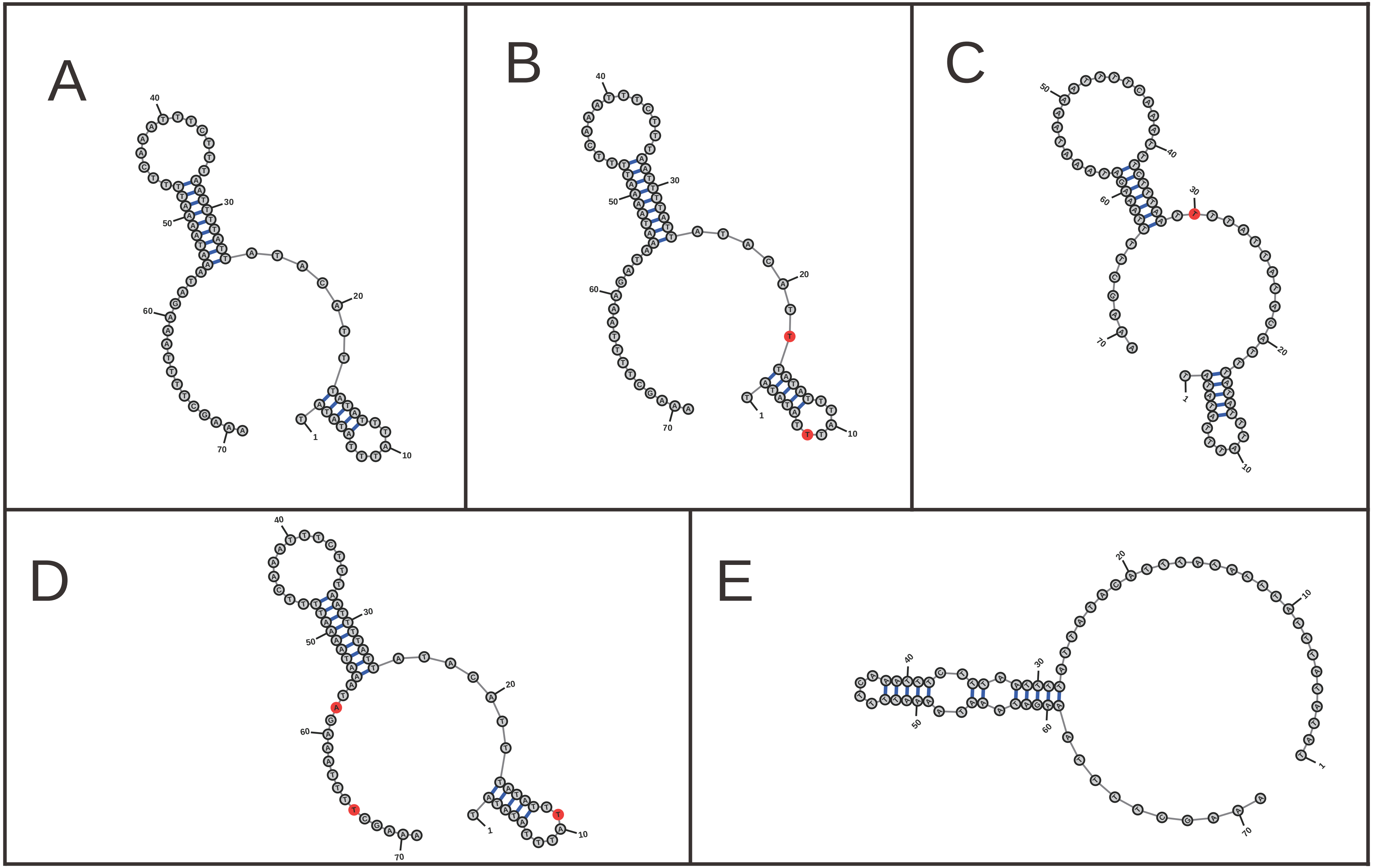

same structure

PF3D7_1470400

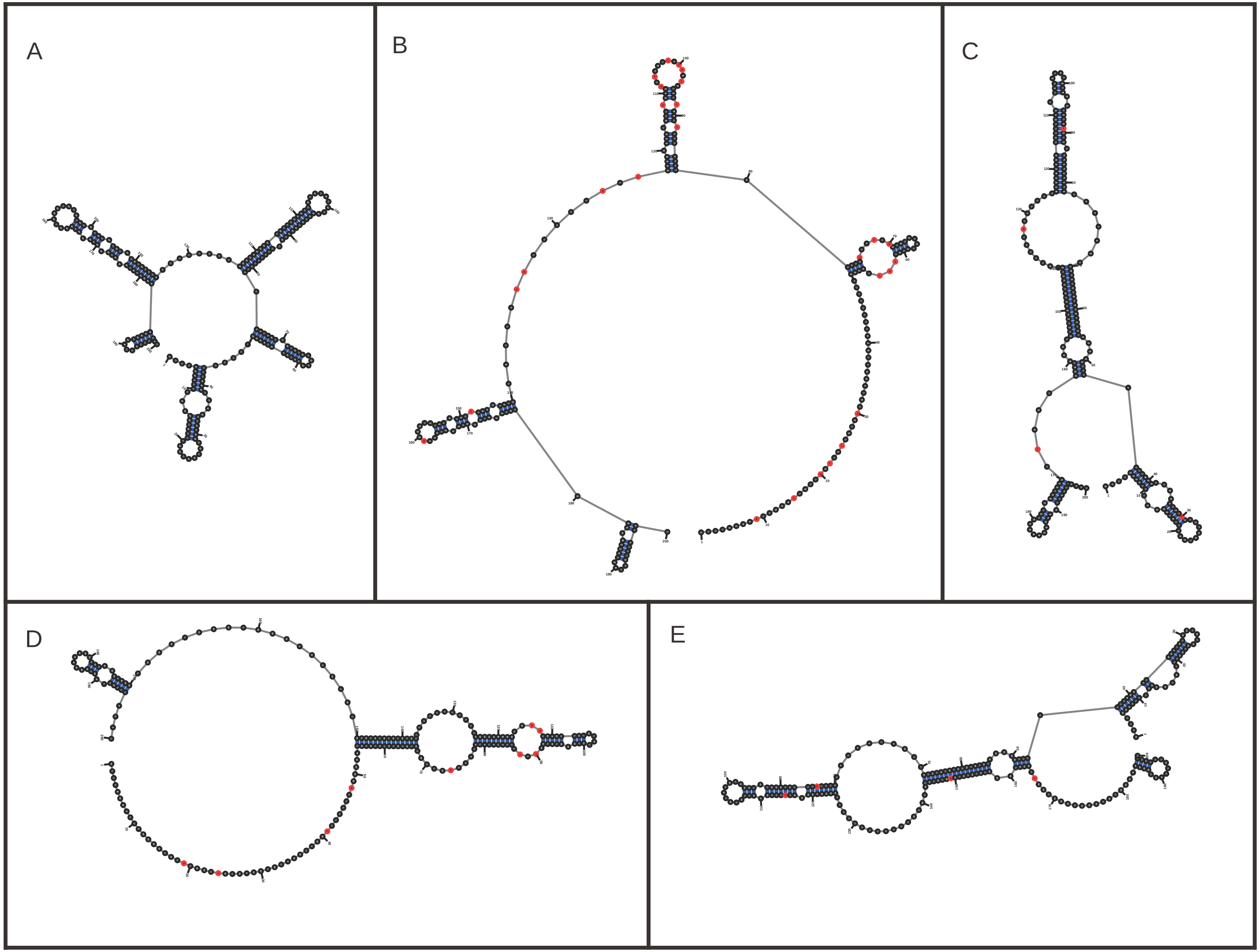

more hairpin loop and interior loop than cold stress

PF3D7_0108000

A	B	C
Those structures contain no pairs		

D	E

PF3D7_0207000

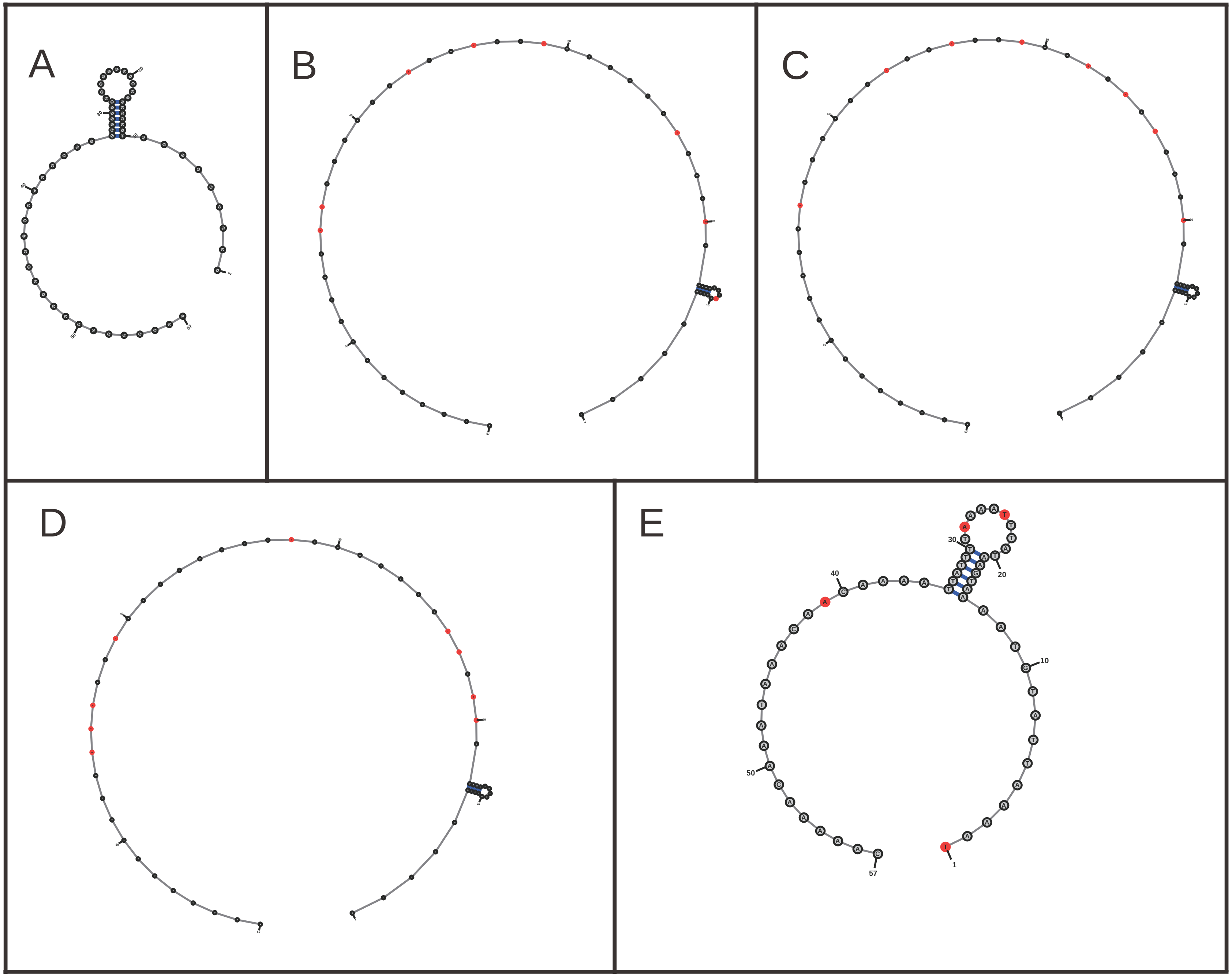

PF3D7_0320900

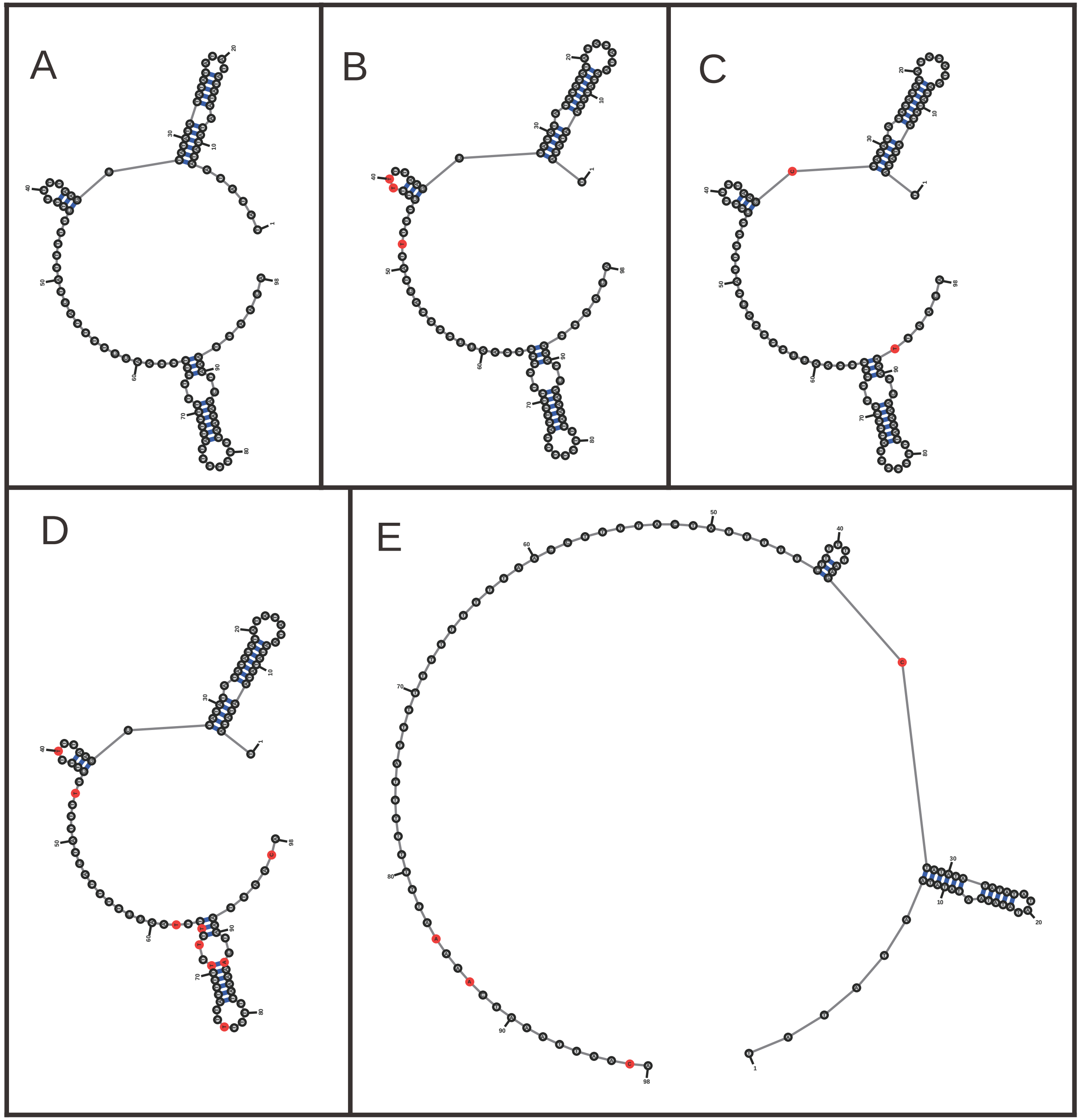

PF3D7_0404900

A

B

Those structures contain no pairs

C

D

E

PF3D7_0501500

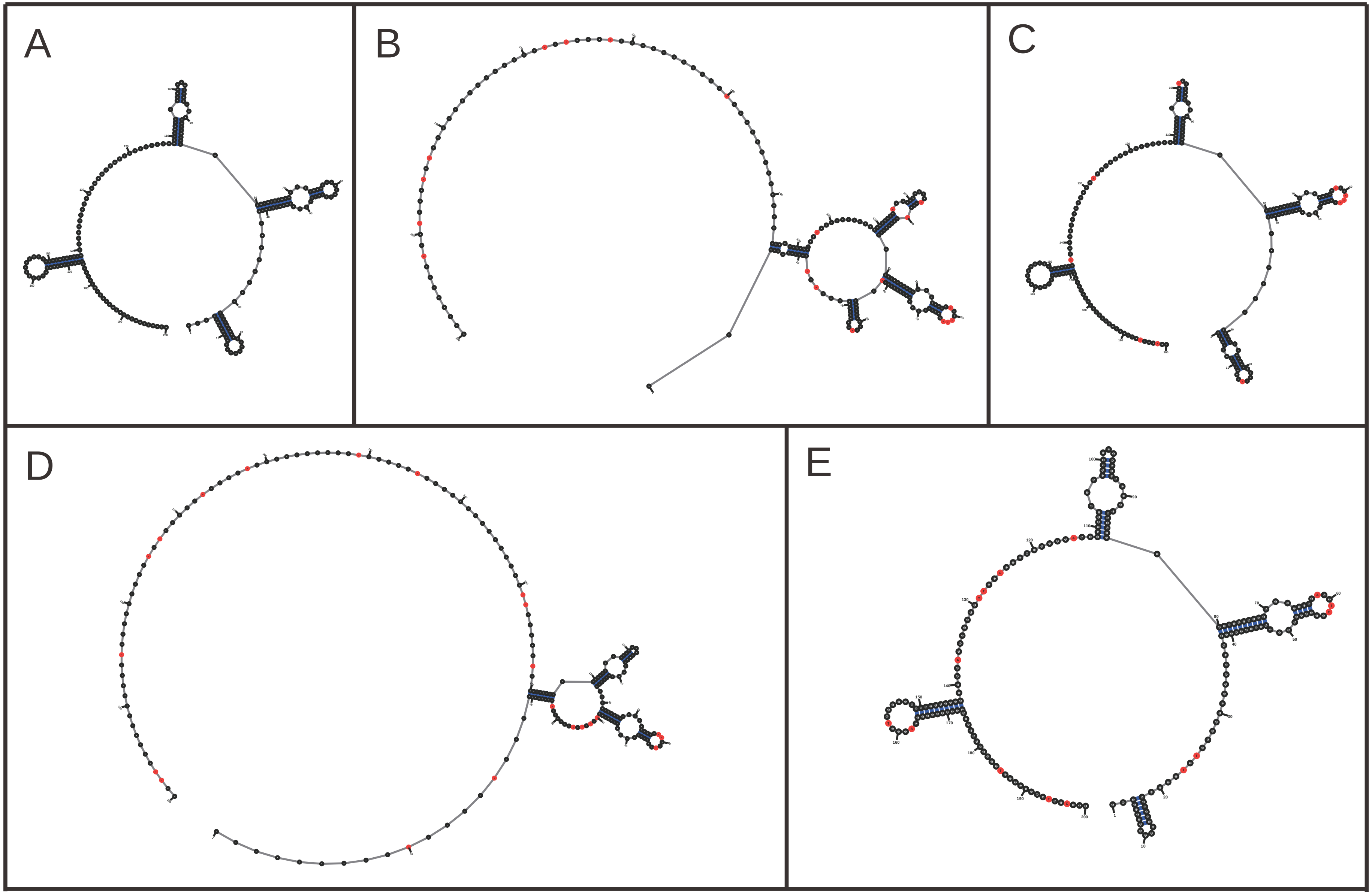

PF3D7_0610400

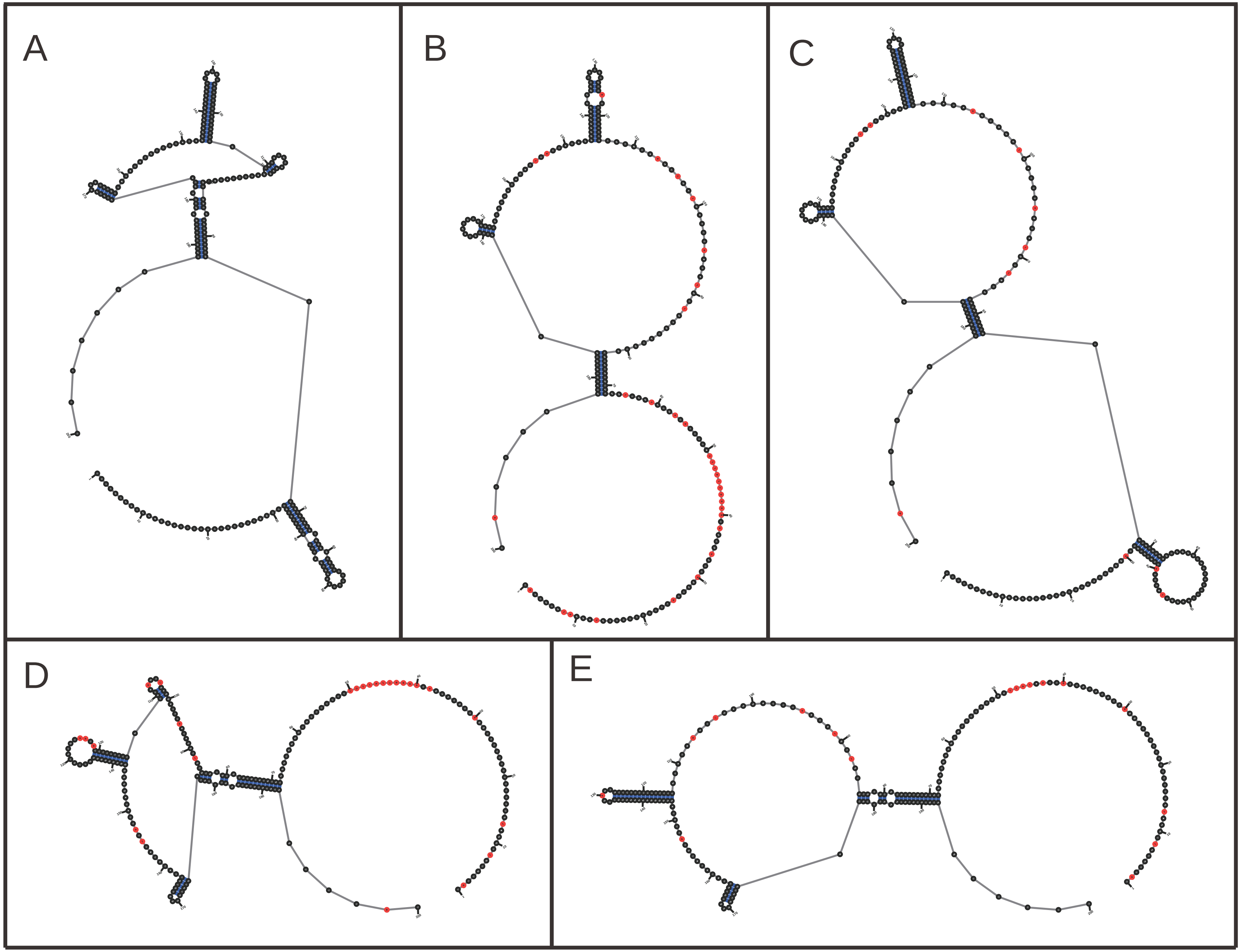

PF3D7_0725400

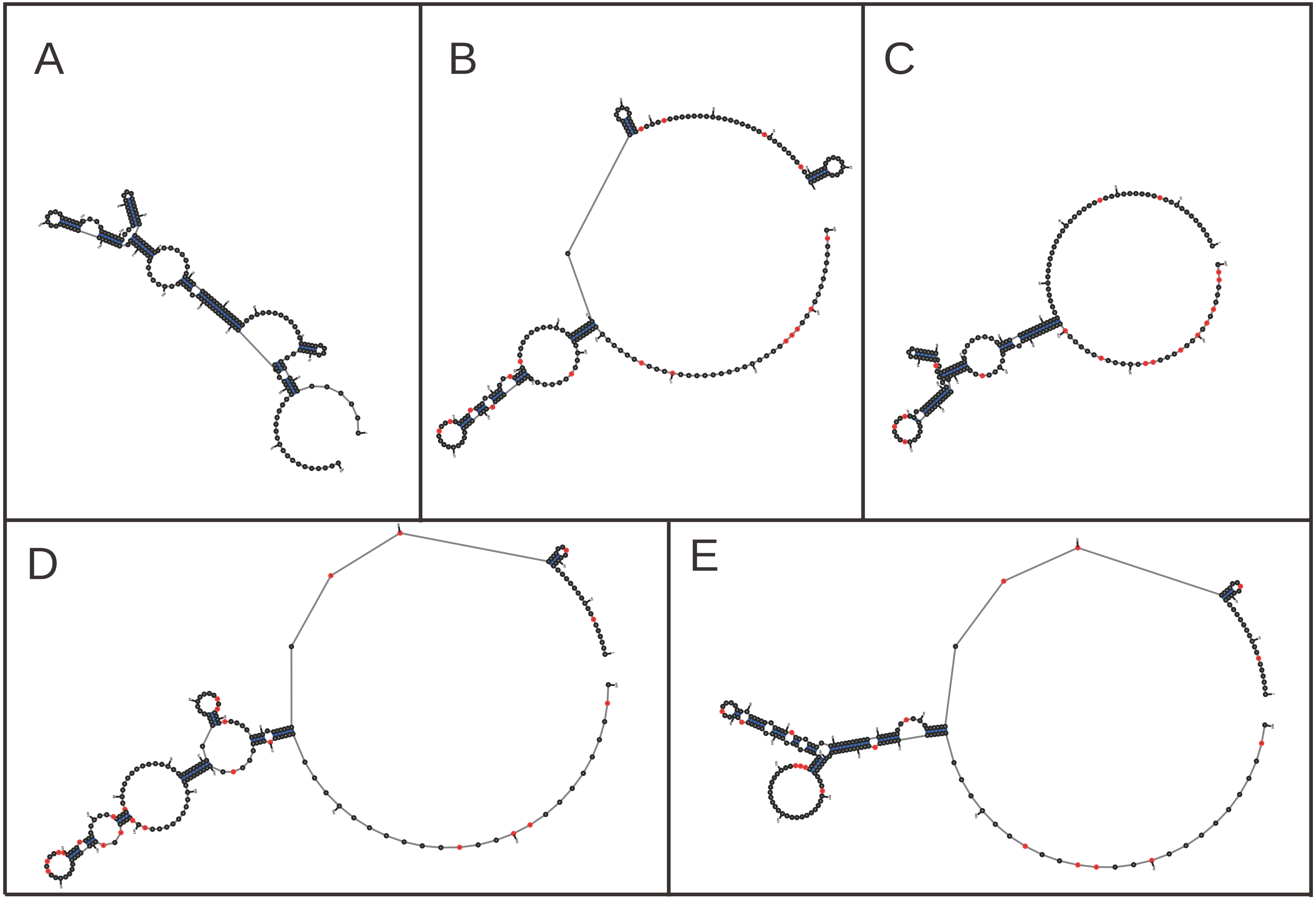

PF3D7_0818200

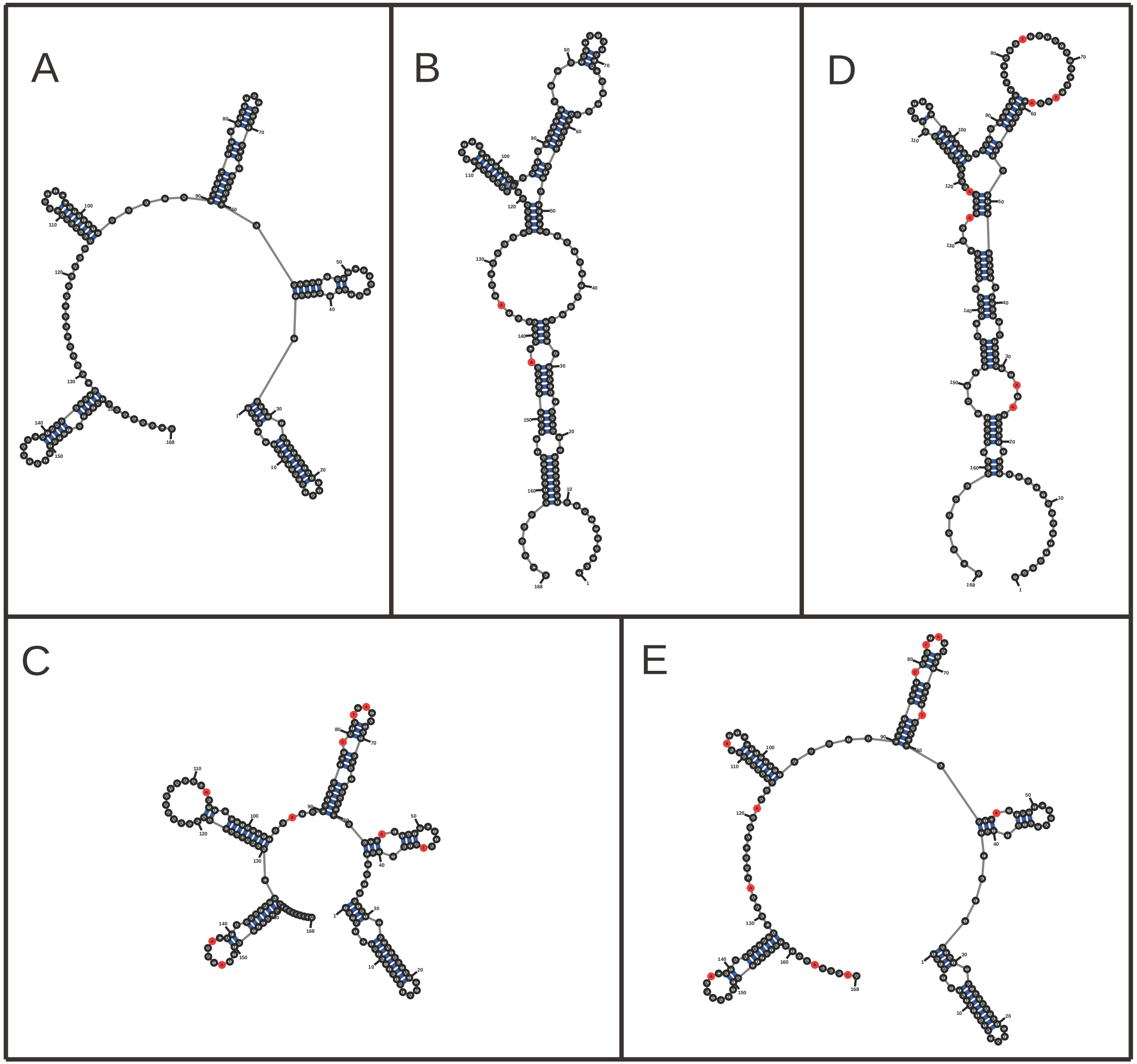

PF3D7_0929500

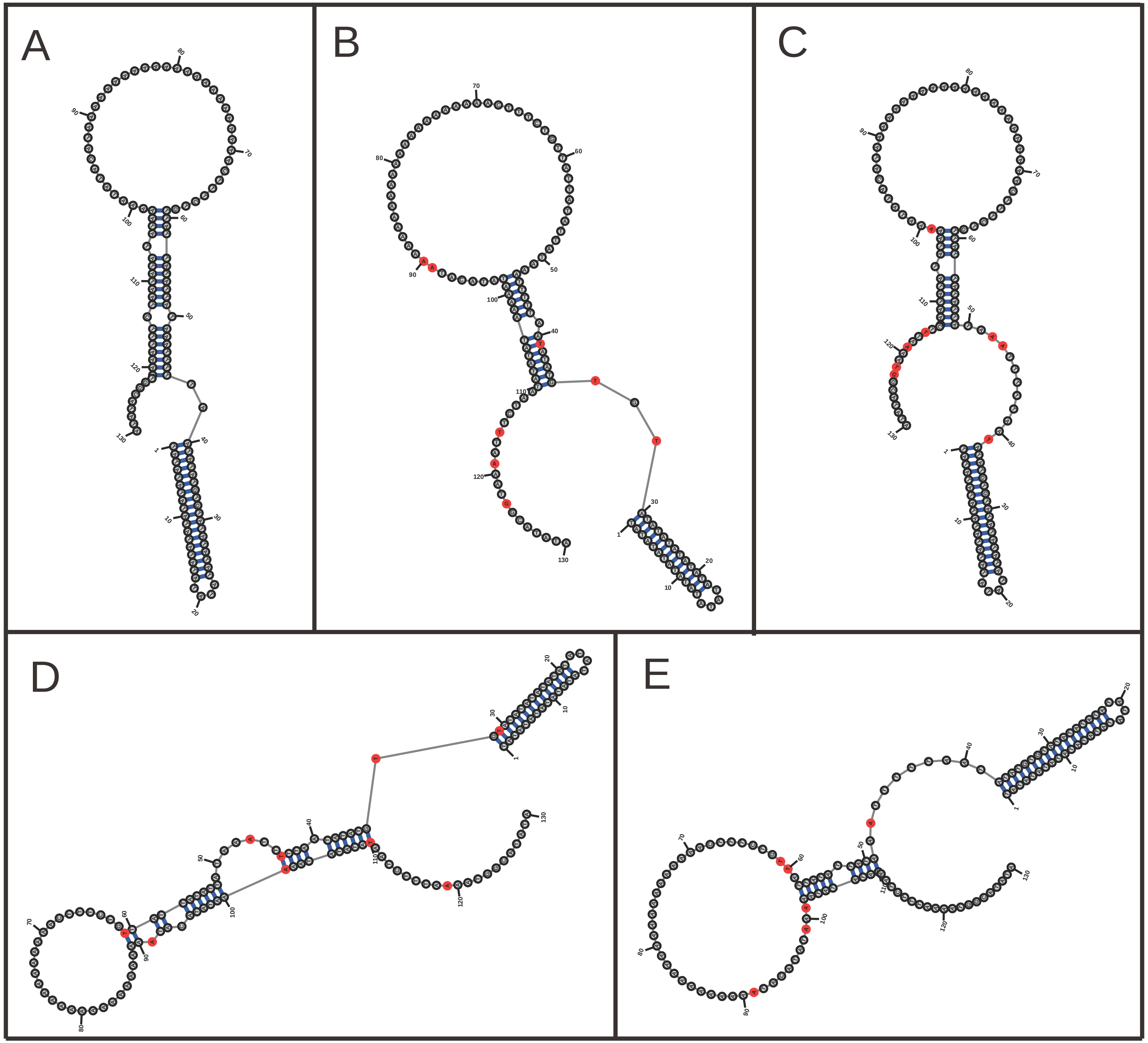

PF3D7_1012400

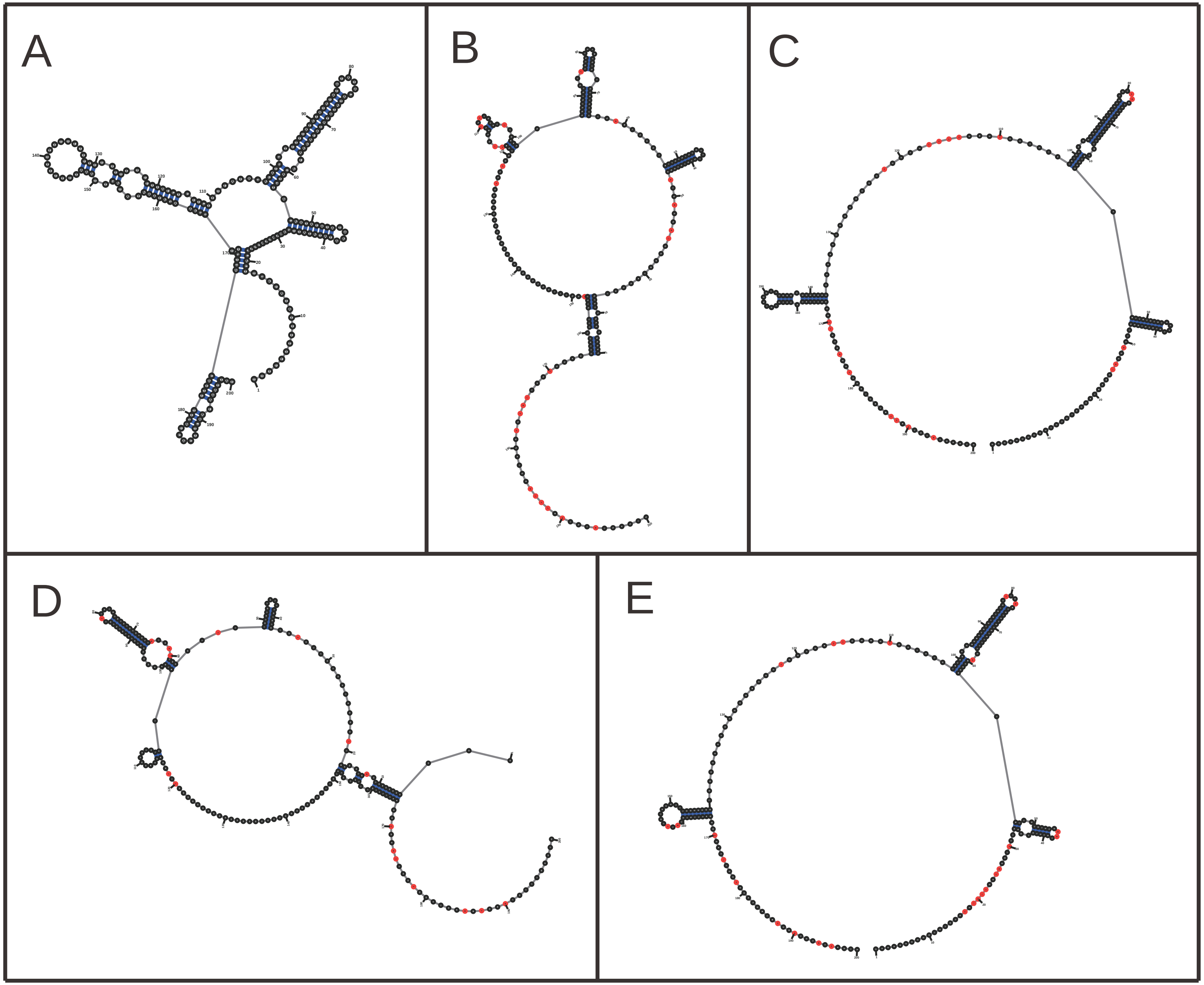

PF3D7_1103100

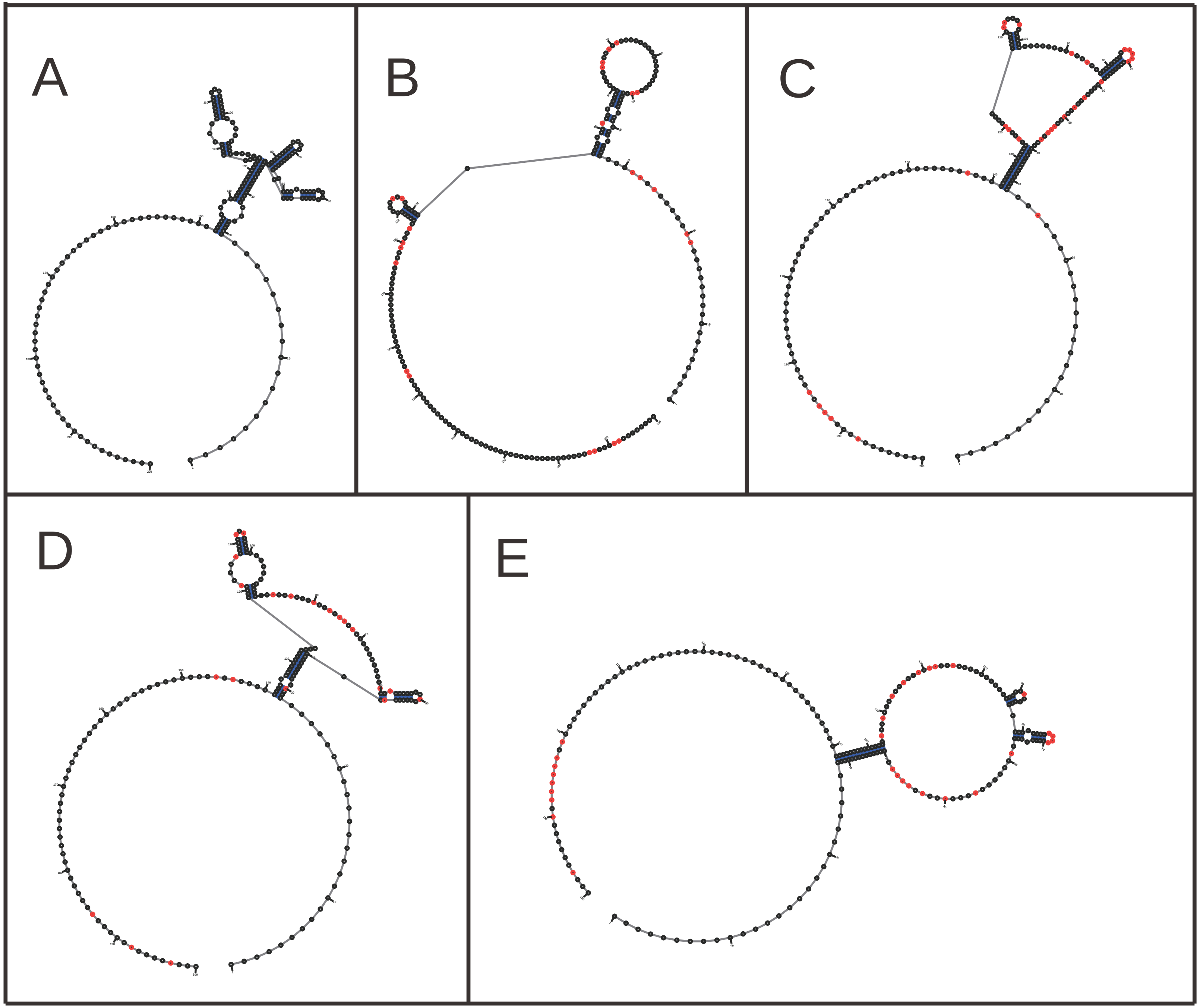

PF3D7_1246200

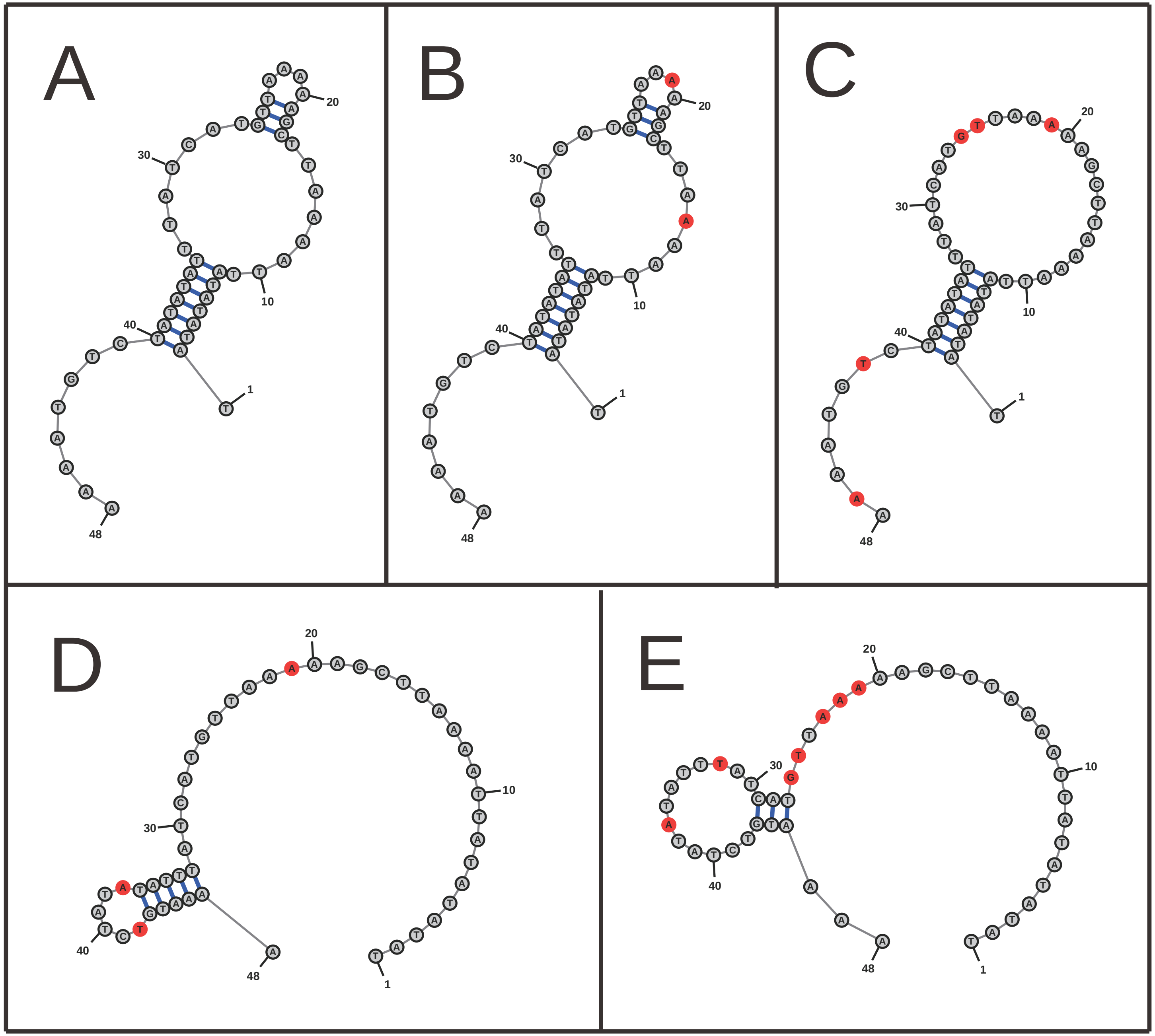

PF3D7_1335100

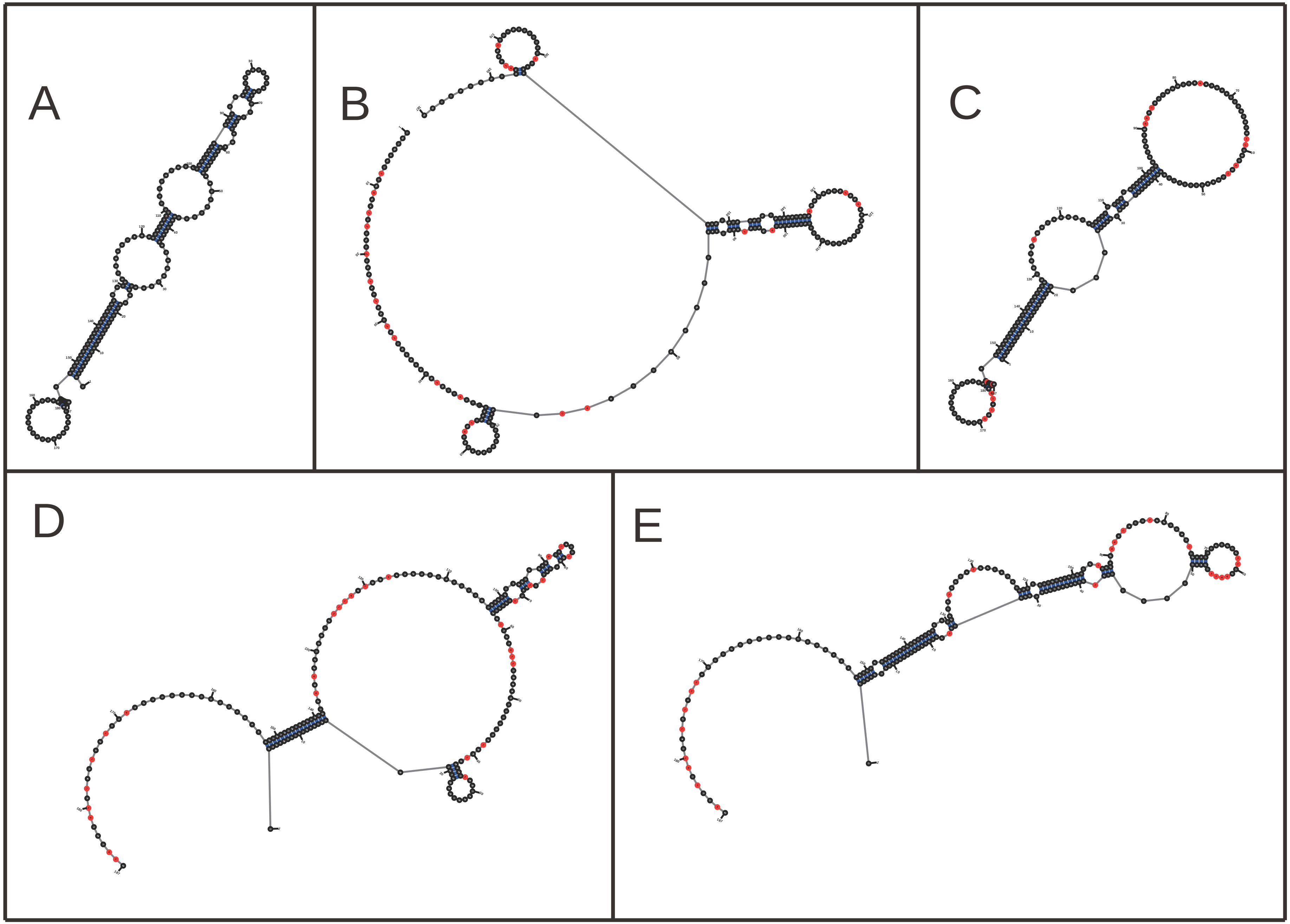

PF3D7_1406800

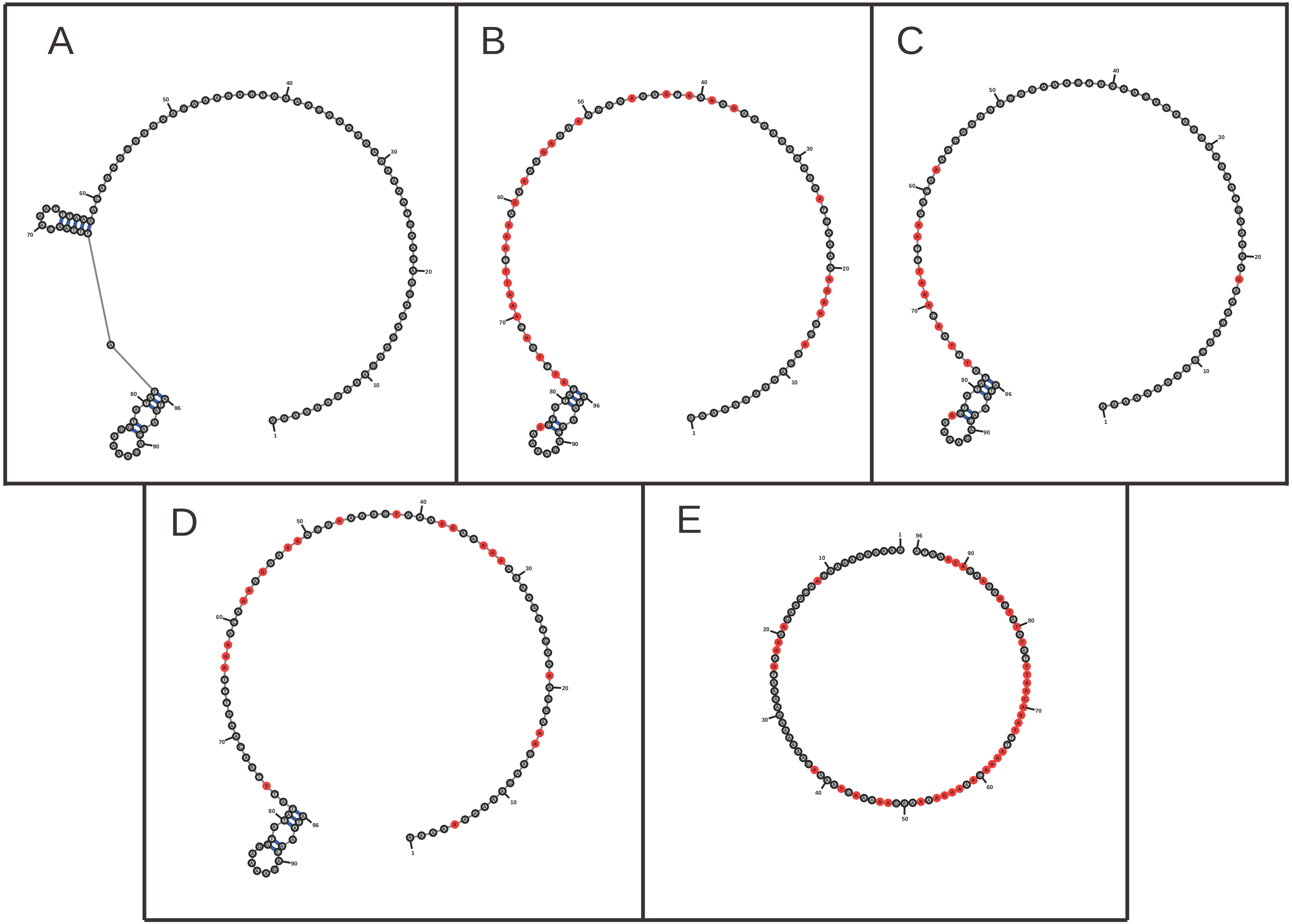

PF3D7_0406200 ring stage

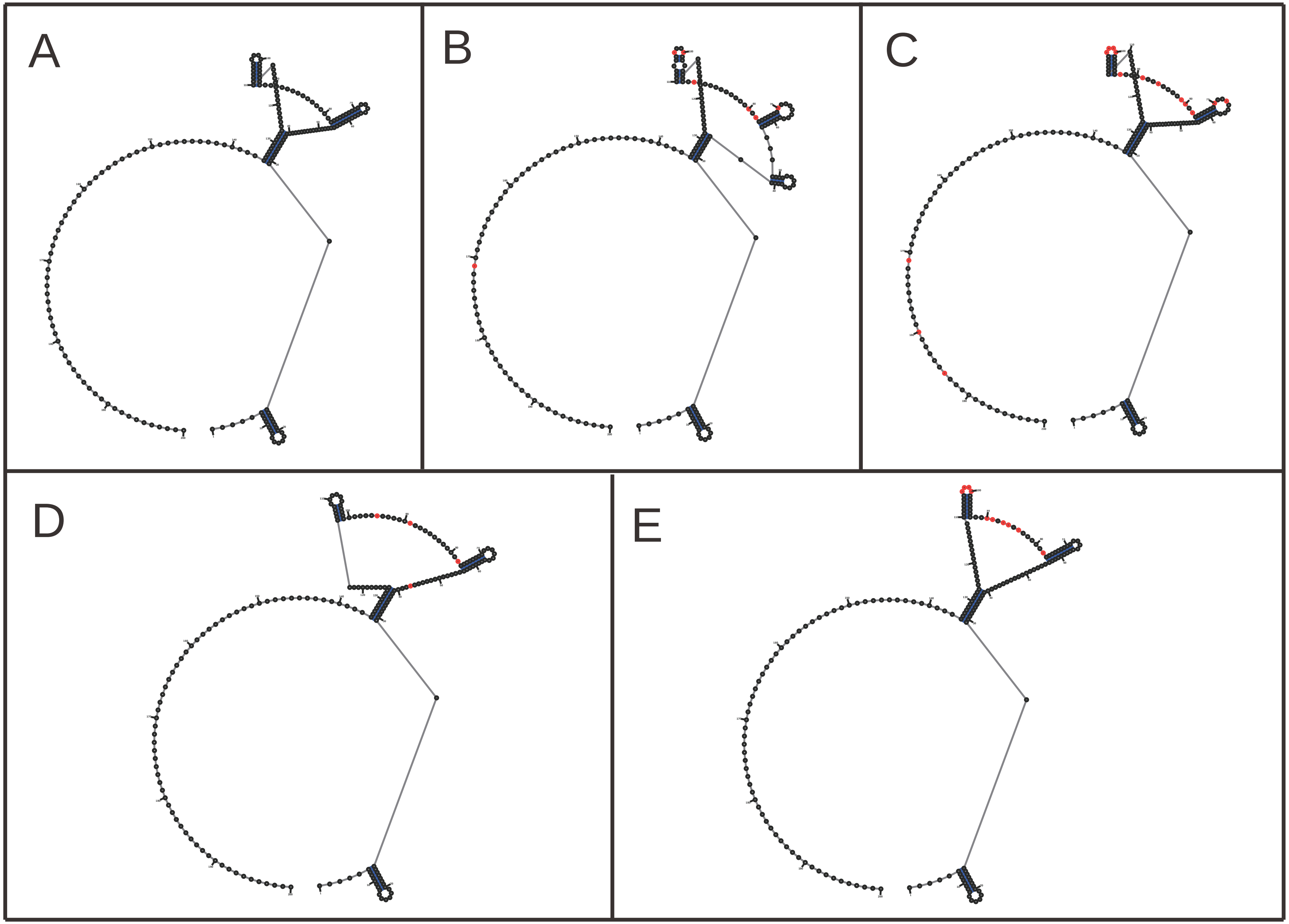

PF3D7_0406200 late stage

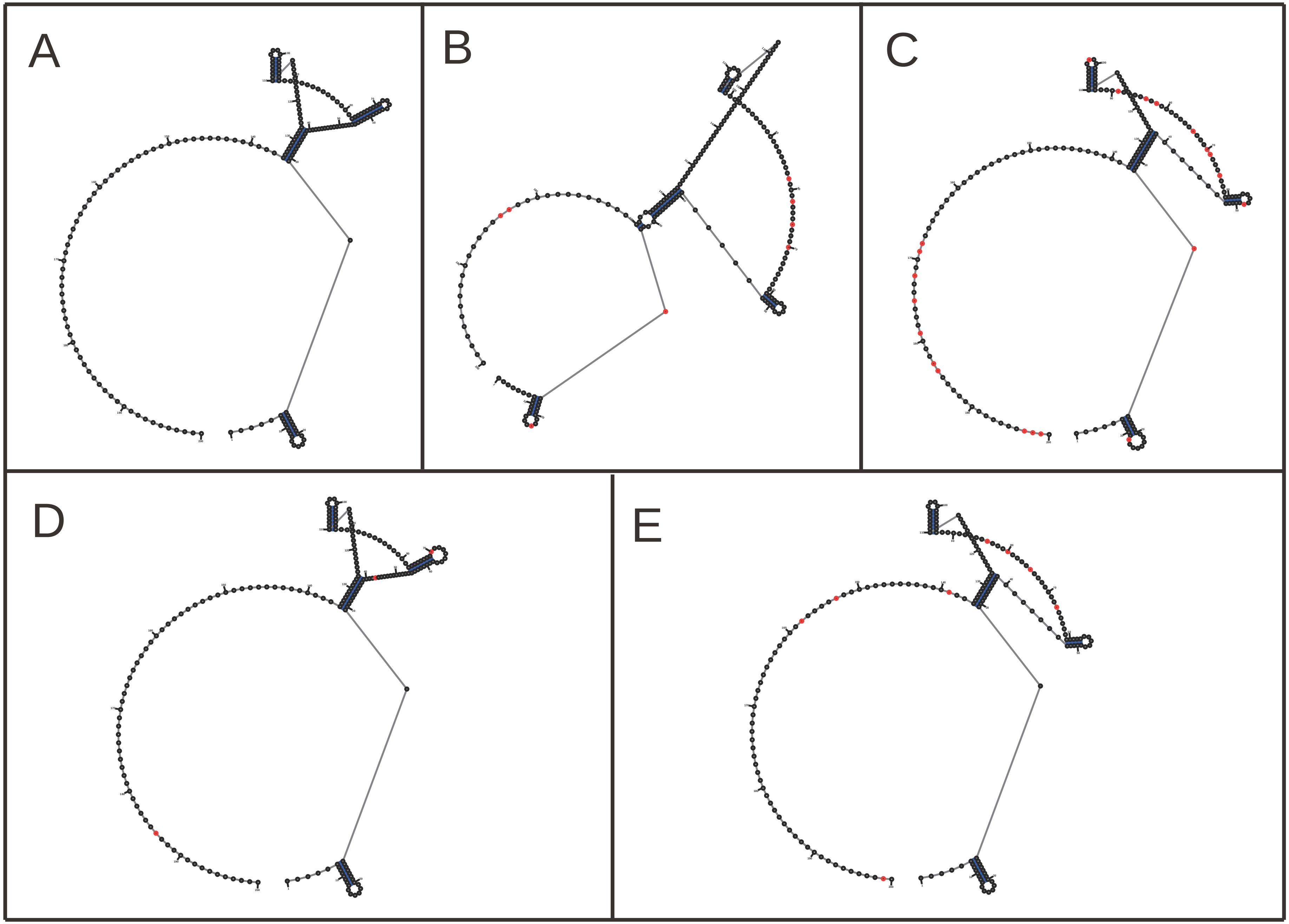

Supplement: Supplementary file 1 [file DataSheet2.PDF]
