# Supplementary material for: RNA Secondary Structurome Revealed Distinct Thermoregulation in Plasmodium falciparum
Source: Front Cell Dev Biol. 2022 Jan 4;9:766532. doi: 10.3389/fcell.2021.766532 (PMC8763798; doi:10.3389/fcell.2021.766532)

Supplementary Figure 1

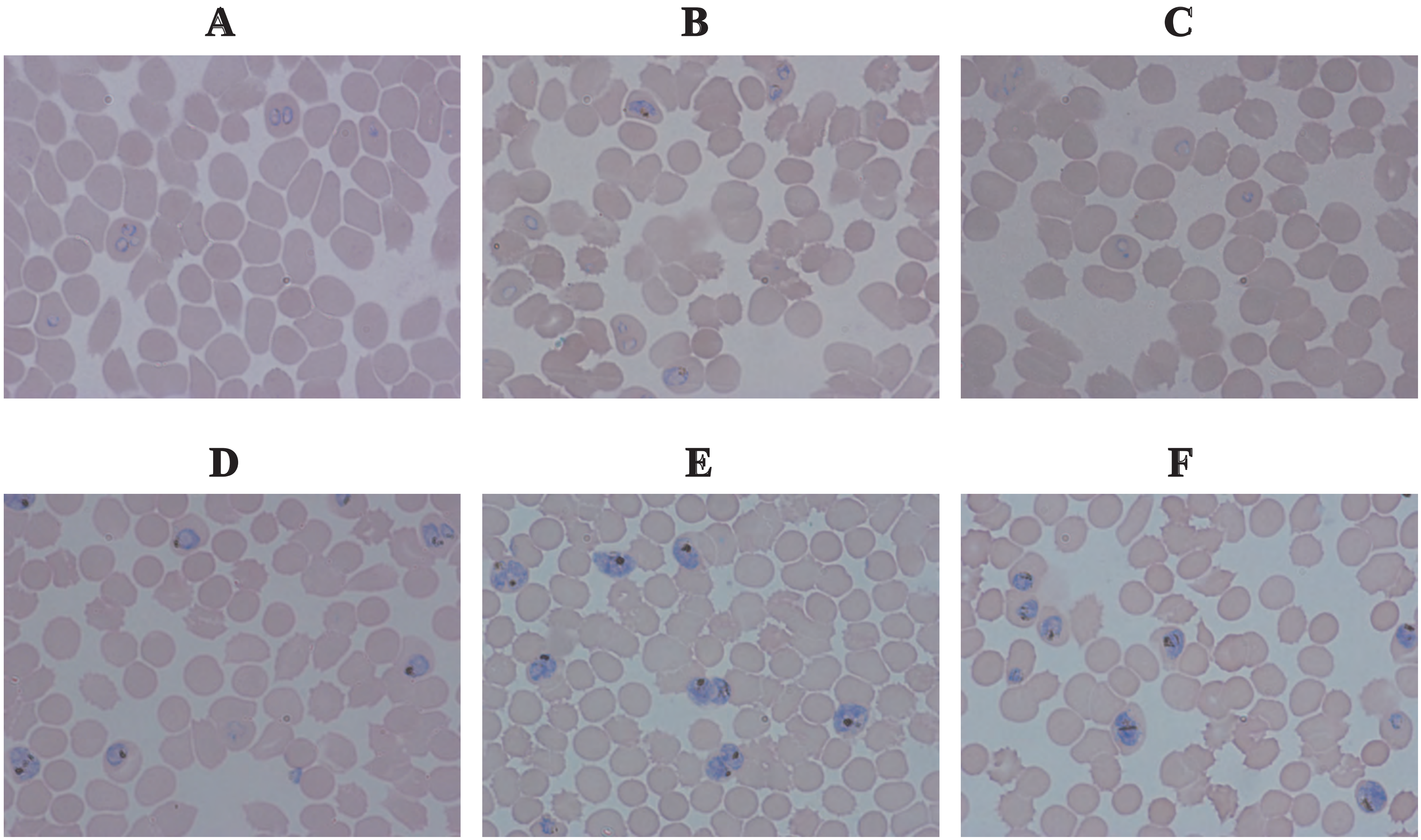

Supplementary Figure 2

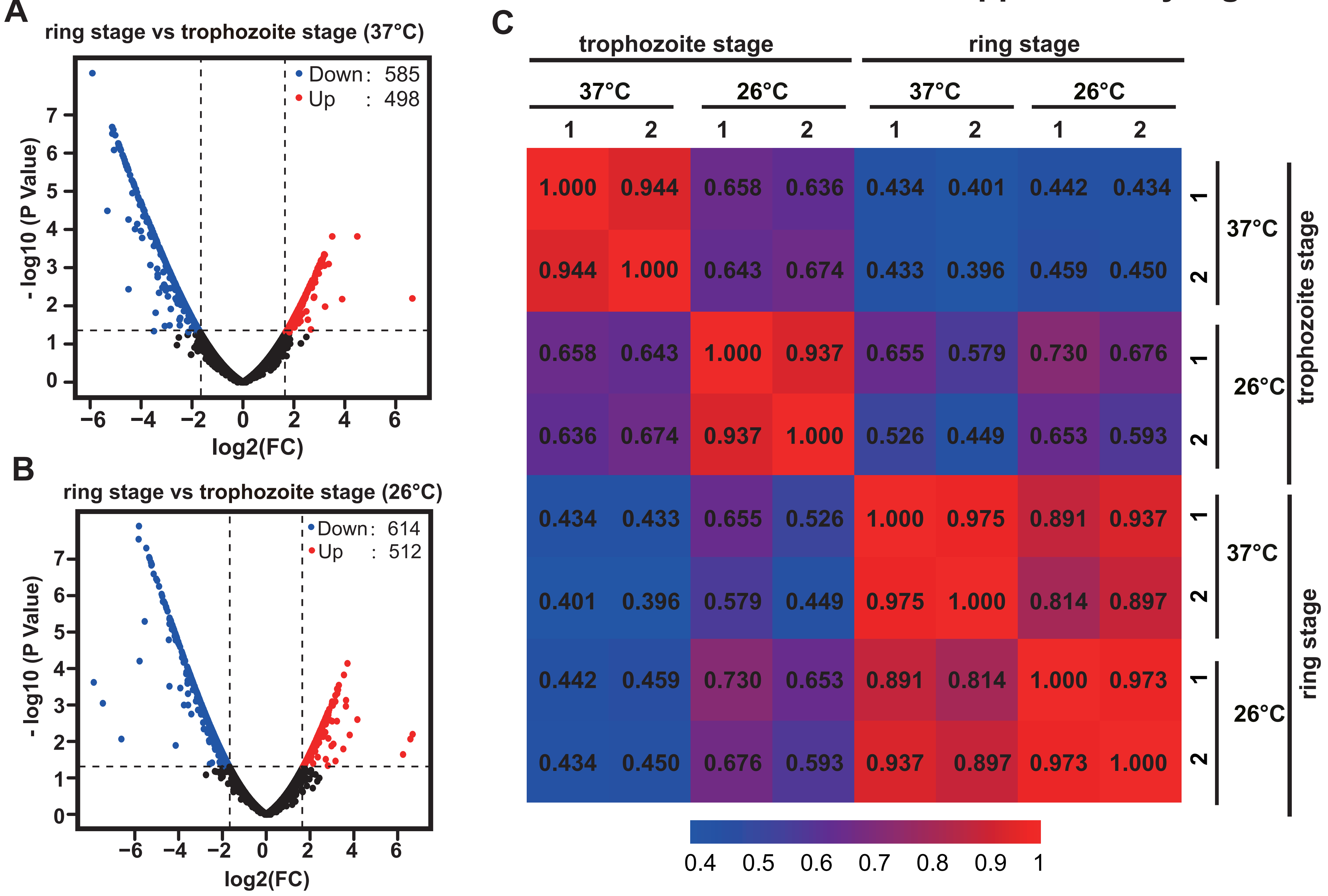

Supplementary Figure 3

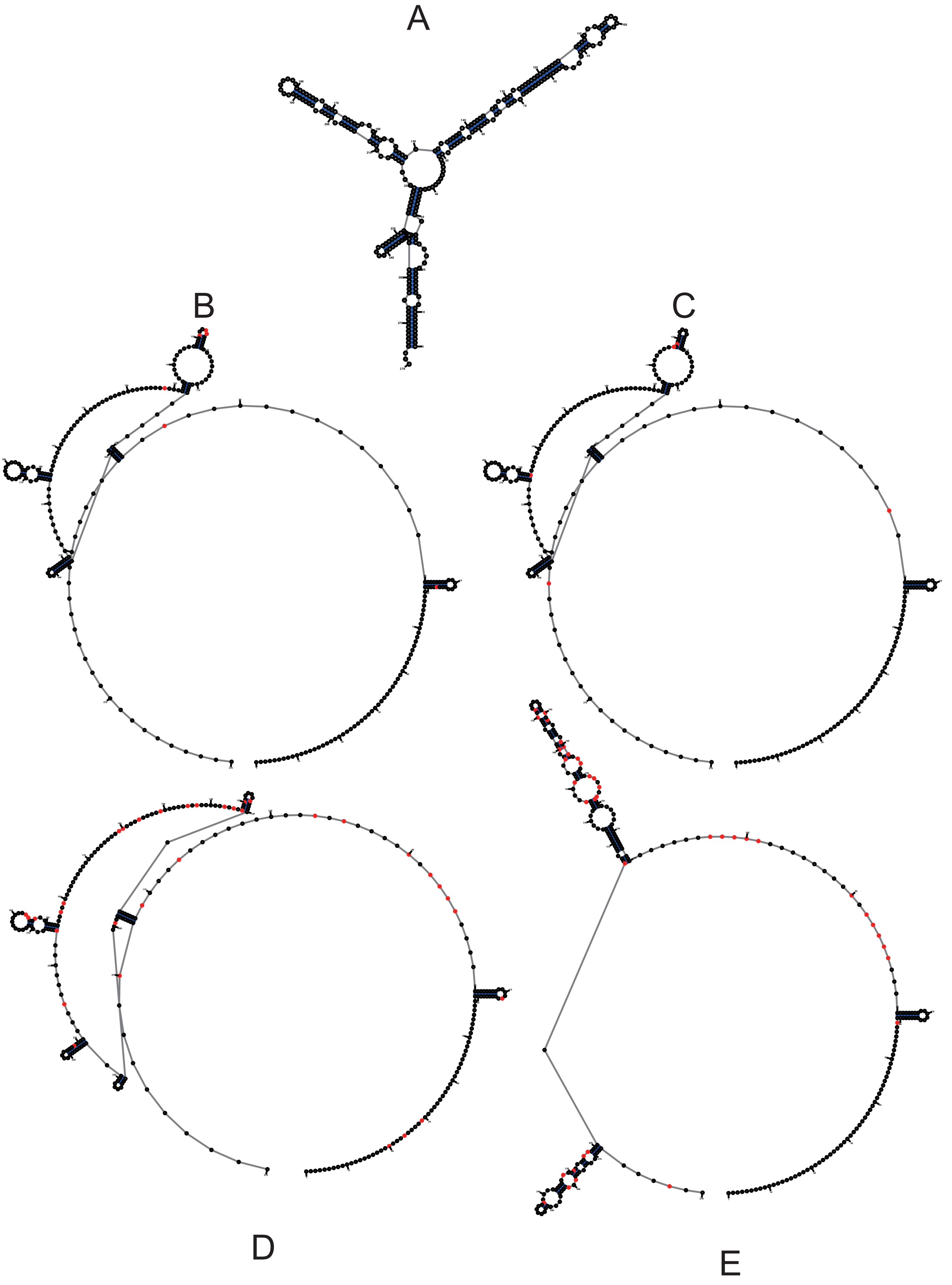

● the red positions in the RNA secondary indicate that icSHAPE scores of this base is no less than 1.5

Supplementary Figure 4

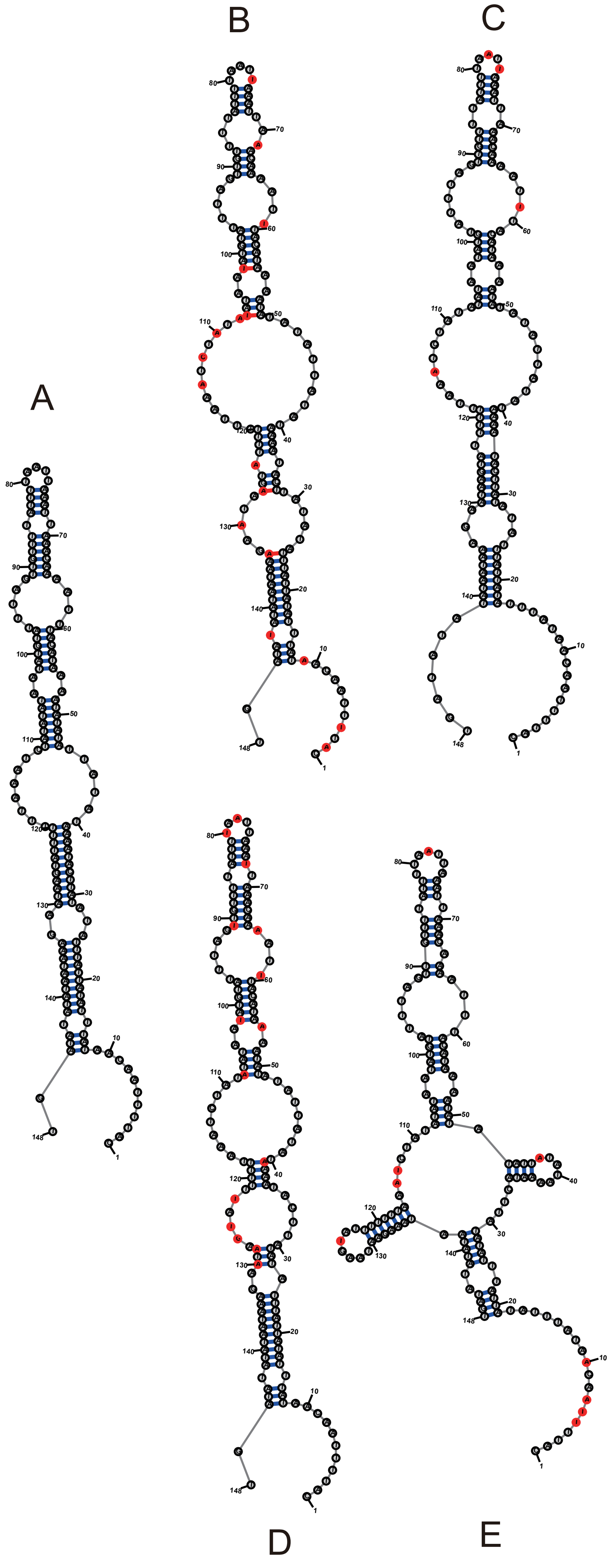

● the red positions in the RNA secondary indicate that icSHAPE scores of this base is no less than 1.5

Supplementary Figure 5

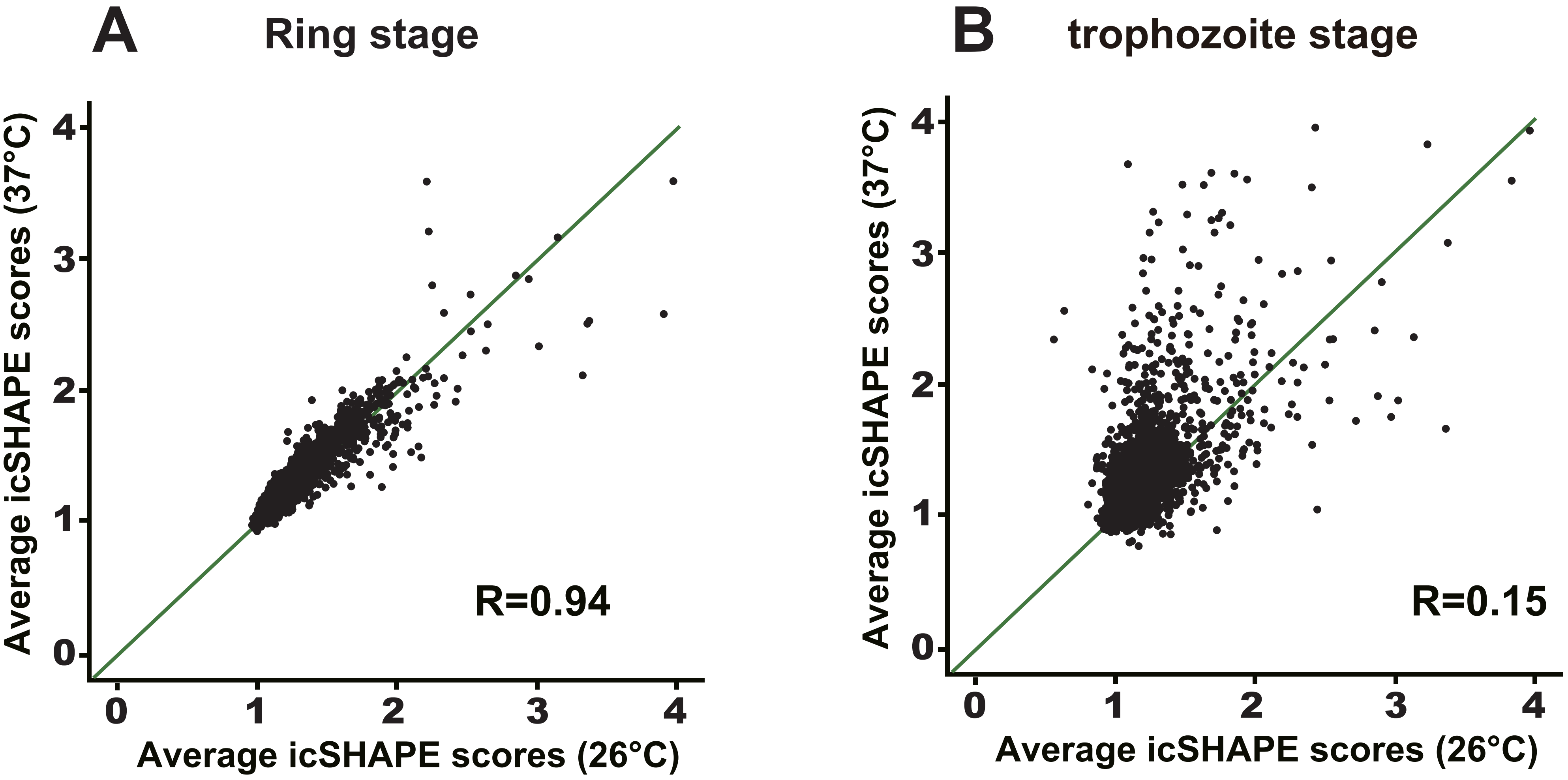

Supplement: Supplementary file 5 [file DataSheet1.PDF]
